# Selective Potassium Chloride Recognition, Sensing, Extraction, and Transport Using a Chalcogen-Bonding Heteroditopic Receptor

**DOI:** 10.1021/jacs.2c05333

**Published:** 2022-08-05

**Authors:** Andrew Docker, Igor Marques, Heike Kuhn, Zongyao Zhang, Vítor Félix, Paul D. Beer

**Affiliations:** † Chemistry Research Laboratory, Department of Chemistry, University of Oxford, Mansfield Road, Oxford OX1 3TA, U. K.; ‡ CICECO—Aveiro Institute of Materials, Department of Chemistry, University of Aveiro, 3810-193 Aveiro, Portugal

## Abstract

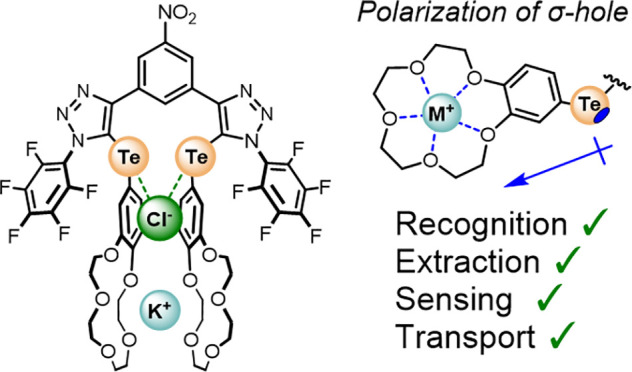

Chalcogen bonding (ChB) is rapidly rising to prominence in supramolecular chemistry as a powerful sigma (σ)-hole-based noncovalent interaction, especially for applications in the field of molecular recognition. Recent studies have demonstrated ChB donor strength and potency to be remarkably sensitive to local electronic environments, including redox-switchable on/off anion binding and sensing capability. Influencing the unique electronic and geometric environment sensitivity of ChB interactions through simultaneous cobound metal cation recognition, herein, we present the first potassium chloride-selective heteroditopic ion-pair receptor. The direct conjugation of benzo-15-crown-5 ether (B15C5) appendages to Te centers in a bis-tellurotriazole framework facilitates alkali metal halide (MX) ion-pair binding through the formation of a cofacial intramolecular bis-B15C5 M^+^ (M^+^ = K^+^, Rb^+^, Cs^+^) sandwich complex and bidentate ChB···X^–^ formation. Extensive quantitative ^1^H NMR ion-pair affinity titration experiments, solid–liquid and liquid–liquid extraction, and U-tube transport studies all demonstrate unprecedented KCl selectivity over all other group 1 metal chlorides. It is demonstrated that the origin of the receptor’s ion-pair binding cooperativity and KCl selectivity arises from an electronic polarization of the ChB donors induced by the cobound alkali metal cation. Importantly, the magnitude of this switch on Te-centered electrophilicity, and therefore anion-binding affinity, is shown to correlate with the inherent Lewis acidity of the alkali metal cation. Extensive computational DFT investigations corroborated the experimental alkali metal cation–anion ion-pair binding observations for halides and oxoanions.

## Introduction

Chalcogen bonding (ChB) is defined as the attractive intermolecular interaction between an electrophilic region of a group 16 atom, commonly referred to as a sigma (σ)-hole, and a nucleophilic Lewis base. While widely acknowledged as a pervasive structural characteristic of main group solid-state chemistry,^1−6^ the exploitation of ChB in the solution phase has only recently been realized. Indeed, seminal applications of ChB, and other noncovalent σ-hole-based interactions, in supramolecular self-assembly,^7−13^ organocatalysis,^14−20^ and transmembrane transport^21−26^ have stimulated intense interest in the field. In the context of anion recognition, employing σ-hole donors in host structural design has frequently demonstrated dramatically augmented anion-binding strength and selectivity behavior relative to hydrogen-bonding host analogues.^27−40^ We have recently shown that ChB donor potency for anion recognition is markedly sensitive to local electronic environments, where electron-withdrawing fluoroaryl substituents operating via an inductive through-bond polarization mechanism effectively modulate ChB donor halide anion affinity and selectivity.^41^ In addition, we have demonstrated redox-switchable on/off ChB anion binding and sensing mediation.^42^ Despite the emergence of this unique ChB anion recognition behavior, the integration of ChB donors in heteroditopic ion-pair receptor design is extremely rare.^43−45^ Notwithstanding the enormous progress made in the field of ion-pair recognition in the last few decades, witnessing receptors exploiting diverse topologies^46−52^ and multitopic recognition modes,^53−64^ heteroditopic receptor systems capable of selective alkali metal cation–chloride anion ion-pair binding are scarce. Seminal examples of selective lithium,^65^ sodium,^66^ rubidium,^67^ and cesium chloride^68,69^ binding have been reported. The selective recognition of potassium chloride, however, is, to the best of our knowledge, unprecedented. This is somewhat surprising given the promise such a selective heteroditopic ion-pair receptor would act as a therapeutic for diseases associated with the misregulation of protein ion channels and as an anticancer agent.^70−72^

**Figure 1:**
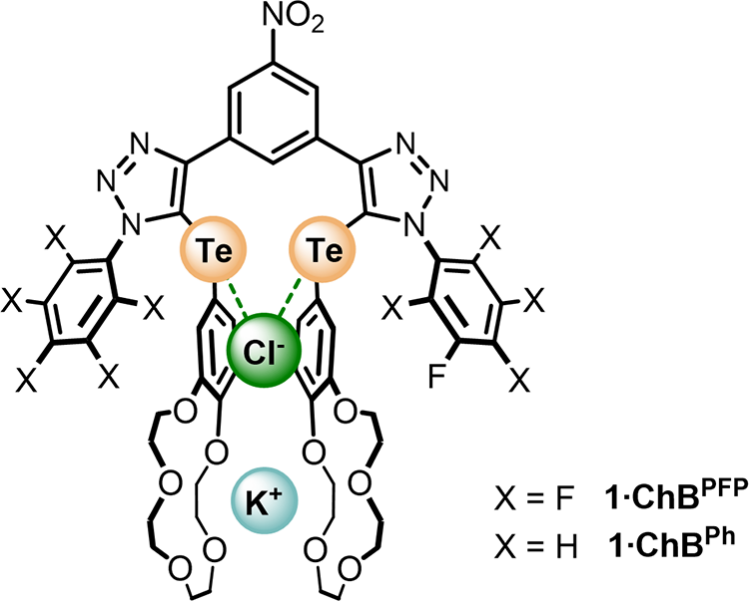
Target chalcogen-bonding heteroditopic receptors, **1·ChB**^**PFP**^ and **1·ChB**^**Ph**^.

Herein, we report the first KCl selective ChB heteroditopic ion-pair receptor, **1·ChB**^**PFP**^, consisting of a 3,5-bis-tellurotriazole nitro-benzene scaffold (Figure 1), with electron-deficient perfluorophenyl substituents and benzo-15-crown-5 (B15C5) units directly appended to the tellurium-incorporated triazoles. We demonstrate that the anion affinity of the receptor is dependent on the formation of a cobound bis-B15C5 potassium cation-induced cofacial intramolecular sandwich, which not only serves to conformationally preorganize the receptor but also induces through-bond polarization of the proximal Te centers that, in effect, switch on the Lewis acidity of the Te σ-hole ChB donors via a noncovalent cooperativity mechanism. The synergy between cation and anion recognition events is responsible for the marked KCl selectivity of **1·ChB**^**PFP**^, over all other group 1 metal chlorides, and underpins the receptor’s ability to perform potassium chloride-selective solid–liquid and liquid–liquid extraction and membrane transport.

## Results and Discussion

### Synthesis of Chalcogen-Bonding Ion-Pair Receptors

**Figure 2:**
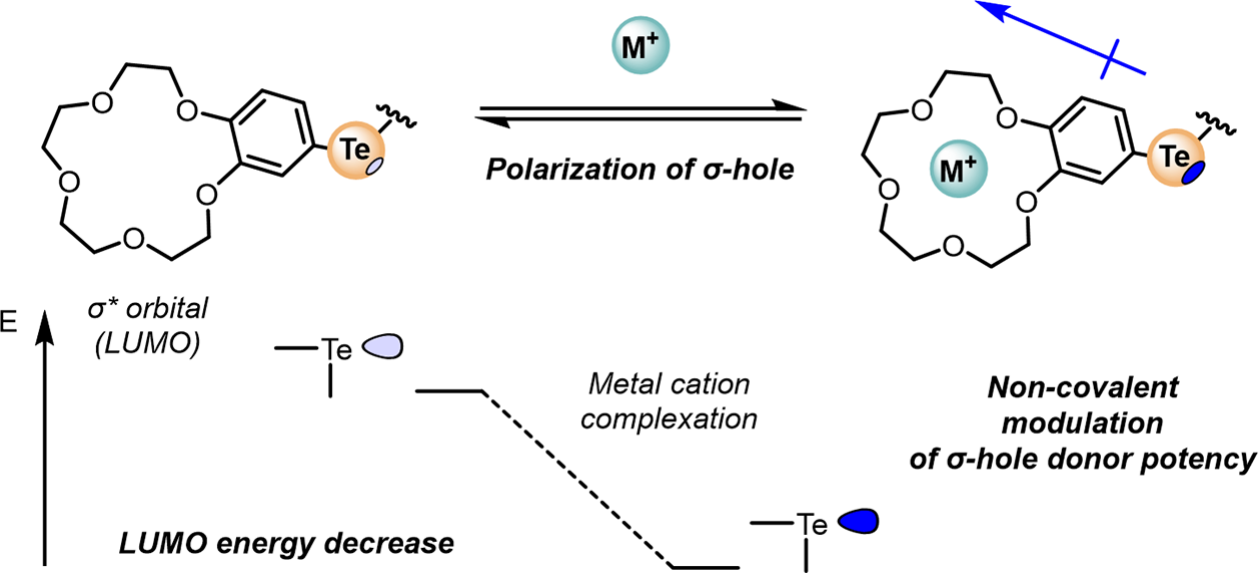
Cartoon representation of the orbital and electrostatic effects of alkali metal cation crown ether complexation.

The target ChB heteroditopic ion-pair receptor design featured an electron-deficient nitro-benzene aromatic scaffold integrated with chelating ChB donor 3,5-bis-telluro-triazole perfluorophenyl or phenyl motifs, wherein each constituent Te atom is directly covalently appended to a B15C5 group. It was anticipated that an appropriately sized alkali metal cation would induce the formation of a cofacial 1:1 stoichiometric intramolecular bis-B15C5 sandwich complex, conformationally preorganizing the receptor to form a bidentate ChB donor cleft for halide anion recognition. Importantly, the conjugated nature of the receptor design serves to relay the through-bond inductive electronic influences of (i) electronic-withdrawing variation of the tellurium-triazole-appended aryl substituents and (ii) alkali metal cation bis-B15C5 complexation, activating the efficacy of σ-hole ChB donor atom potency for anion and ion-pair recognition (Figure 2).

**Scheme 1:**
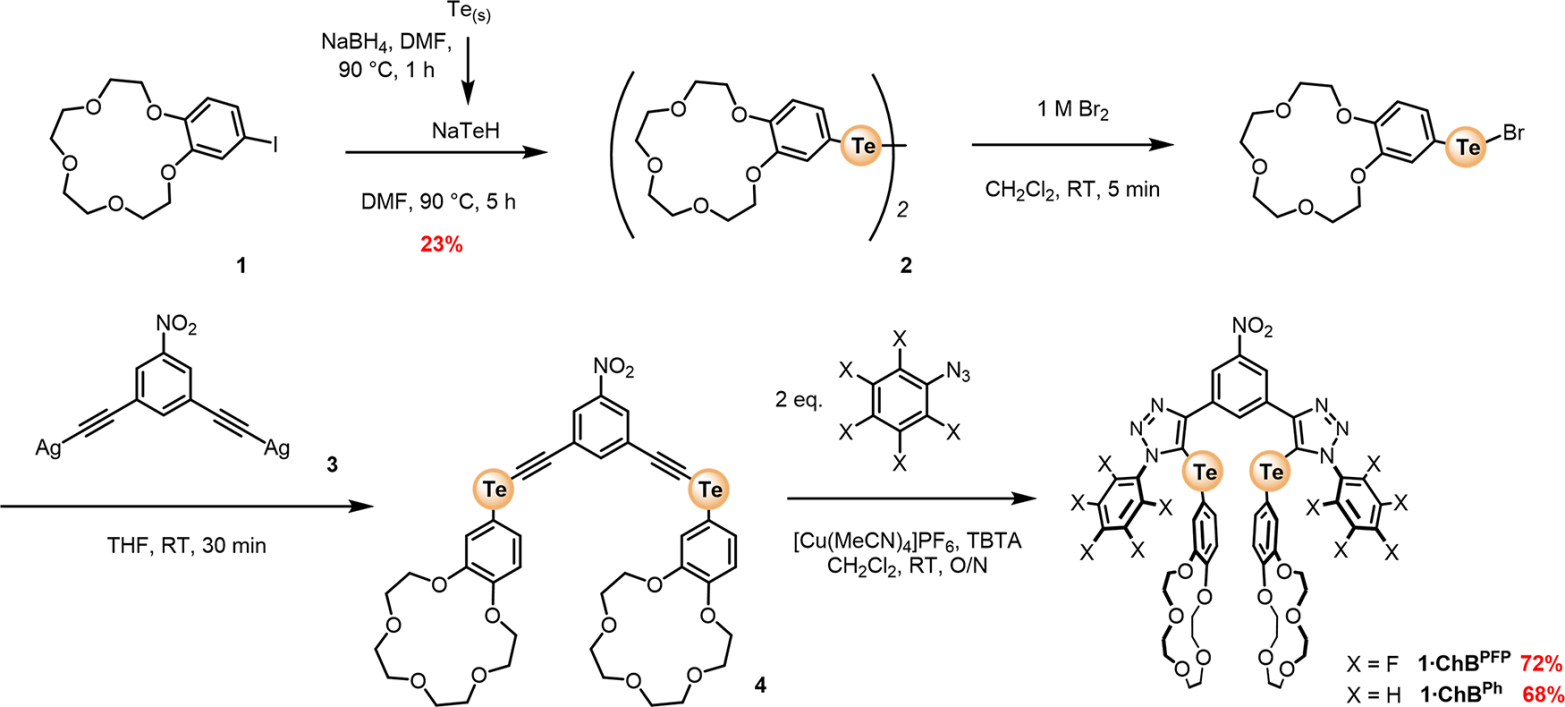
Synthesis of B15C5 Ditelluride and CuAAC-Mediated Synthesis of **1·ChB**^**PFP**^ and **1·ChB**^**Ph**^

The ChB heteroditopic receptors were synthesized according to a copper(I)-catalyzed alkyne-azide cycloaddition CuAAC methodology (Scheme 1). The requisite B15C5-appended, tellurium-functionalized alkyne precursors were prepared according to Scheme 1. A nucleophilic substitution reaction between freshly generated NaTeH and iodo-arene **1** followed by an aerial oxidation procedure afforded the requisite ditelluride **2** in 23% yield after column chromatography. The synthesis of the bis-telluoro-B15C5 alkyne was achieved by careful treatment of ditelluride **2** with a 1 M Br_2_ CH_2_Cl_2_ solution, forming the corresponding organotellurium bromide, which was reacted immediately with a THF suspension of bis-silver-acetylide **3**. The generated bis-alkyne **4** target was used in a subsequent CuAAC reaction with 2 equiv of the appropriate aryl azide in the presence of catalytic TBTA in anhydrous dichloromethane. Subsequent aqueous workup procedures of the reaction mixtures and purification of the crude materials by column chromatography gave the novel heteroditopic receptors **1·ChB**^**PFP**^ and **1·ChB**^**Ph**^ in 72 and 68% yields, respectively, characterized by ^1^H, ^13^C, and ^125^Te NMR and high-resolution electrospray ionization mass spectrometry (ESI-MS).

### Anion and Ion-Pair Recognition Studies

**Figure 3:**
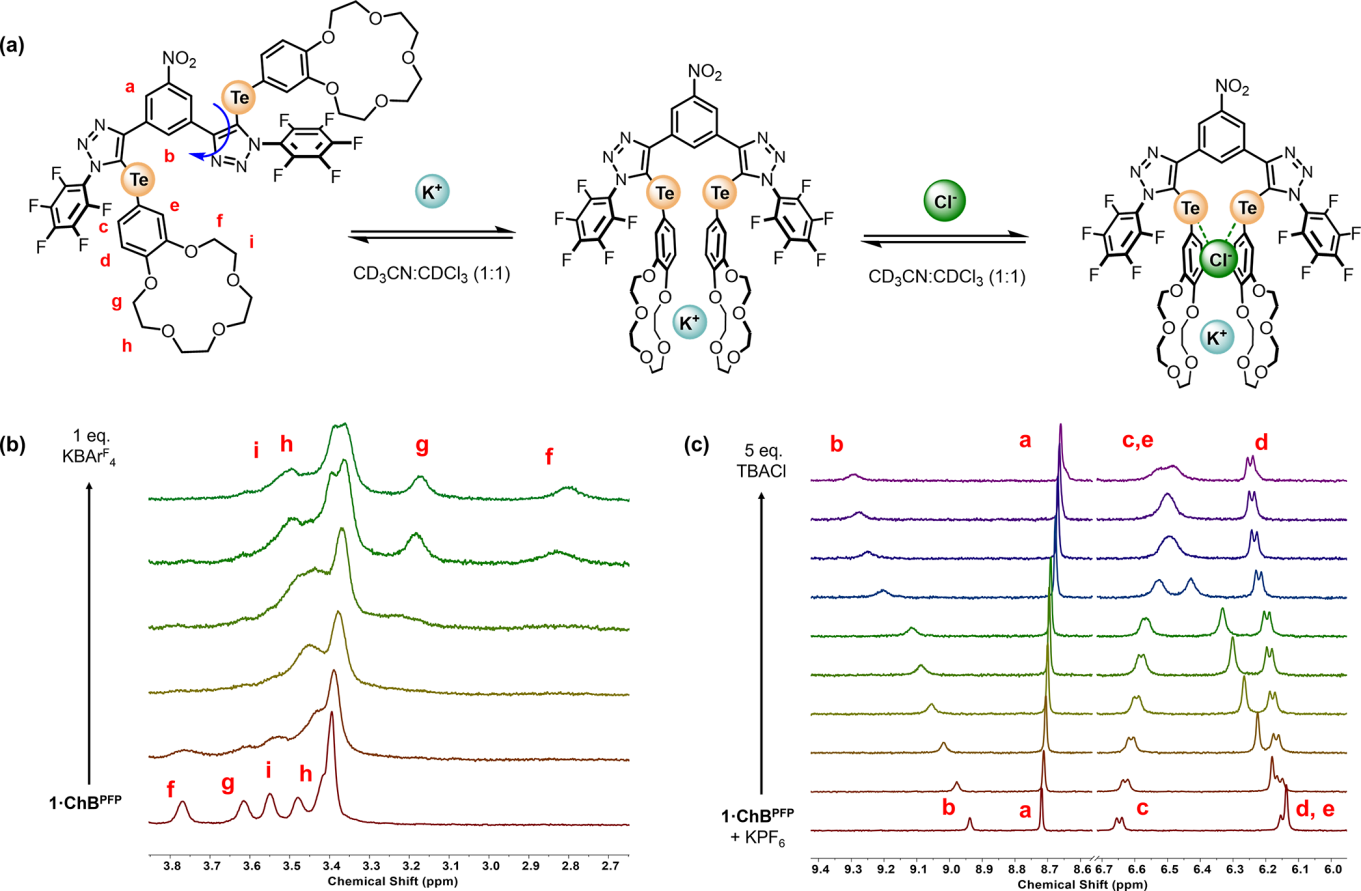
(a) Potassium chloride **1·ChB**^**PFP**^ binding equilibria. ^1^H NMR titration experiments of (b) KBAr_4_^F^ and **1·ChB**^**PFP**^ (c) TBACl and **1·ChB**^**PFP**^ in the presence of 1 equiv of KPF_6_ (CD_3_CN/CDCl_3_ 1:1 (v/v), 500 MHz, 298 K).^73^

Initially, the potassium cation recognition properties of **1·ChB**^**PFP**^ were investigated via ^1^H NMR titration studies conducted by adding increasing equivalents of K^+^ as the highly organic solvent-soluble tetrakis 3,5-bis(trifluoromethyl)phenyl borate (BAr_4_^F–^) salt to a 1:1 CD_3_CN/CDCl_3_ (v/v) solution of the receptor (Figure 3a,b). The addition of the potassium cation induced significant perturbations and broadening of the resonances associated with the crown ether methylene and aromatic regions. Indeed, the broadening of receptor proton signals c–h was so pronounced as to result in their disappearance until 1 equiv of KBAr_4_^F^ had been administered, after which no further changes were observed, suggesting the formation of a highly stable 1:1 stoichiometric host/guest complex (*K*_a_ > 10^5^ M^–1^). Inspection of the ^1^H NMR spectrum revealed several key features concerning the nature of the potassium complex, specifically the dramatic ca. 1 and 0.5 ppm upfield shifts of CH_2_ signals f and h of the polyether chain, indicating strong shielding effects from a proximal aromatic ring current, supporting the formation of an intramolecular cofacial K^+^ bis-B15C5 sandwich complex (see the Supporting Information for further details).^74−76^ Importantly, KBAr_4_^F^ titration experiments with **1·ChB**^**Ph**^ elicited similar spectroscopic changes, indicating the formation of an analogous K^+^ bis-B15C5 sandwich complex.

**Figure 4:**
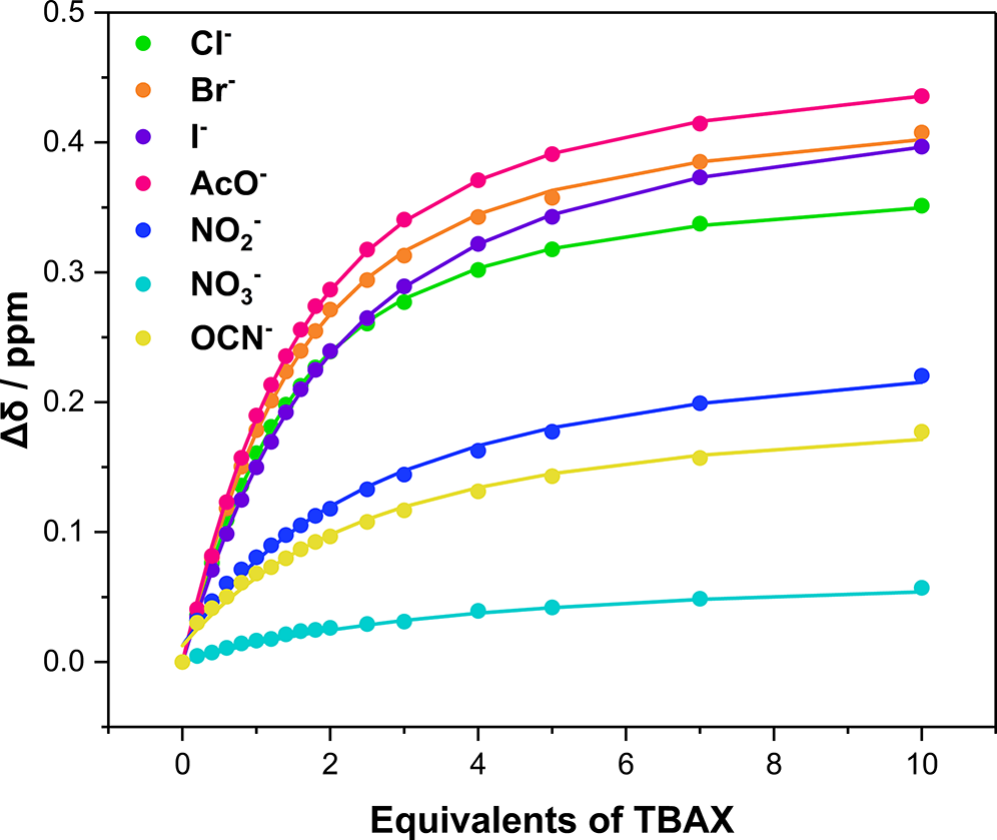
Anion-binding isotherms for **1·ChB**^**PFP**^ in the presence of 1 equiv of KPF_6_ (CD_3_CN/CDCl_3_ 1:1 (v/v), 500 MHz, 298 K).

The ion-pair receptor capabilities of **1·ChB**^**PFP**^ and **1·ChB**^**Ph**^ were investigated by ^1^H NMR anion titration experiments conducted in the presence of equimolar KPF_6_ (Figure 3c). A variety of anions, added as their tetrabutylammonium salts, induced progressive downfield shifts of internal aromatic signal b. Importantly, it was observed that the diagnostic features of the ^1^H NMR spectrum associated with the K^+^B15C5 sandwich complex, persisted upon anion addition, indicating concomitant ion-pair binding. Bindfit analysis^77^ of the anion-induced chemical shift perturbations (Figure 4) determined 1:1 stoichiometric host/guest association constants for the range of halide and oxoanions investigated (Table 1). Notably, anion titration experiments conducted in the absence of a K^+^ source elicited no chemical shift perturbations, indicating that a cobound potassium cation is crucial for switching on the anion recognition capabilities of both receptors. For **1·ChB**^**PFP**^, inspection of Table 1 reveals strong halide and acetate binding, in particular chloride that is bound with an association constant (*K*_a_) of 1198 M^–1^. The observed chloride selectivity, over anions such as acetate, is particularly impressive considering that affinity trends in simple acyclic hydrogen bond donor anion receptor systems are typically dictated by intrinsic anion basicity. In comparison to **1·ChB**^**PFP**^, the halide association constant values for **1·ChB**^**Ph**^ are considerably diminished, by nearly an order of magnitude. Saliently, these results indicate that the major determinant in thermodynamic stability of the ion-pair complex is the formation of potent ChB–anion interactions and not a consequence of nonspecific electrostatic interactions between the cationic K^+^ receptor complex and the anion guest species.

**Table 1:** Anion Association Constants for **1·ChB**^**PFP**^ and **1·ChB**^**Ph**^ from ^1^H NMR Titration Experiments (1:1 CD_3_CN/CDCl_3_ (v/v), 500 MHz, 298 K)

	Anion association constant (*K*_a_, M^-1^) in the presence of equimolar KPF_6_^a^	
Anion^b^	**1·ChB**^**PFP**^	**1·ChB**^**Ph**^
Cl^–^	1198	128
Br^–^	1080	190
I^–^	709	93
AcO^–^	1020	d
NO_3_^–^	276	d
NO_2_^–^	458	d
OCN^–^	486	d
ClO_4_^–^	c	d

a Determined from Bindfit analysis, monitoring signal b, error < 5%.

b Anions added as their tetrabutylammonium salts.

c No binding.

d Not performed.

Attention was subsequently directed toward investigating the role of the nature of the alkali metal cation in the formation of the bis-B15C5 sandwich complex and its effects on the MX ion-pair recognition properties of the receptor. Inspection of the ^1^H NMR spectra of **1·ChB**^**PFP**^ with equimolar potassium, rubidium, or cesium perchlorate in 1:1 CD_3_CN/CDCl_3_ (v/v) evidenced similar diagnostic chemical shift changes of the crown ether methylene region associated with the formation of the K^+^ complex, indicating both the larger Rb^+^ and Cs^+^ cations form the 1:1 stoichiometric intramolecular sandwich complex. Analogous complexation experiments conducted with the sequential addition of 1 and 2 equiv of LiClO_4_ or NaClO_4_ indicated very minor resonance perturbations (<0.05 ppm), suggesting that smaller Li^+^ and Na^+^ are each bound by a single B15C5 forming 1:2 host/M^+^ stoichiometric complexes that were subsequently confirmed by qualitative ^1^H NMR cation titration experiments (see Figure S17).

When complexed with 1 equiv of Rb^+^ or Cs^+^, the addition of increasing chloride equivalents induced similar significant downfield perturbations of the **1·ChB**^**PFP**^ receptor’s internal aromatic signal b. In contrast, in the presence of 1 equiv of lithium or sodium perchlorate, no downfield shift of aromatic signal b was observed upon the addition of chloride. Furthermore, minor crown-ether-based perturbations observed, interpreted as a result of Li^+^ or Na^+^ complexation, were lost upon addition of 1 equiv of chloride, indicating salt-recombination precipitation by direct electrostatic interactions between the chloride anion and either the lithium or sodium cation. Bindfit analysis of the resultant binding isotherms revealed that the chloride affinity increases with decreasing group 1 metal cation radius, K^+^ > Rb^+^ > Cs^+^ (Table 2).

**Table 2:** Chloride Association Constants for **1·ChB**^**PFP**^ from ^1^H NMR Titration (1:1 CD_3_CN/CDCl_3_ (v/v), 500 MHz, 298 K)

Chloride association constant (*K*_a_, M^-1^) of **1·ChB**^**PFP**^ in the presence of equimolar MClO_4_^a^				
Li^+^	Na^+^	K^+^	Rb^+^	Cs^+^
b	b	1100	742	605

a Determined from Bindfit analysis, monitoring signal b, error < 10%.

b ^1^H NMR evidence demonstrates quantitative cation decomplexation and salt recombination.

### Single-Crystal Diffraction Studies of Ion-Pair Complexes

**Figure 5:**
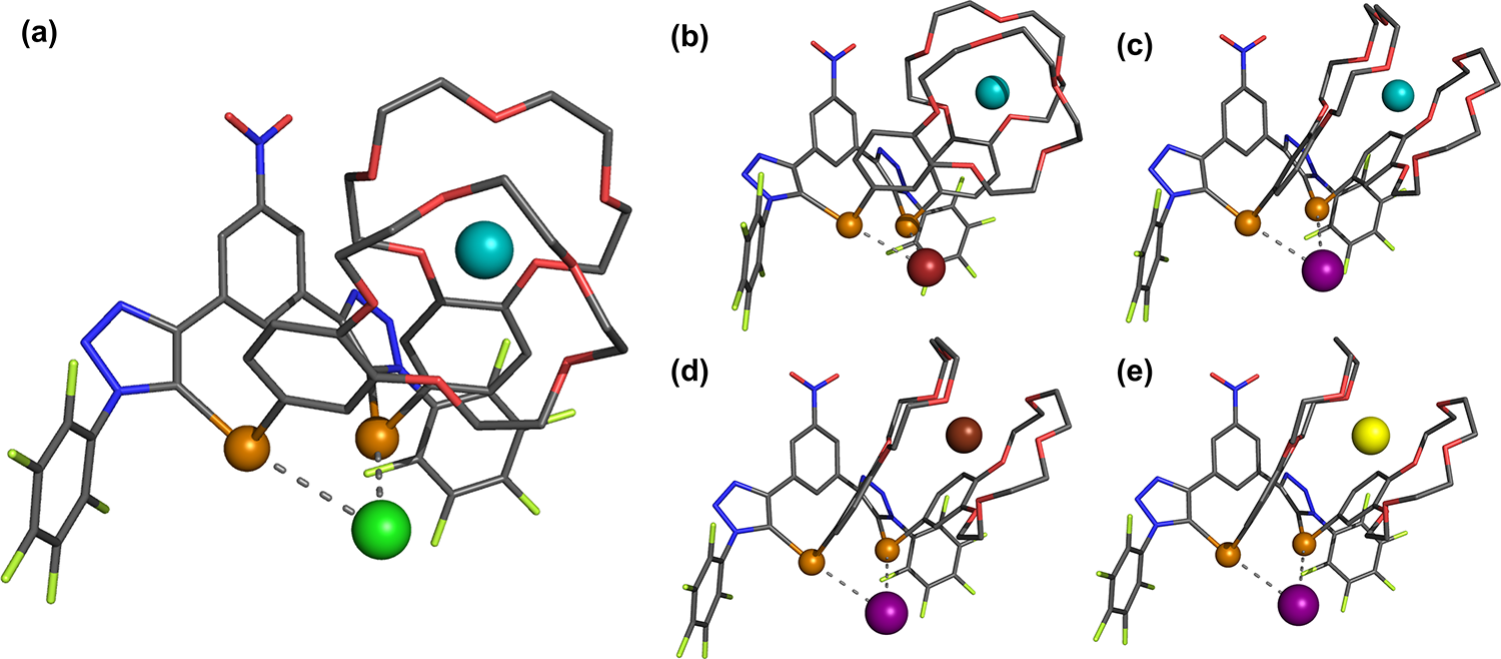
Solid-state structures of **1·ChB**^**PFP**^ complexed with (a) KCl, (b) KBr, (c) KI, (d) RbI, and (e) CsI (solvent molecules and hydrogen atoms are omitted for clarity). Grey = carbon, blue = nitrogen, red = oxygen, light green = fluorine, orange = tellurium, cyan = potassium, brown = rubidium, yellow = cesium, green = chlorine, dark red = bromine, purple = iodine.

Further insight into the ion-pair binding behavior of **1·ChB**^**PFP**^ was provided by solid-state characterization of the ion-pair receptor complexes. Crystals suitable for X-ray structure determination of the KCl, KBr, KI, RbI, and CsI complexes were obtained. Concordant with ^1^H NMR titration studies, potassium, rubidium, and cesium cations are complexed via a cofacial B15C5 sandwich complex, and the halide counteranions exhibit bifurcated chalcogen bond formation (Figure 5). Inspection of the summarized tellurium–halide interaction distances in Table 3 revealed short contacts, considerably shorter than the sum of their van der Waals radii.

**Table 3:** Selected Interaction Distances Obtained from Crystal Structure Determination of **1·ChB**^**PFP**^ Ion-Pair Complexes

Ion-pair	Interatomic distances (Te···X^–^ (Å))^a^	Contraction of van der Waals radii (%)
KCl	3.099(15), 3.520(15)	81, 92
KBr	3.245(7), 3.579(7)	83, 92
KI	3.460(8), 3.889(8)	86, 96
RbI	3.467(6), 3.871(5)	86, 96
CsI	3.478(6), 3.904(6)	86, 97

a Calculated uncertainties are in parentheses.

### Alkali Metal Chloride Solid–Liquid and Liquid–Liquid Ion-Pair Extraction Studies

**Figure 6:**
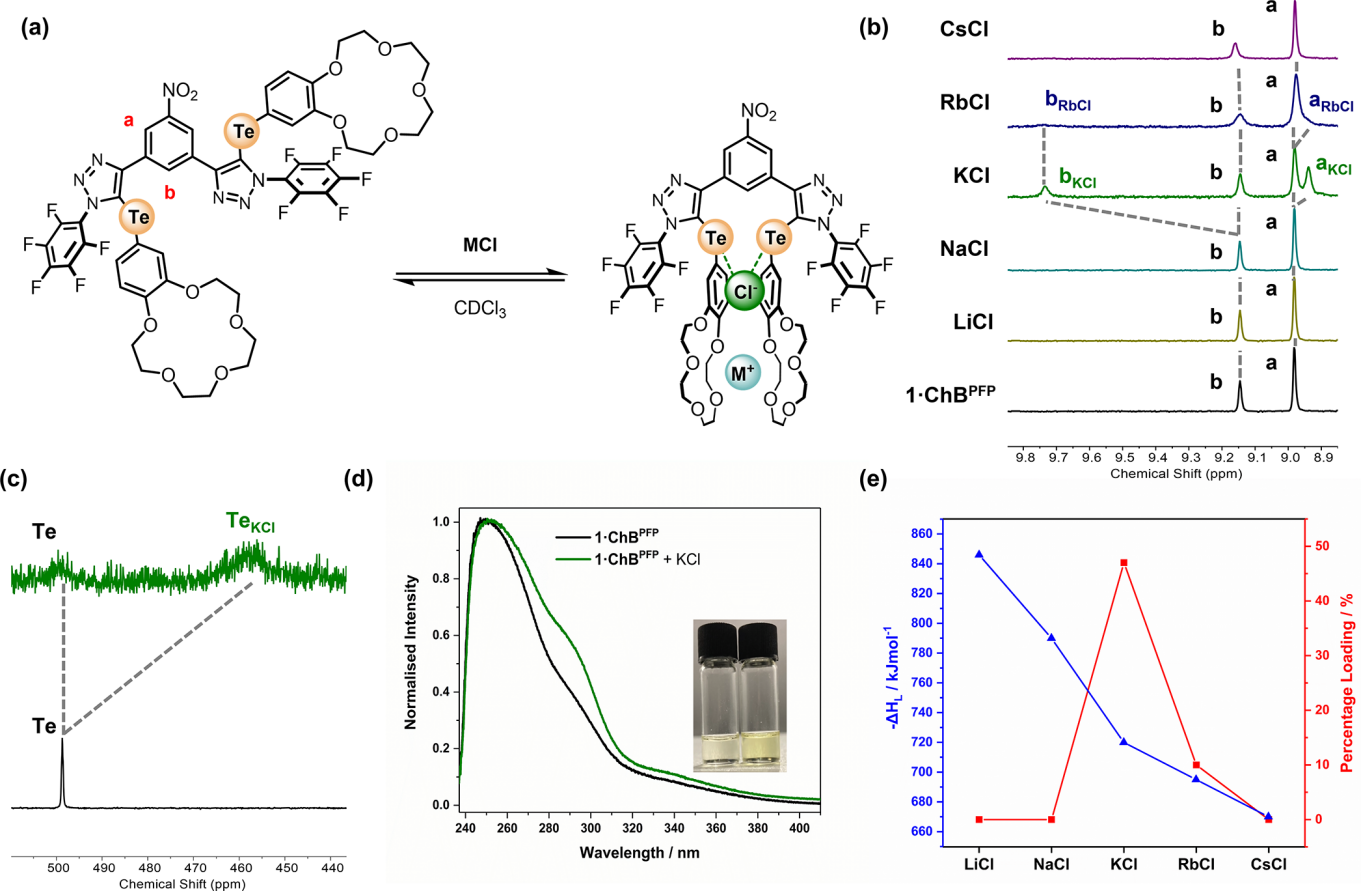
(a) Ion-pair binding equilibrium for **1·ChB**^**PFP**^. Pre and post-SLE: (b) ^1^H NMR spectra (CDCl_3_, 500 MHz, 298 K), (c) ^125^Te NMR spectra (CDCl_3_, 126 MHz, 298 K), (d) UV–vis spectra (10^–4^ M, CDCl_3_); inset: picture of pre- and post-extraction solutions. (e) Plot showing receptor loading (%) (red) versus MCl lattice energy.

Encouraged by the KCl ion-pair selectivity of **1·ChB**^**PFP**^ as evidenced by the quantitative ^1^H NMR binding studies, attention was directed toward investigating the receptor **1·ChB**^**PFP**^ as an extraction agent for KCl under solid–liquid extraction (SLE) and liquid–liquid extraction (LLE) conditions. In a typical SLE experiment, a CDCl_3_ solution of **1·ChB**^**PFP**^ (colorless) was layered over a microcrystalline sample of KCl (Figure 6a) and stirred for 10 min. The ^1^H NMR spectrum of the resultant yellow post-extraction solution revealed new sets of resonances in addition to those corresponding to the free receptor (Figure 6b). Control experiments revealed that the complexation of KCl is slow on the NMR time scale and the new signals correspond to those of the **1·ChB**^**PFP**^·KCl complex (see Supporting Information p36 for further details). Notably, the internal aromatic signal, proximal to the ChB donor cleft, of the potassium chloride complex, *b*_KCl_, exhibits a dramatic 0.6 ppm downfield shift relative to the same proton, b, of the free receptor. Integration of these respective signals enabled the calculation of the KCl loading percentage, from which it was determined that 47% of **1·ChB**^**PFP**^ was complexed with potassium chloride. Despite its low abundance, ^125^Te nuclei are highly sensitive to solution-phase noncovalent interaction formation and provided a further opportunity to investigate their ion-pair binding behavior. A comparison of the pre- and post-extraction ^125^Te NMR spectra (Figure 6c), similarly to ^1^H NMR, revealed the appearance of a new tellurium signal, a 45 ppm upfield shift relative to free **1·ChB**^**PFP**^. The observed color change upon KCl extraction also prompted UV–vis studies of the extraction process (Figure 6d), in which it was demonstrated that the naked eye response relies on concomitant K^+^ and Cl^–^ binding, while no measurable response is exhibited in the sole presence of either a potassium cation or chloride anion source with a noncoordinating counterion. Analogous SLE experiments conducted with other group 1 metal chlorides (MCl, M = Li, Na, Rb, Cs) exhibited a starkly different result. Most notably, in comparison to other group 1 metal chlorides, **1·ChB**^**PFP**^ exhibits remarkable selectivity for KCl, demonstrating either no extraction as in the case of lithium, sodium, and cesium or only 10% receptor loading for rubidium (Figure 6e). Importantly, a comparison of the extraction performance versus the lattice enthalpy (Δ*H*_L_) reveals that despite a reduced energetic penalty of lattice dissolution for RbCl relative to KCl, potassium chloride is extracted over four times more efficiently than its rubidium congener.

**Figure 7:**
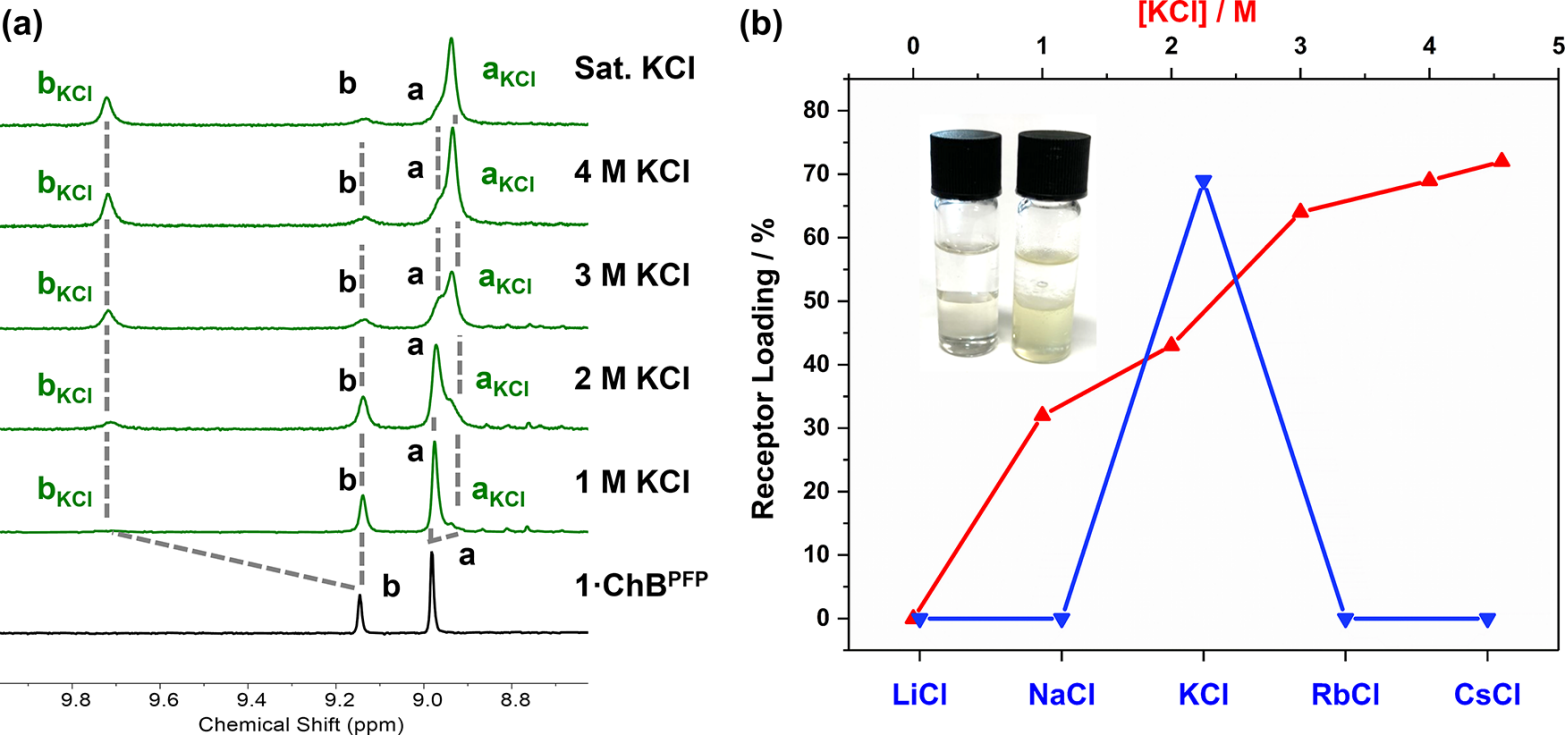
(a) Stacked post-LLE ^1^H NMR spectra of **1·ChB**^**PFP**^ with varying KCl source phase concentrations. (b) Plot showing salt extraction performance for various KCl source phase concentrations (red) and group 1 metal chlorides (blue) under LLE with 4 M MCl aqueous phases; inset: picture of pre- and post-extraction solutions.

The LLE experiments, in which a CDCl_3_ solution of **1·ChB**^**PFP**^ was exposed to a potassium chloride D_2_O solution, exhibited similar changes in the post-extraction ^1^H NMR spectrum, enabling relative LLE extraction percentages to be calculated by a similar method to ^1^H NMR signal integration. The percentage of KCl salt complexed receptor was determined to be 32, 43, 64, 69, and 71% when exposed to 1, 2, 3, 4 M, and saturated aqueous KCl solutions, respectively (Figure 7a). As anticipated, the percentage of LLE extraction increases with increasing KCl source phase concentration. Impressively, under these LLE conditions, KCl selectivity is even more pronounced than in the SLE experiment and is the only group 1 metal chloride to be extracted (Figure 7b). To further confirm the role of ChB–anion interactions in the SLE and LLE processes, analogous alkali metal chloride salt extraction experiments were conducted with **1·ChB**^**Ph**^. Crucially, an inspection of the SLE or LLE post-extraction ^1^H NMR spectra for **1·ChB**^**Ph**^ demonstrates that this receptor is incapable of any measurable group 1 chloride salt extraction, highlighting the requirement for the electron-withdrawing perfluorophenyl substituent and K^+^ sandwich complex formation to facilitate ChB-mediated chloride anion recognition in the overall KCl selective extraction capability of **1·ChB**^**PFP**^.

**Figure 8:**
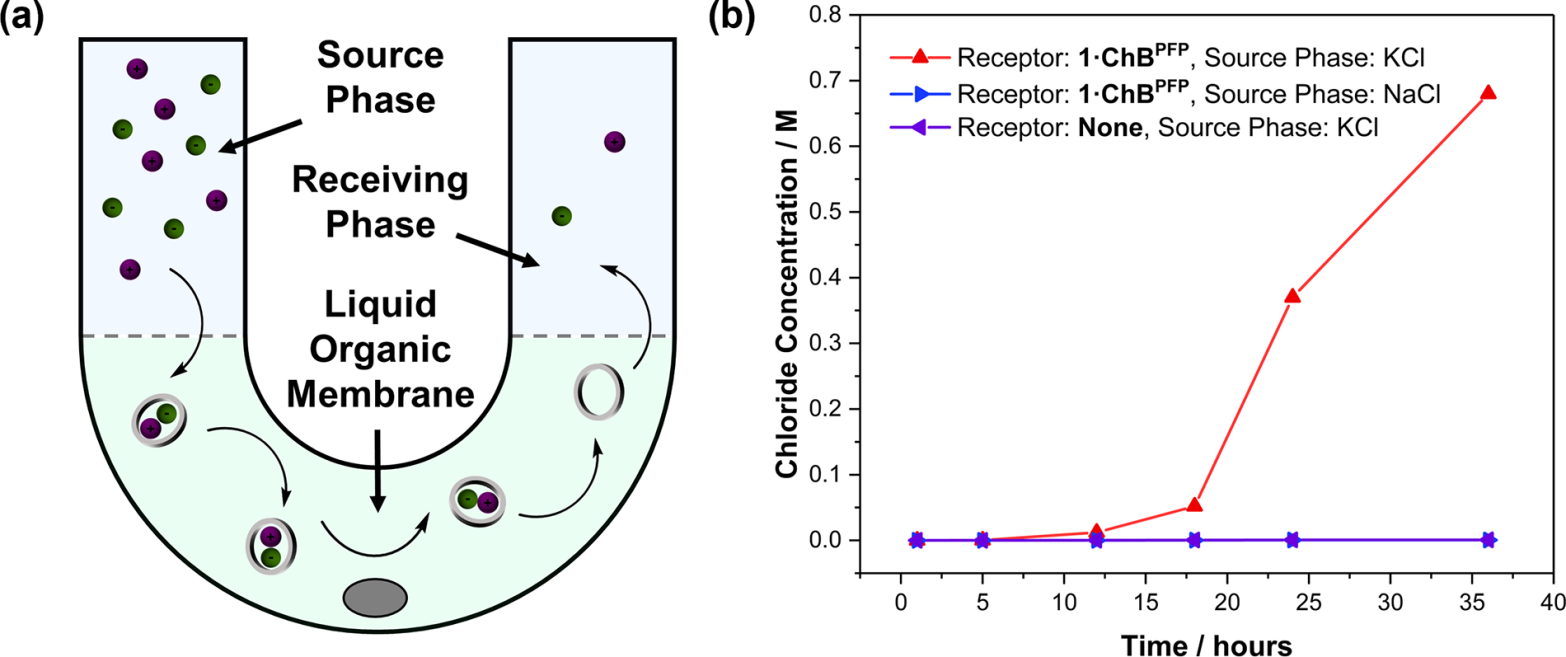
(a) Cartoon representation of the U-tube experiment and (b) calculated changes in chloride receiving phase concentration in the U-tube experiment as determined by potential measurements.

Motivated by the excellent KCl selectivity exhibited by **1·ChB**^**PFP**^, preliminary liquid membrane U-tube transport experiments were performed. Specifically, a 4 M KCl aqueous source phase and a deionized water receiving phase were separated by a stirred 8 mM solution of **1·ChB**^**PFP**^ in CHCl_3_ (Figure 8a). Monitoring the receiving phase chloride concentration via an ion-selective electrode (Figure 8b) revealed a steady increase over the course of 35 h, after which the chloride concentration was determined to be ca. 0.7 M, and co-transport of potassium was confirmed by flame test analysis of the receiving phase. Crucially, the co-dependence of K^+^ and Cl^–^ transport was demonstrated by a NaCl source phase, in which no perturbation in the receiver-phase chloride concentration was observed.

### DFT Computational Studies

Having demonstrated the unique ion-pair recognition properties of **1·ChB**^**PFP**^, DFT calculations were performed at the M06-2X/Def2-TZVP(CPCM) theory level to understand how the electronic and anion binding properties of the ChB receptors are influenced by alkali cation complexation. This extensive theoretical study was performed in the gas phase, chloroform, and acetonitrile, given that the experimental anion recognition studies were performed in a 50/50 v/v mixture of these solvents. The remaining computational details are given in the Supporting Information.

**Figure 9:**
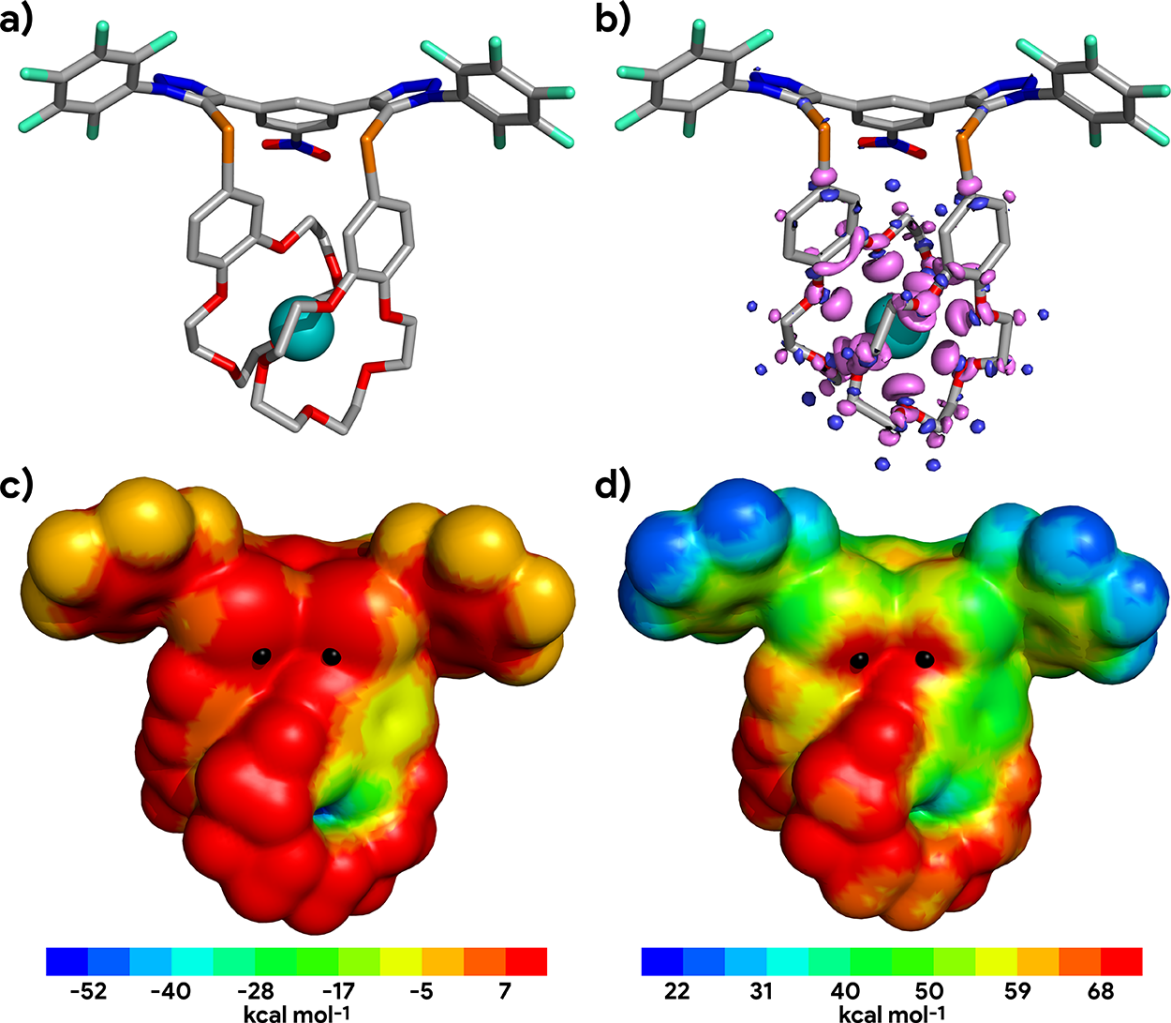
Electronic features of the computed structure of the [K^+^ + **1·ChB**^**PFP**^] cationic receptor: (a) gas-phase DFT optimized structure (H atoms were hidden for clarity) and (b) electron density difference map for the [K^+^ + **1·ChB**^**PFP**^] complex (Δρ = ρ[K^+^ + **1·ChB**^**PFP**^] – ρ[**1·ChB**^**PFP**^] – ρ[K^+^]). Blue indicates an increase of electron density (+0.002 *e*a_0_^–3^ contour), and purple indicates a loss of electron density (−0.002 *e*a_0_^–3^ contour). Distribution of the electrostatic potential mapped on the 0.001 *e*a_0_^–3^ isodensity surface (*V*_S_) of (c) free [**1·ChB**^**PFP**^] and of (d)*V*_S_ of free [K^+^ + **1·ChB**^**PFP**^]. The highest point of *V*_S_ in front of each ChB-binding unit (i.e., the approximate location of its σ-hole) is marked with a black dot.

The structures of K^+^, Rb^+^, and Cs^+^ bis-B15C5 sandwich complexed **1·ChB**^**PFP**^ receptors were optimized in the gas phase and are illustrated in Figure 9a with the [K^+^ + **1·ChB**^**PFP**^] complex. The distances between the alkali metal cations and crown-ether oxygen atoms are listed in Table S14. Overall, the distances to the aryl oxygen atoms are typically longer than those to the aliphatic ones and naturally increase with the size of the encapsulated cation. Furthermore, in agreement with natural bond orbital (NBO) analysis, the M···O bonds result from the interaction of the electron lone pairs of the oxygen atoms with the alkali metal's lone vacant orbitals (*n*_O_ → LV_M_). These ten bonding contacts lead to the second-order perturbation theory stabilization energies (*E*^2^) also given in Table S14, which amount to 36.1, 35.1, and 26.7 kcal mol^–1^, reflecting the average M···O distances of the respective K^+^, Rb^+^, and Cs^+^ complexes. The complexation of each alkali cation is accompanied by charge transfer from the oxygen atoms to the sandwiched metal center, as indicated by the natural population analysis (NPA) charges of K^+^ (0.859 *e*), Rb^+^ (0.869 *e*), and Cs^+^ (0.884 *e*). Concomitantly, a charge redistribution within the entire alkali sandwich **1·ChB**^**PFP**^ receptor occurs, with the NPA charges centered on the tellurium anion binding sites increasing on average from ca. 0.57 to 0.60 *e* upon cation binding, as detailed in Table S15. In other words, the alkali metal cation polarizes the receptor’s electron density. This electron density variance is also observed in the crown-ether aryl rings, including the carbon atoms connected to the Te centers, as illustrated in Figure 9b for the [K^+^ + **1·ChB**^**PFP**^] cationic complex, where the electron density difference between the complex and free **1·ChB**^**PFP**^, in the complex conformation, is plotted.

By design, the highest points of the electrostatic potential on the electron density surface (*V*_S,max_) of the neutral **1·ChB**^**PFP**^ receptor are in front of its ChB-binding units, inherently activated by the perfluorinated aryl electron-withdrawing groups, as assessed on the receptor’s cation organized conformation, with *V*_S,max_ values of ca. 32 kcal mol^–1^, while the lowest value of *V*_S_ (*V*_S,min_) is located between the crown-ethers, consistent with cation complexation. The *V*_S_ of free **1·ChB**^**PFP**^ is illustrated in Figure 9c, together with the *V*_S_ of the [K^+^ + **1·ChB**^**PFP**^] sandwich complex (Figure 9d). Overall, the encapsulation of either alkali cation leads to an increase in the value of *V*_S_, with the *V*_S,max_ still located in front of the ChB-binding units in the K^+^ and Rb^+^ complexes, having values of ca. 80 kcal mol^–1^. In the Cs^+^ complex, the imperfect fitting of the bis-crown moiety and the larger cation leads to a *V*_S,max_ point of 89.8 kcal mol^–1^ near the partially exposed cation, followed by two *V*_S_ points in front of the ChB-binding units, with slightly lower average values of 88.2 kcal mol^–1^.

**Figure 10:**
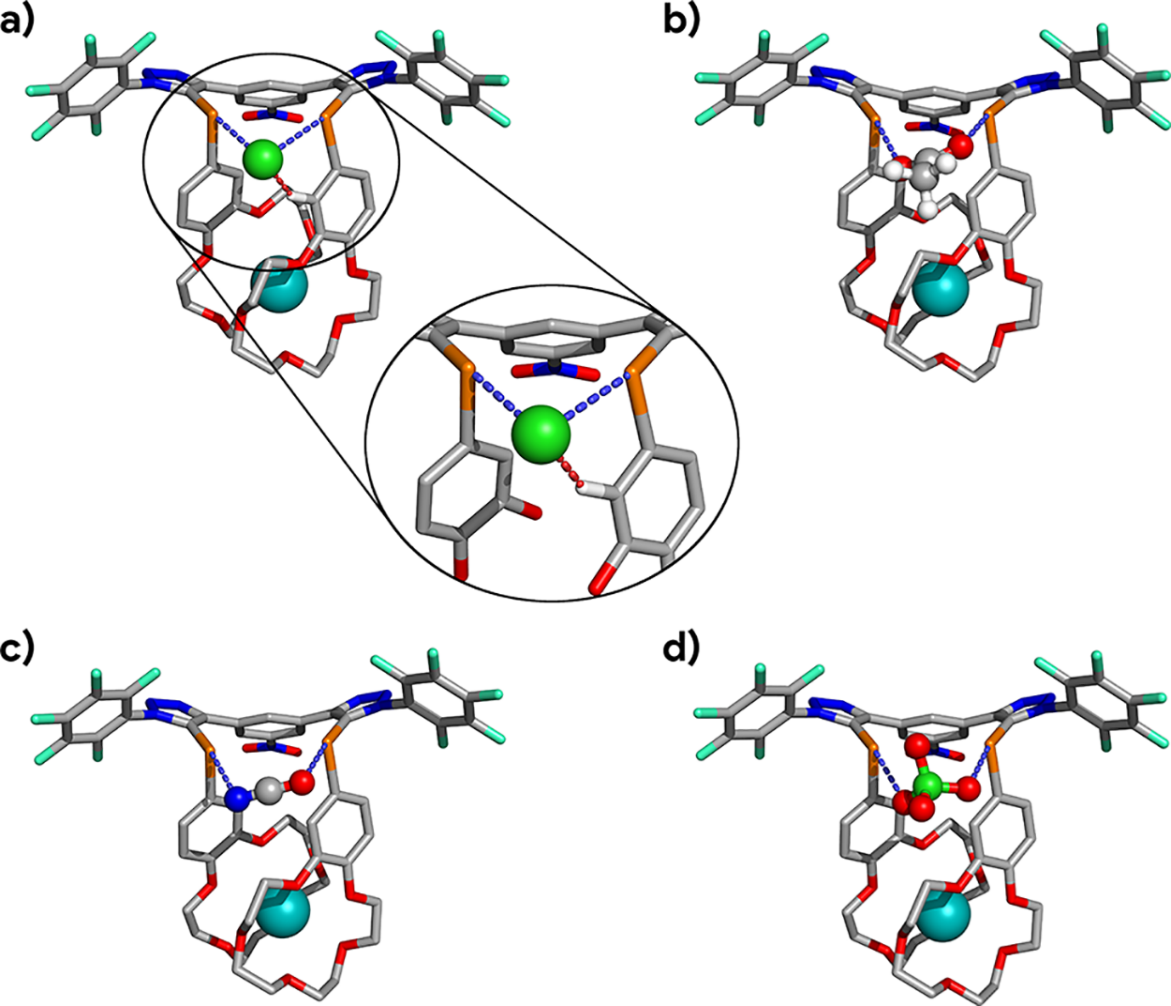
M06-2X/Def2-TZVP optimized structures of **1·ChB**^**PFP**^ complexes with (a) KCl, (b) KOAc, (c) KOCN, and (d) KClO_4_ ion pairs in chloroform. The ChB interactions are drawn with light blue dashes. The computed HB interaction in the KCl ion-pair complex is drawn with red dashes and is highlighted as an inset of (a). Apart from the donor C_Ar_–H proton, all hydrogen atoms are hidden for clarity. Grey = carbon, blue = nitrogen, red = oxygen, light green = fluorine, orange = tellurium, cyan = potassium, green = chlorine, white = hydrogen.

The crystal structures of the ion-pair complexes of **1·ChB**^**PFP**^, apart from the CsI complex, (vide supra) present chloroform solvent molecules establishing C–H···X^–^ short bonding contacts (HB) with the halides, putatively affecting the ChB dimensions. This fact inspired us to start a theoretical investigation on the **1·ChB**^**PFP**^ complexes of the KCl, KBr, KI, RbI, and CsI ion pairs with geometry optimizations in chloroform. The distances and angles for HB and ChB interactions are gathered in Table S16. All computed structures display two almost linear ChB interactions with markedly different Te···X^–^ distances, as found in the corresponding crystal structures. As illustrated in Figure 10a for chloride chalcogen bonded by [K^+^ + **1·ChB**^**PFP**^] and in Figure S57 for the remaining halide ion-pair complexes, the longer interaction results from the existence of a single ancillary C_ar_–H···X^–^ bond that pushes the anion away from this chalcogen binding unit, weakening the adjacent ChB bond. The computed ChB dimensions naturally increase with the size of the guest halide and compare well with those observed in the solid state (Table S17). On the other hand, as observed in the crystal structures of **1·ChB**^**PFP**^ with KI, RbI, and CsI, the dimensions of the ChB and HB interactions appear to be independent of the sandwiched alkali cation, which should be expected as the anion and cation are separated by a long distance (at least 7 Å, Table S17). Within the cation sandwich moiety of the **1·ChB**^**PFP**^ receptors, the distances to the aromatic oxygens are longer than those to the aliphatic ones. Furthermore, as observed in the gas-phase optimized structures of the cationic sandwich complexes of **1·ChB**^**PFP**^, the M···O distances increase following the cation size (K^+^ < Rb^+^ < Cs^+^), with the crystallographic and computed values being similar (Table S17).

To rationalize the experimental binding data gathered in Table 1, our DFT calculations continued with geometry optimizations of [K^+^ + **1·ChB**^**PFP**^] halide complexes in acetonitrile, being also extended to oxoanion ion pairs in both solvents. In the absence of the crystal structures of **1·ChB**^**PFP**^ with these anion guests, their initial binding arrangements were conceived by positioning an oxygen atom (and nitrogen atom in OCN^–^) in front of each C_trz_–Te bond (C_trz_ is the triazole’s carbon atom), regardless of the anion shape, establishing two putative chalcogen bonds. These interactions are maintained in the optimized structures, as illustrated in Figure 10b–d for the complexes of **1·ChB**^**PFP**^ with KAcO, KOCN, and KClO_4_ and in Figure S57 for the KNO_3_ and KNO_2_ complexes. The ChB and HB dimensions together with the alkali···O distances are given in Table S18 for both solvents. In contrast with the halide complexes of [K^+^ + **1·ChB**^**PFP**^], the oxoanions are recognized by two ChB interactions with comparable distances, ranging from 2.644 or 2.678 Å for basic AcO^–^ to 2.943 or 3.015 Å for the less basic tetrahedral ClO_4_^–^, in chloroform or acetonitrile implicit solvents. HB interactions between **1·ChB**^**PFP**^ and the oxoanions are also discernible but of smaller importance in the overall anion recognition process, as discussed in the Supporting Information. The binding free energies between [K^+^ + **1·ChB**^**PFP**^] and the chalcogen bonded anions were calculated for both solvents, as thoroughly reported in the Supporting Information. The inclusion of an additional diffusion function in the anions’ and Te centers’ basis sets (Def2-TZVP(D)), to describe the σ-hole-based interactions more accurately, was also investigated.^78^ The binding free energies are listed in Tables S19 (halides) and S20 (oxoanions) together with their enthalpic (Δ*H*) and entropic (TΔ*S*) energy terms estimated at 298.15 K, as well as the standard-state-corrected binding free energies (Δ*G*_SS_). Whereas a comparison between basis sets shows that the dimensions of the ChB interactions are negligibly affected (see Tables S16 and S18), better fittings between the association constants and the Δ*G*_SS_ values computed with the augmented basis set were observed (Figure S58), leading us to focus on the subsequent discussion on the Def2-TZVP(D)’s results. The energy penalty inherent to anion recognition appears to be independent of the solvent, depending only on the anion type with the [K^+^ + **1·ChB**^**PFP**^] complexes with oxoanions having higher −*T*Δ*S* values. On the other hand, the recognition of each anion in chloroform is more exothermic than that in acetonitrile due to the weaker interactions between the cationic sandwich complexes and the anionic guests in a more polar solvent, as suggested by the slightly longer ChB and HB distances. In agreement, when the Δ*G*_SS_ and Δ*H* values are grouped by halide and oxoanion series, they follow linear relationships (Figure S59).

The computed binding free energies for the halide series, following the sequence Cl^–^ > Br^–^ > I^–^, mirror the experimental binding affinity order (Table 1), in agreement with the gas-phase *V*_S,min_ values estimated for halides (Cl^–^ (−140.2) < Br^–^ (−132.3) < I^–^ (−123.1), in kcal mol^–1^). The Δ*G*_SS_ values computed for the oxoanions also correlate with the experimental binding trend (AcO^–^ > OCN^–^ > NO_2_^–^ > NO_3_^–^), regardless of the anion geometry and gas-phase *V*_S,min_ (AcO^–^ (−154.9) < NO_2_^–^ (−150.4) < OCN^–^ (−140.7) ≈ NO_3_^–^ (−140.3), in kcal mol^–1^). The estimated binding affinity of [K^+^ + **1·ChB**^**PFP**^] toward ClO_4_^–^ (*V*_S,min_ = −124.4 kcal mol^–1^) is low in chloroform and almost nonexistent in acetonitrile (Table S20), being in line with the experimental absence of binding in the solvent mixture (Table 1). Moreover, when compared with Cl^–^, the interaction of [K^+^ + **1·ChB**^**PFP**^] with ClO_4_^–^ is disfavored in both solvents (ca. 7 kcal mol^–1^ in chloroform and ca. 5 kcal mol^–1^ in acetonitrile), rationalizing its binding selectivity for the halide in the presence of this oxoanion (Table 2). The strength of ChB interactions was further ascertained by computing the *E*^2^ interaction energies between the anions’ lone pairs and the antibonding orbitals of the C_trz_–Te and ancillary C_Ar_–H binding units. Noteworthy, as discussed in the Supporting Information, for each solvent, the *E*^2^ values for the ChB interactions linearly correlate with the computed Δ*H* binding values when the OCN^–^ complex is excluded from the data enclosing halide and oxoanion complexes. Overall, the selectivity of [K^+^ + **1·ChB**^**PFP**^] for the anions is mainly dictated by the directional ChB interactions.

## Conclusions

Heteroditopic receptors exhibiting pronounced selectivity for potassium over sodium salts are rare, and those selective for KCl are unknown. Herein, a novel ChB heteroditopic receptor is developed and demonstrated to be capable of selective KCl recognition over all other alkali metal chlorides. Importantly, the origin of this selectivity behavior hinges upon intramolecular bis-benzo-15-crown-5 ether metal cation sandwich complex-induced electronic polarization of conjugated proximal Te centers switching on electrophilic ChB donor potency. Significantly, the extent of this polarization is strongly dependent on the charge density of the bound alkali metal cation, thereby generating a powerful ion-pair cooperativity mechanism. This unique cooperativity mechanism underpins the ability of **1·ChB**^**PFP**^ to perform selective extraction of KCl under solid–liquid and liquid–liquid extraction conditions. Furthermore, preliminary liquid membrane U-tube transport experiments reveal the ChB heteroditopic receptor’s ability to exhibit selective KCl transmembrane transport over NaCl, which demonstrates remarkable promise as a novel treatment strategy for channelopathy-related conditions and cancer cell proliferation. These results, corroborated by computational DFT methods, serve to highlight that judicious control of the stereoelectronic factors that govern ChB-mediated recognition is a powerful strategy in engineering potency and selectivity in σ-hole-based anion recognition.

## Supporting Information Available

Additional experimental and computational details; materials, and methods, including photographs of the experimental setup, and DFT data (PDF)
