# Supplementary material for: Selective Potassium Chloride Recognition, Sensing, Extraction, and Transport Using a Chalcogen-Bonding Heteroditopic Receptor
Source: J Am Chem Soc. 2022 Aug 5;144(32):14778–89. doi: 10.1021/jacs.2c05333 (PMC9394446; doi:10.1021/jacs.2c05333)
Supplement: Supplementary file 1 — ja2c05333_si_001.pdf [file ja2c05333_si_001.pdf]

Supporting Information for: Selective Potassium Chloride Recognition, Sensing, Extraction and Transport Using a Chalcogen Bonding Heteroditopic Receptor

Andrew Docker,[a] Igor Marques,[b] Zongyao Zhang,[a] Heike Kuhn,[a] Vítor Félix[b] and Paul D. Beer[a] *

[a] *Chemistry Research Laboratory, Department of Chemistry, University of Oxford, Mansfield Road, Oxford OX1 3TA, U. K.*

[b] *CICECO – Aveiro Institute of Materials, Department of Chemistry, University of Aveiro, 3810-193 Aveiro, Portugal*

Email: paul.beer@chem.ox.ac.uk

Contents

Synthesis and Characterisation 3

General Remarks 3

Synthetic Procedures and Spectra 4

NMR Spectroscopy Experiments 17

^{1}H NMR Cation or Anion Binding Studies 18

^{1}H NMR Ion-Pair Binding Studies 21

Solid-Liquid Extraction (SLE) Procedure 36

Liquid-Liquid Extraction (LLE) Procedure 44

Liquid Membrane Transport Studies 51

Computational Studies 54

Detailed Methods 54

Detailed Discussion 54

Supplementary Figures 55

Supplementary Tables 59

References for Computational Studies 66

Crystal Structure Determination 67

Synthesis and Characterisation

General Remarks

All solvents and reagents were purchased from commercial suppliers and used as received unless otherwise stated. Dry solvents were obtained by purging with nitrogen and then passing through an MBraun MPSP-800 column. H_2O was de-ionized and micro filtered using a Milli-Q ® Millipore machine. Column chromatography was carried out on Merck® silica gel 60 under a positive pressure of nitrogen. Routine NMR spectra were recorded on either a Varian Mercury 300, a Bruker AVIII 400 or a Bruker AVIII 500 spectrometer with ^{1}H NMR titrations recorded on a Bruker AVIII 500 spectrometer. TBA salts were stored in a vacuum desiccator containing phosphorus pentoxide prior to use. Where mixtures of solvents were used, ratios are reported by volume. Chemical shifts are quoted in parts per million relative to the residual solvent peak. Mass spectra were recorded on a Bruker μTOF spectrometer. Triethylamine was distilled from and stored over potassium hydroxide. Tris[(1-benzyl-1H-1,2,3-triazol-4-yl)methyl]amine (TBTA).

Synthetic Procedures and Spectra

1

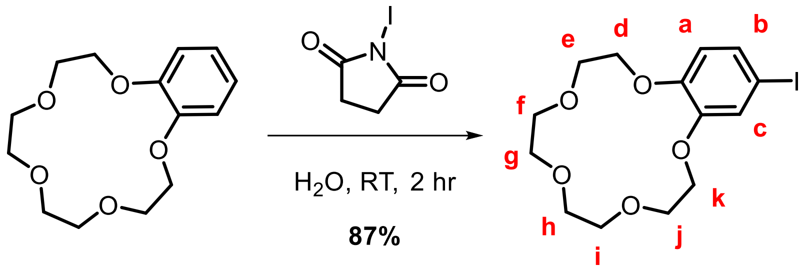

Benzo-15-crown-5 (5.00 g, 18.6 mmol) was dissolved in H_2O (200 ml) to which was added NIS (4.39 mmol, 19.5 mmol) portion wise. The mixture was stirred at room temperature, under the exclusion of light, for 2 hours after which time a brown precipitate formed. The reaction mixture was diluted with H_2O (200 ml) and extracted with CH_2Cl_2 (300 ml). The organic phase was collected and washed with H_2O (3 × 200 ml), dried over $MgSO_4$ and the solvent removed *in vacuo* and isolated as an off white solid (6.38g, 16.2 mmol, 87%).

^{1}H NMR (400 MHz, $CDCl_3$) δ 7.20 (dd, *J* = 8.4, 2.0 Hz, $1H_a$), 7.13 (d, *J* = 2.0 Hz, $1H_b$), 6.61 (d, *J* = 8.4 Hz, $1H_c$), 4.16 – 4.05 (m, $4H_{d,k}$), 3.97 – 3.85 (m, $4H_{e,k}$), 3.81 – 3.69 (s, $8H_{f-j}$).

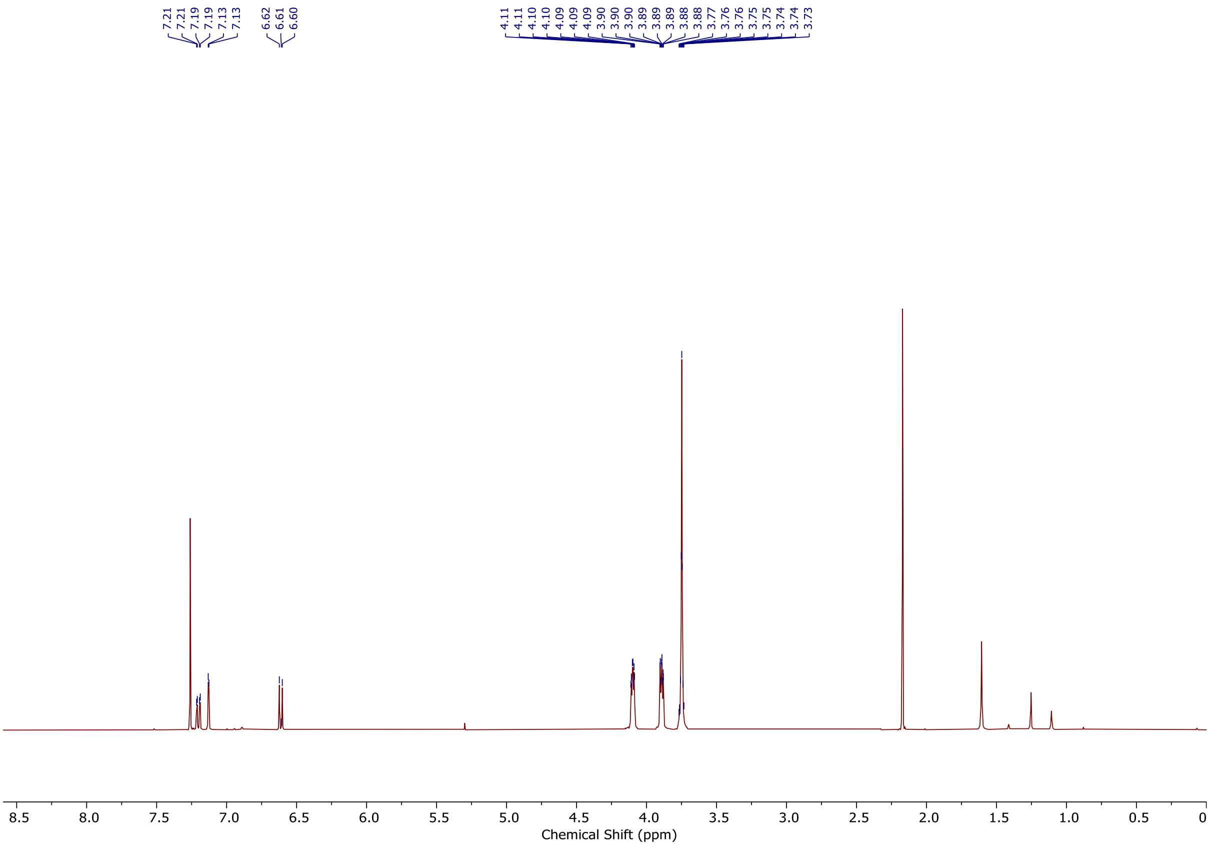

Figure S1. ^{1}H NMR spectrum of **1** ($CDCl_3$, 500 MHz, 298K).

2

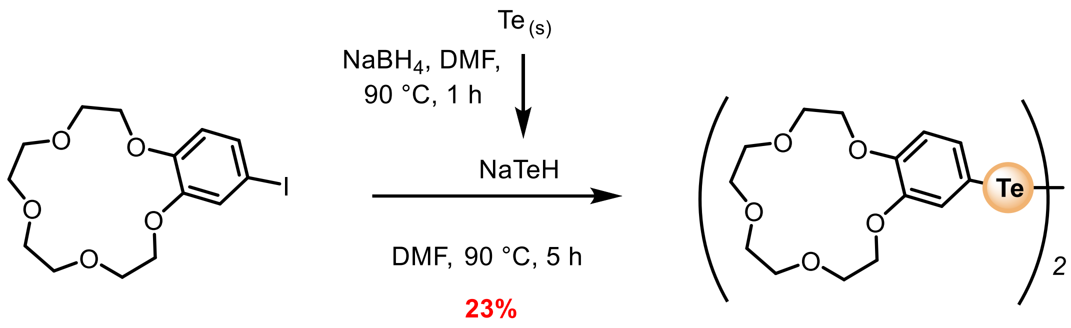

Elemental tellurium (1.136 g, 8.88 mmol) and $NaBH_4$ (369 mg, 9.77 mmol) were suspended in anhydrous degassed DMF (15 ml) and heated to 90 °C for 30 minutes under an atmosphere of N_2. After which time an intense purple suspension formed, this mixture was subsequently cooled to 0 °C and **1** (1.75 g, 4.44 mmol) in DMF (3 ml) was added dropwise. The resultant mixture was heated to 90 °C and left to stir for 5 hours. After which time the reaction mixture was again cooled to 0 °C and H_2O (50 ml) added dropwise. The mixture was vigorously stirred in air for 1 hour and diluted with CH_2Cl_2 (250 ml), the organic phase was collected and the aqueous phase extracted with CH_2Cl_2 (5 x 250 ml), the combined organics were subsequently washed with H_2O (3 x 250 ml), dried over $MgSO_4$ and solvent removed in vacuo. The crude black tar was subjected to silica gel column chromatography eluting with a gradient of CH_2Cl_2 $\rightarrow$ CH_2Cl_2;MeOH (97:3, v/v) which gave **2** as an orange solid (403 mg, 0.511 mmol).

A yield of 23 % is estimated for the synthesis of **2**, due to inseparable impurities which were determined to be the corresponding telluride.

^{1}H NMR (400 MHz, $CDCl_3$) δ 7.30 (dd, J = 8.1, 1.8 Hz, 2H), 7.26 (s, 2H), 6.66 (d, J = 8.1 Hz, 2H), 4.15 – 4.04 (m, 8H), 3.98 – 3.84 (m, 8H), 3.75 (d, J = 1.8 Hz, 16H).

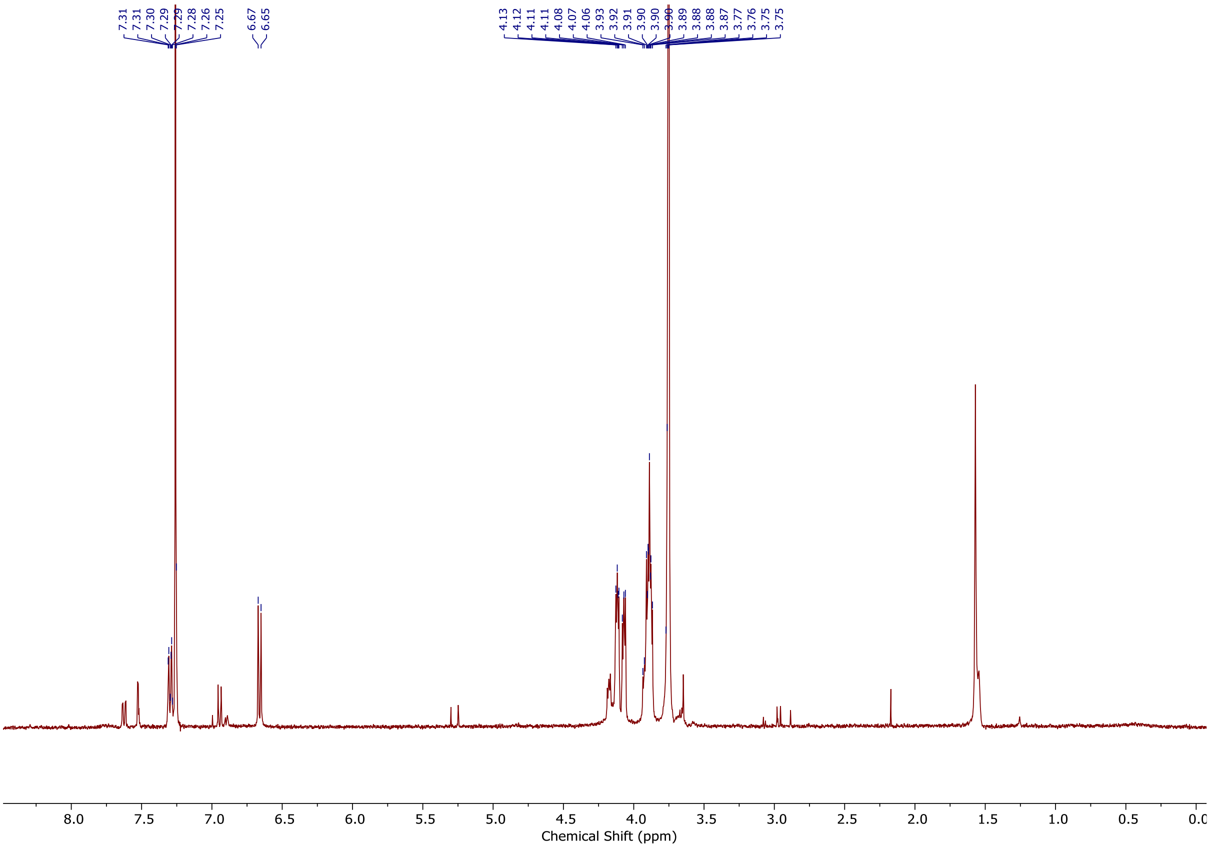

Figure S2. ^{1}H NMR spectrum of **2** ($CDCl_3$, 500 MHz, 298K).

General Procedure 1

Ditelluride **2** (1 eq.) was dissolved in THF (5 ml) and cooled to 0°C, to which was added a 1 M Br_2 solution in CH_2Cl_2 (1 eq.) dropwise, after which time the ditelluride solution darkened indicating the formation of (B15C5)TeBr which was added immediately to a THF suspension of silver acetylide **3** (0.5 eq.). The reaction mixture was left to stir for 30 minutes at room temperature protected from light, after which time it was filtered through celite and the reaction mixture was concentrated to dryness in vacuo. The crude mixture was redissolved in CH_2Cl_2 and washed with 0.1 M NH_4OH/EDTA dried over $MgSO_4$ and concentrated to dryness in vacuo to afford the crude telluroalkyne.

The crude telluroalkyne was used immediately for the following CuAAC reaction. $[Cu(MeCN)_4]PF_6$ (0.2 equivalents) and TBTA (0.2 equivalents) were dissolved in the minimum amount of anhydrous degassed CH_2Cl_2 (ca. 5 ml) and left to stir for 15 minutes. After which time, the crude telluroalkyne was added to the solution as a CH_2Cl_2 solution, followed by the appropriate phenyl azide (2.2 equivalents). Once complete, as determined by TLC analysis, the reaction mixture was diluted with CH_2Cl_2 (ca. 150 ml) and washed with EDTA/$NH_4OH_{(aq)}$ solution (20 ml) and water (50 ml), the collected organic phase was dried over $MgSO_4$ and concentrated to dryness and purified by silica gel column chromatography (eluting with a gradient of CH_2Cl_2 → CH_2Cl_2;MeOH (98:2, v/v) to afford the target products as light yellow solids.

1·ChB^PFP^

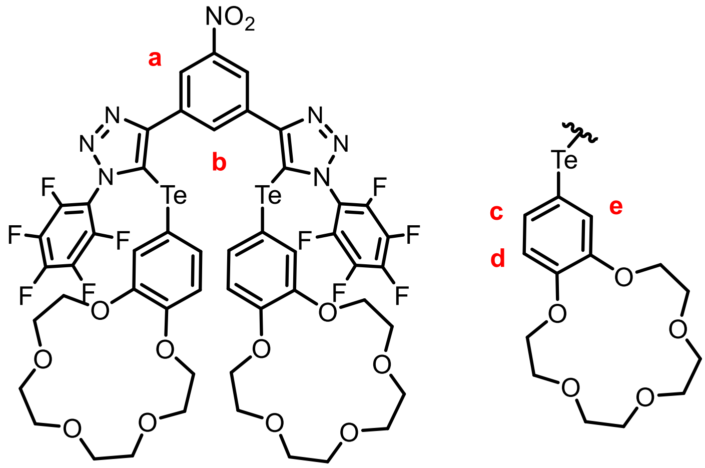

General Procedure 1: 72%

^{1}H NMR (500 MHz, $CDCl_3$) δ 9.15 (t, *J* = 1.7 Hz, $1H_b$), 8.98 (d, *J* = 1.7 Hz, 2H), 7.05 (dd, *J* = 8.2, 1.8 Hz, $2H_c$), 7.00 (d, *J* = 1.9 Hz, $2H_e$), 6.60 (d, *J* = 8.2 Hz, $2H_d$), 4.05 (t, *J* = 4.3 Hz, 4H), 3.95 (t, *J* = 4.3 Hz, 4H), 3.87 (t, *J* = 4.3 Hz, 4H), 3.82 (t, *J* = 4.3 Hz, 4H), 3.71 (m, 16H).

^{13}C NMR (126 MHz, $CDCl_3$) δ 151.25, 151.08, 150.09, 148.65, , 143.81 (dm, *J* = 252 Hz), 143.31 (dm, *J* = 258Hz), 138.01 (dm, *J* = 253Hz), 133.25, 133.18, 132.67, 124.48, 122.85, 114.72, 113.56 (m), 107.65, 100.39, 71.25, 70.56, 70.48, 69.42, 69.26, 68.87.

^{19}F NMR (470 MHz, $CDCl_3$) δ -141.84 – -142.89 (m), -148.06 (t, *J* = 21.5 Hz), -159.63 (dd, *J* = 21.7, 17.3 Hz).

^{125}Te NMR (126 MHz, $CDCl_3$) δ 498.60 (t, *J* = 28.7 Hz).

HRMS (ESI+ve) m/z: 1380.0784 ($[M+H]^+$, $C_{50}H_{42}F_{10}N_7O_{12}Te$ requires 1380.0833).

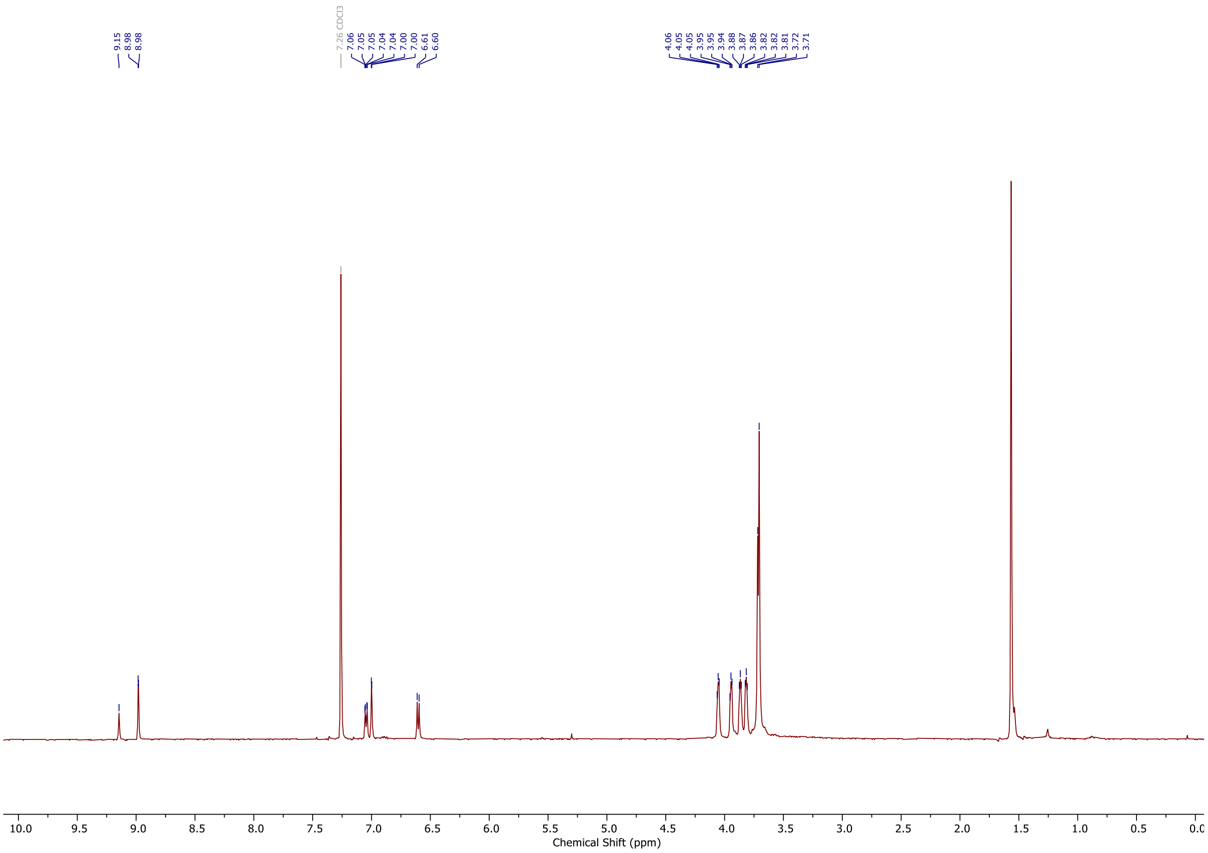

Figure S3. ^{1}H NMR spectrum of **1·ChB^{PFP}** ($CDCl_3$, 500 MHz, 298K).

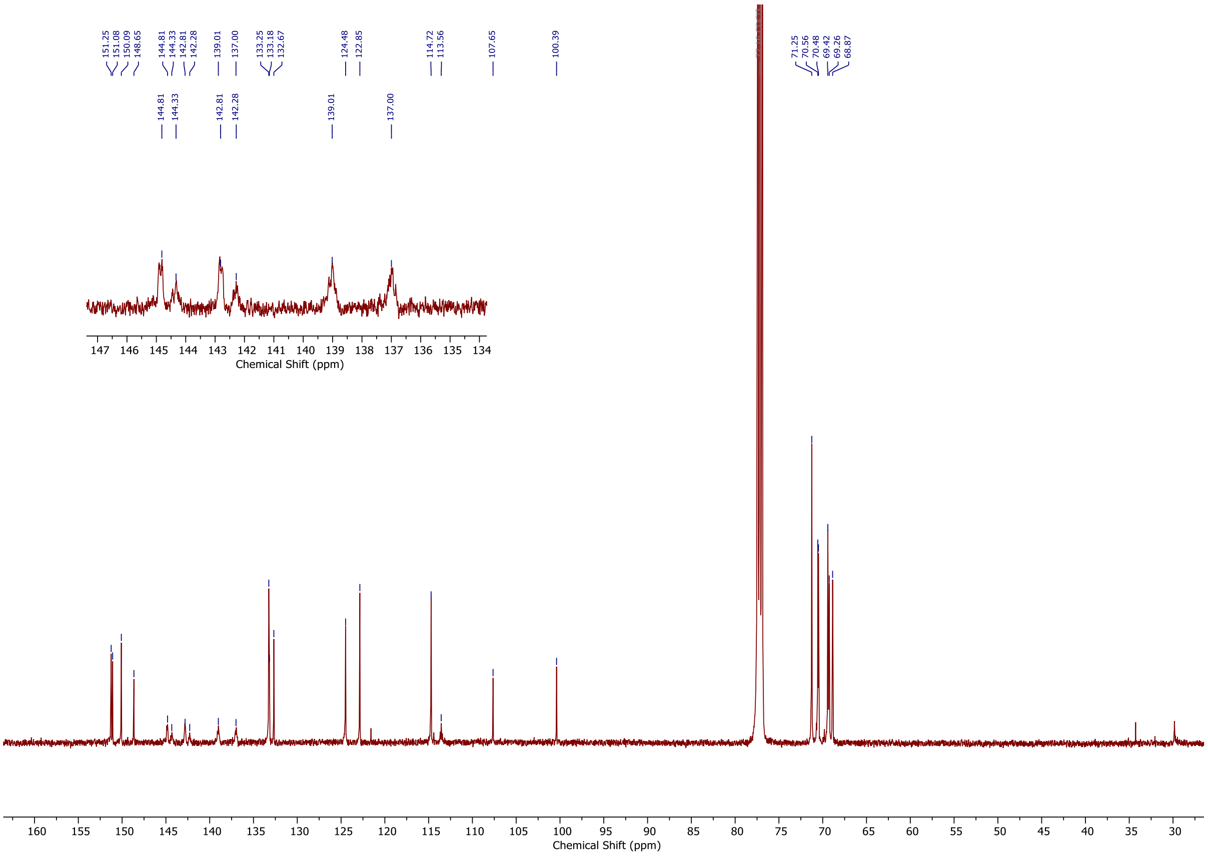

Figure S4. ^{13}C NMR spectrum of **1·ChB^{PFP}** ($CDCl_3$, 126 MHz, 298K).

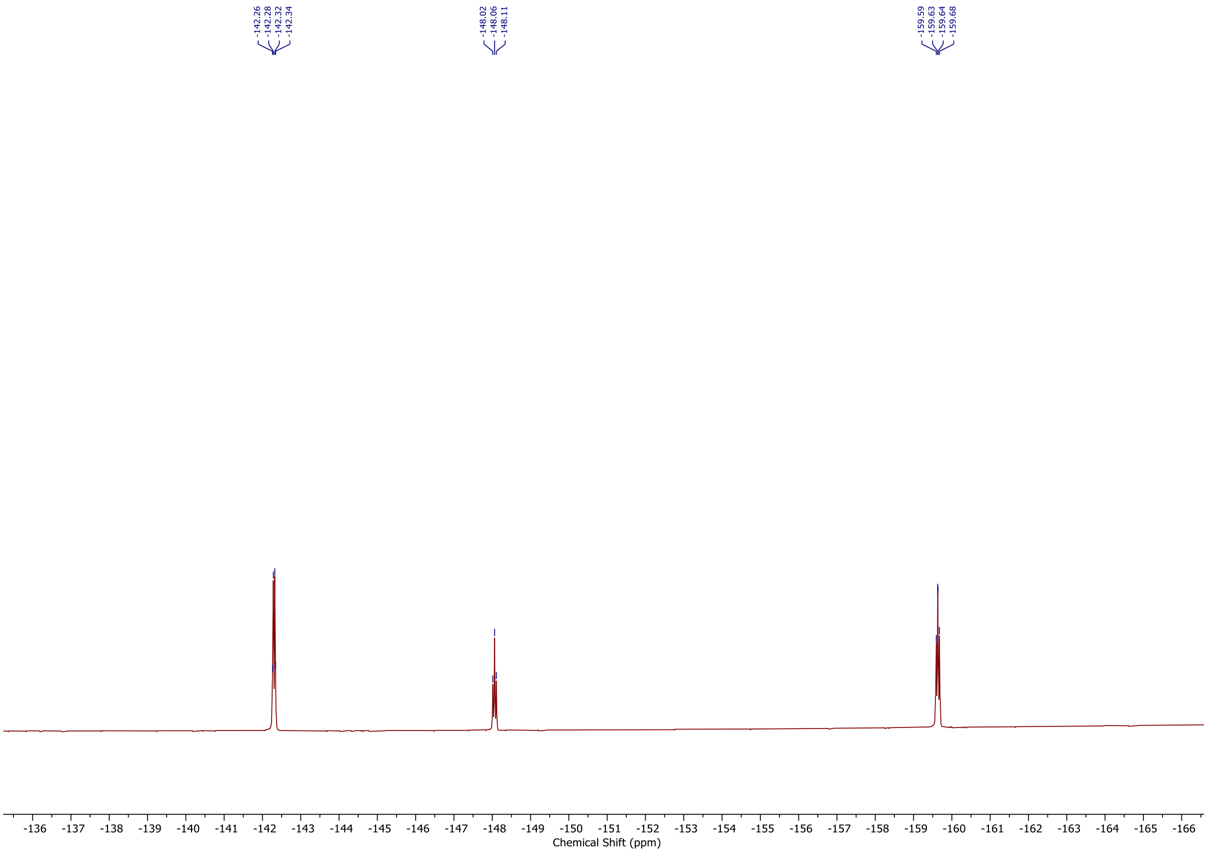

Figure S5. ^{19}F NMR spectrum of **1•ChB^{PFP}** ($CDCl_3$, 470 MHz, 298K).

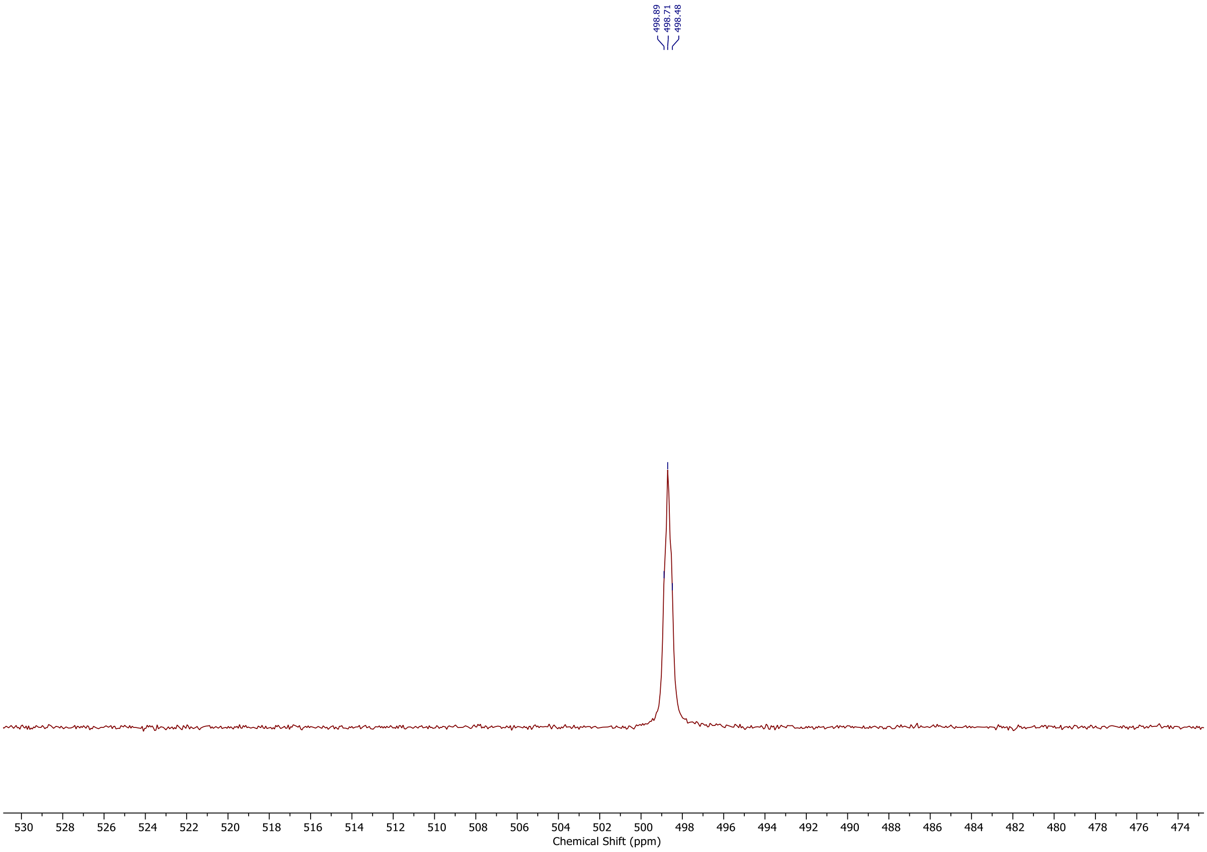

Figure S6. ^{125}Te NMR spectrum of **1•ChB^{PFP}** ($CDCl_3$, 126 MHz, 298K).

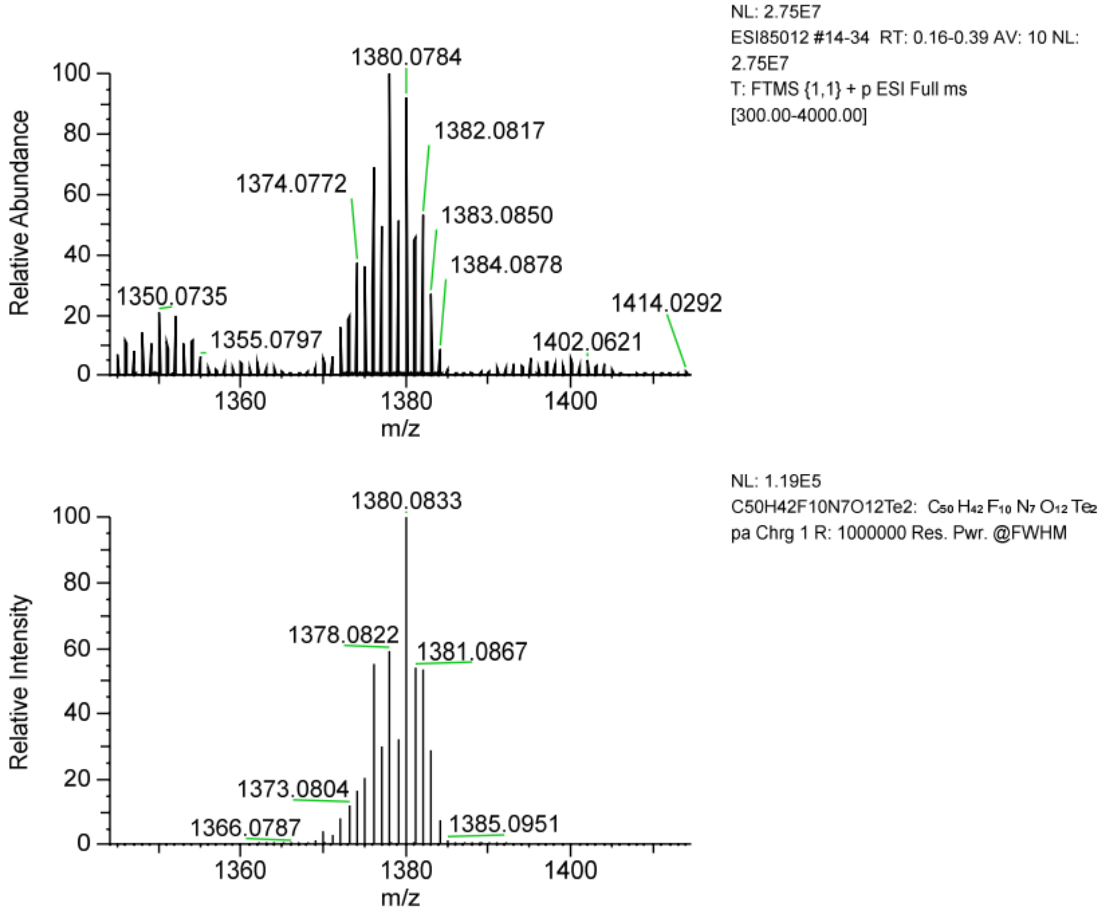

Figure S7. HRESI spectrum of **1·ChB**^{PFP}.

1·ChB^{Ph}

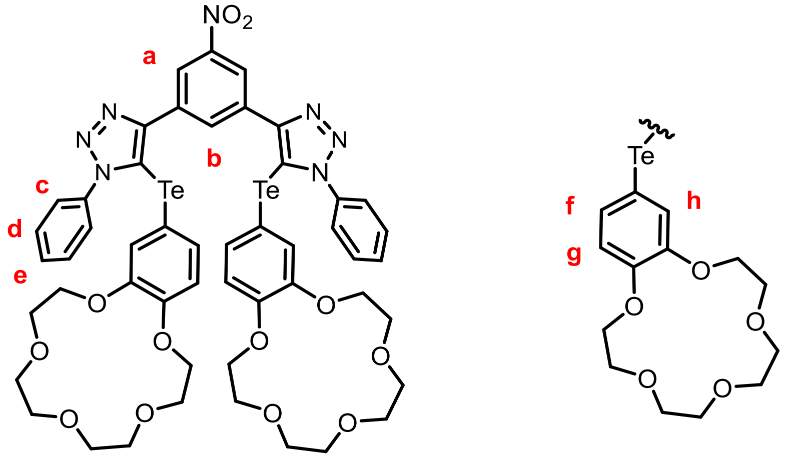

General Procedure 1: 68%

^{1}H NMR (500 MHz, $CDCl_3$) δ 8.98 (s, 1H_b), 8.87 (s, 2H_a), 7.61 – 7.51 (m, 6$H_{c,e}$), 7.51 – 7.40 (m, 4H_a), 6.94 (d, *J* = 8.2 Hz, 2H_f), 6.87 – 6.81 (m, 2H_h), 6.53 (d, *J* = 8.2 Hz, 2H_g), 4.02 (m, 4H), 3.93 – 3.76 (m, 12H), 3.70 (m, 16H).

^{13}C NMR (126 MHz, $CDCl_3$) δ 151.57, 150.53, 149.68, 148.30, 137.90, 134.08, 133.30, 132.96, 130.16, 129.33, 126.88, 124.15, 122.91, 114.82, 105.46, 102.30, 71.19, 70.53, 70.50, 69.43, 69.11, 68.82.

HRMS (ESI+ve) m/z: 1198.1764 ($[M+H]^+$, $C_{50}H_{52}N_7O_{12}Te$ requires 1198.1764).

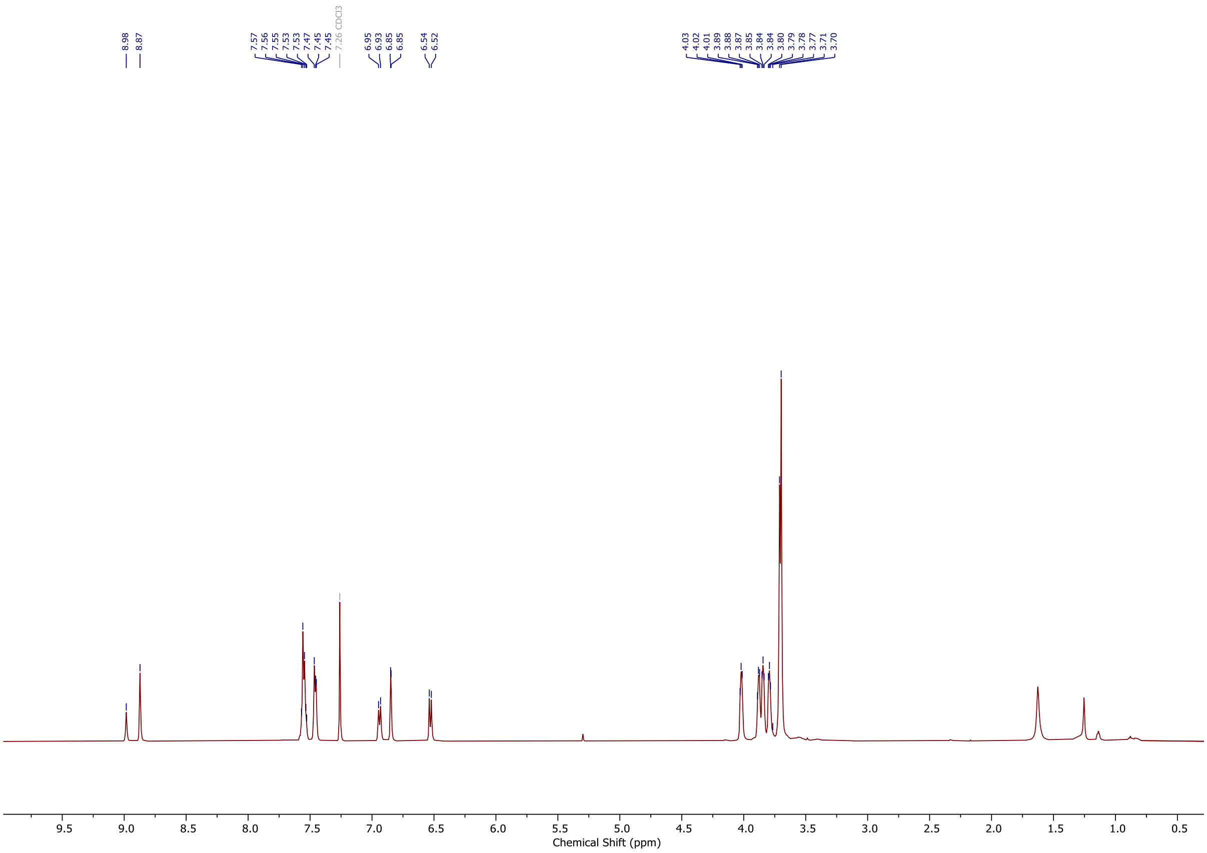

Figure S8. ¹H NMR spectrum of **1•ChB^Ph** ($CDCl_3$, 500 MHz, 298K).

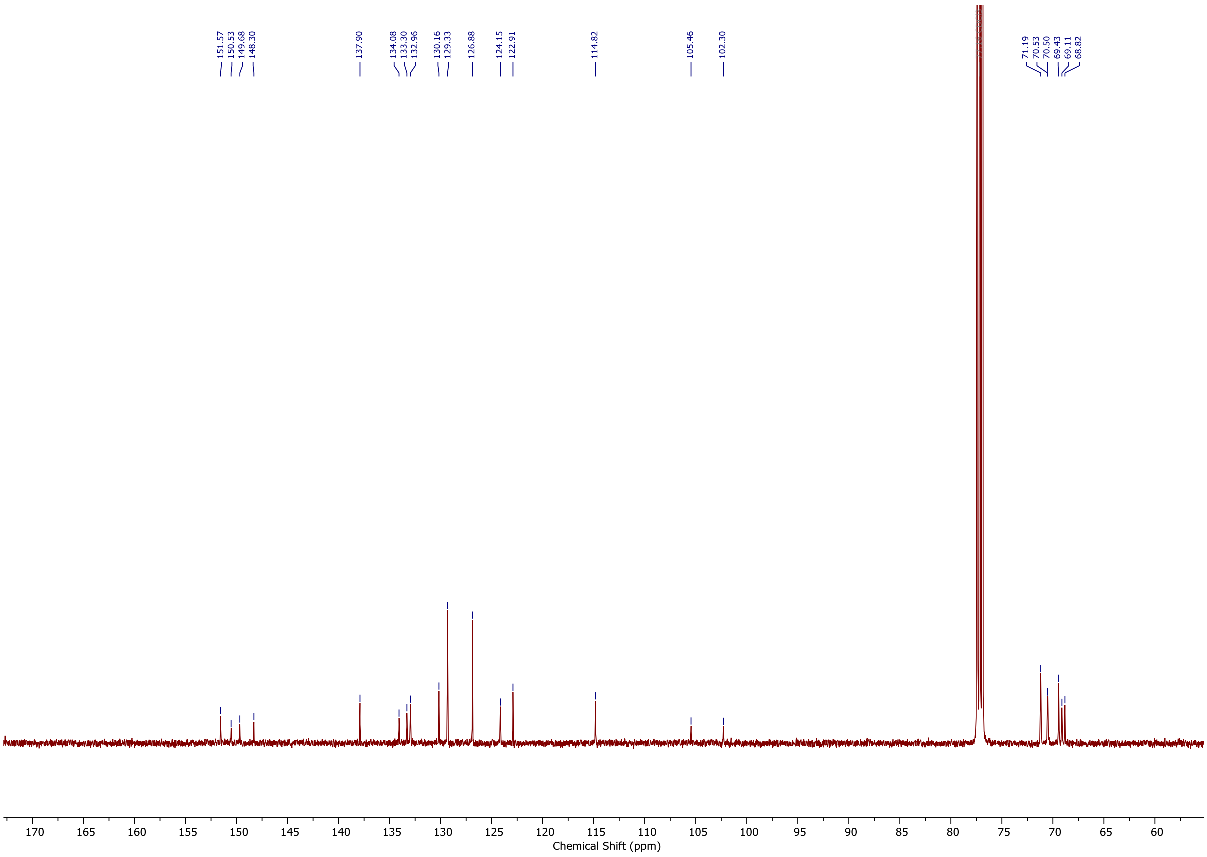

Figure S9. ¹³C NMR spectrum of **1•ChB^Ph** ($CDCl_3$, 126 MHz, 298K).

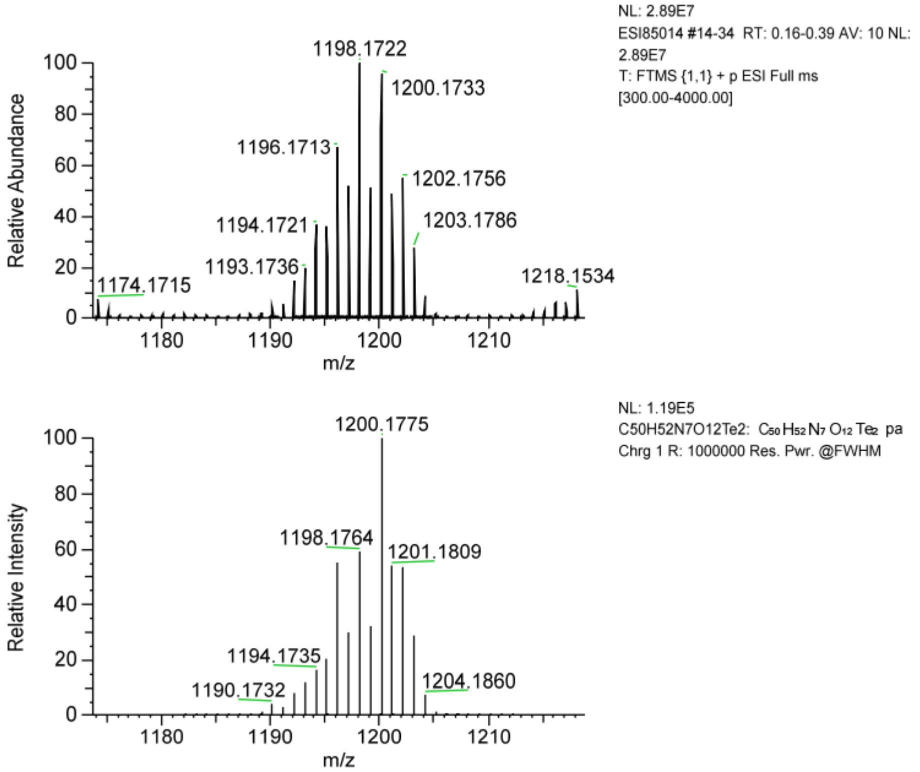

Figure S10. HRESI spectrum of **1·ChB**^{Ph}.

S1

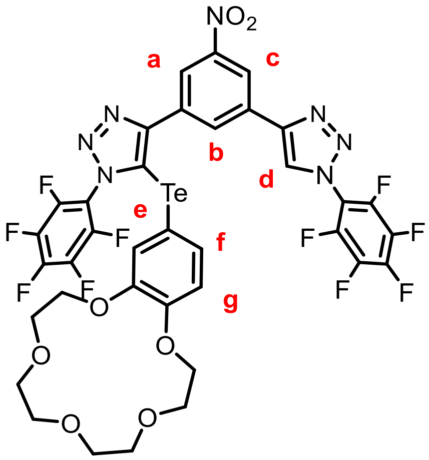

Isolated as a side product from General Procedure 1: 5%

^{1}H NMR (500 MHz, $CDCl_3$) δ 8.92 (m, $2H_{a,c}$), 8.78 (t, *J* = 1.9 Hz, $1H_b$), 8.31 (s, $1H_d$), 7.04 (dd, *J* = 8.2, 1.8 Hz, $1H_f$), 6.99 (d, *J* = 1.8 Hz, $1H_e$), 6.59 (d, *J* = 8.2 Hz, $1H_g$), 4.07 – 3.99 (m, 2H), 3.92 (t, *J* = 4.3 Hz, 2H), 3.84 (t, *J* = 4.3 Hz, 2H), 3.80 (t, *J* = 4.3 Hz, 2H), 3.75 – 3.58 (m, *J* = 3.1 Hz, 8H).

^{13}C NMR (126 MHz, $CDCl_3$) δ 150.90, 150.63, 149.55, 149.04, 145.98, 133.24, 133.06, 131.68, 131.05, 124.09, 123.49, 122.82, 120.80, 114.57, 113.47, 112.83, 107.90, 100.73, 70.76, 70.17, 70.10, 69.01, 68.96, 68.80, 68.44 (Some signals obscured due to C-F coupling).

HRMS (ESI+ve) m/z: 986.0599 ($[M+H]^+$, $C_{36}H_{24}F_{10}N_7O_{12}Te$ requires 986.0634).

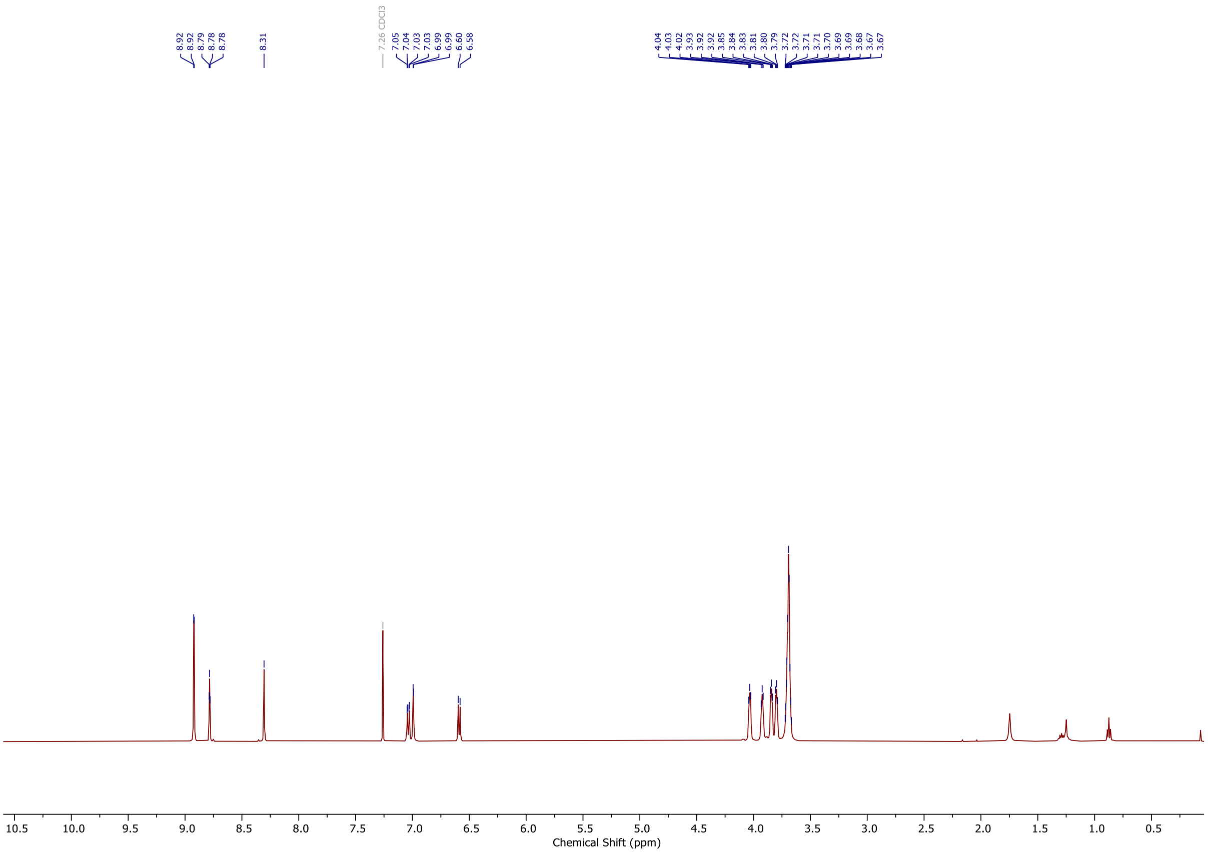

Figure S11. ^{1}H NMR spectrum of **S1** ($CDCl_3$, 500 MHz, 298K).

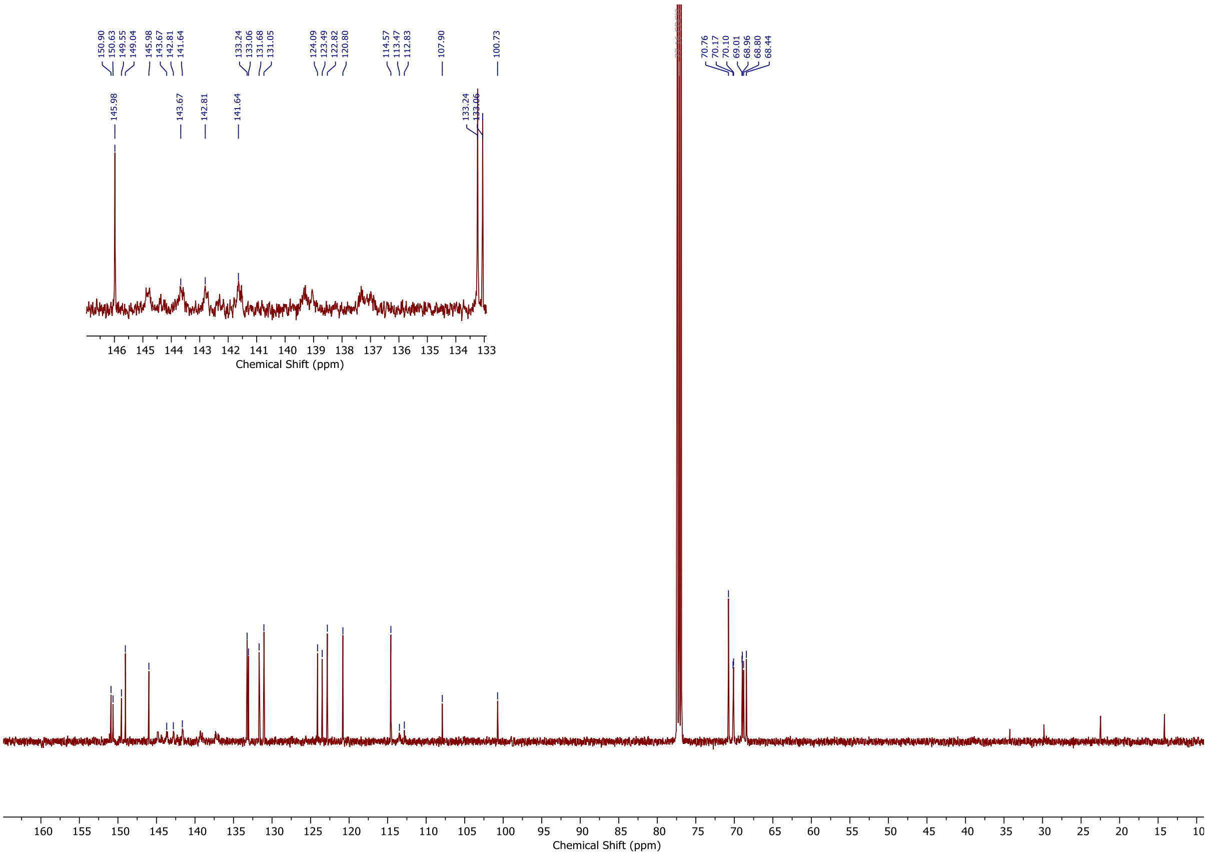

Figure S12. ^{13}C NMR spectrum of **S1** ($CDCl_3$, 126 MHz, 298K).

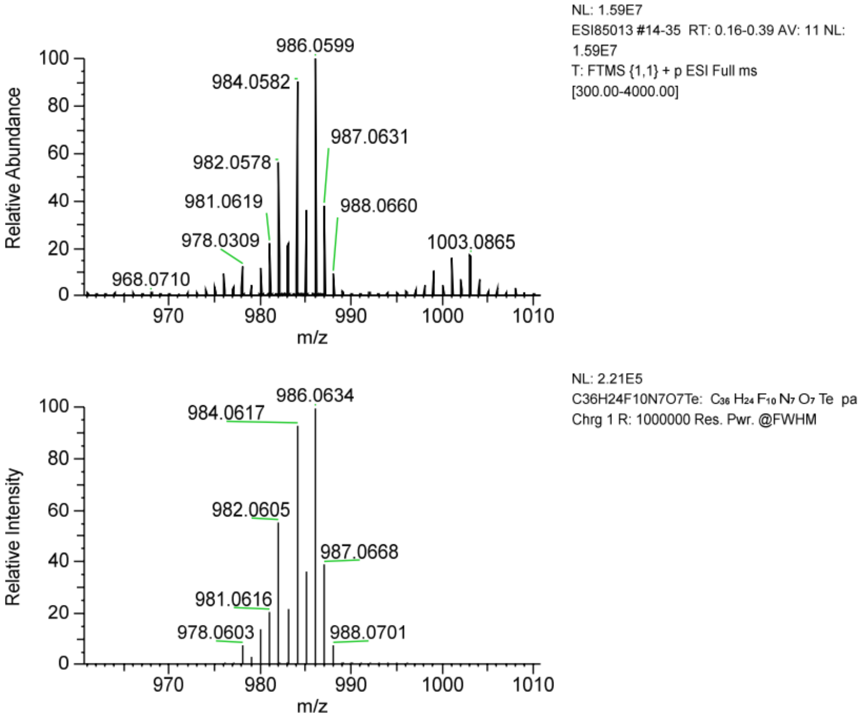

Figure S13. HRESI spectrum of **S1.**

NMR Spectroscopy Experiments

General Titration Protocol: In a typical ^{1}H NMR titration experiment, aliquots of the titrant were added to a 1:1 $CDCl_3$:CD_3CN (v/v) solution of the receptor and the spectrum recorded (typically) at 0, 0.2, 0.4, 0.6, 0.8, 1.0, 1.2, 1.4, 1.6, 1.8, 2.0, 2.5, 3.0, 4.0, 5.0, 7.0 and 10 equivalents.

[Host] = 1 mM

[$KBAr^F_4$] or [$NaBAr^F_4$] or [TBAX] = 50 mM

^{1}H NMR **Cation** or Anion **Binding Studies**

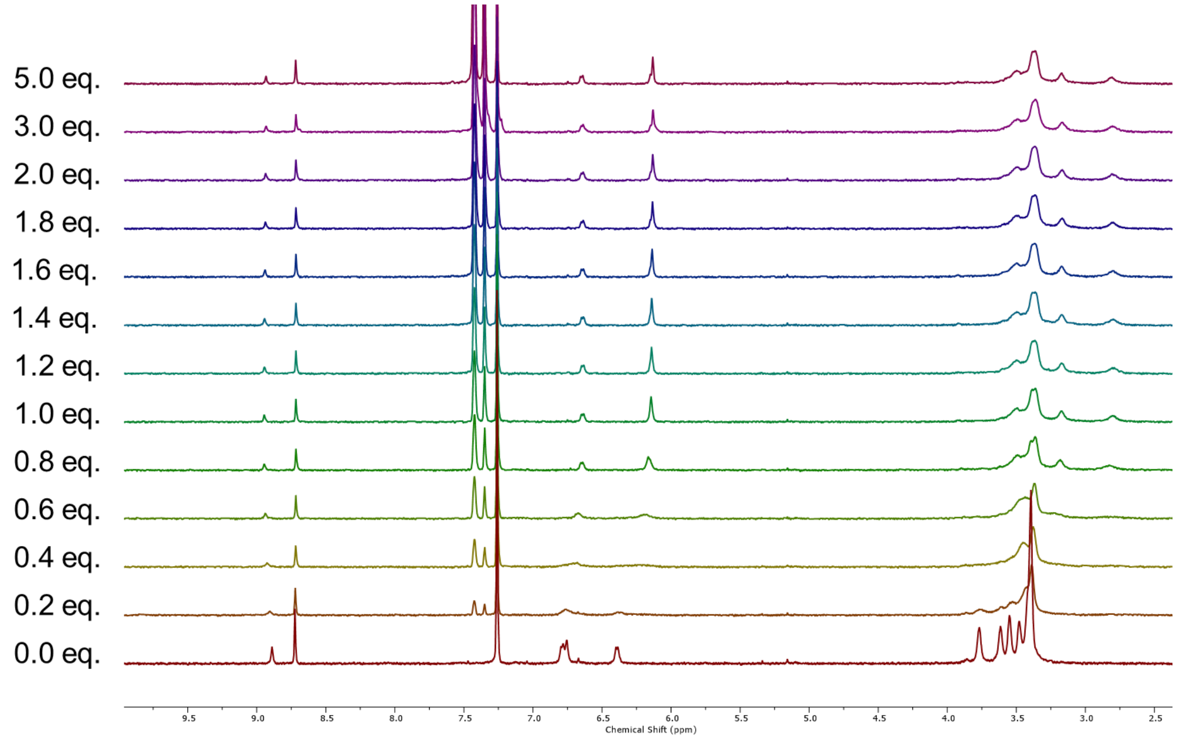

Figure S14. Stacked ^{1}H NMR titration spectra of **1·ChB^{PFP}** (1 mM) with $KBAr^{F}_{4}$ (50 mM) (1:1 CD_3CN:$CDCl_3$ (v/v), 500 MHz, 298K).

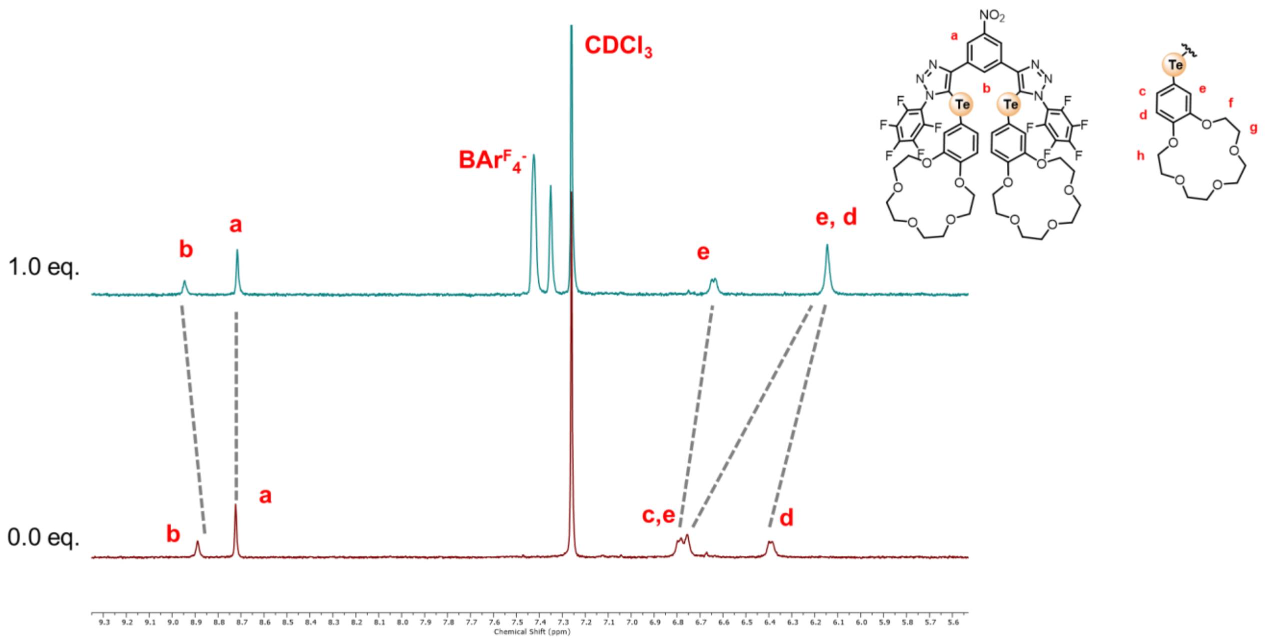

Figure S15. Stacked ^{1}H NMR titration spectra of **1·ChB^{PFP}** (1 mM) with $KBAr^{F}_{4}$ (50 mM) (1:1 CD_3CN:$CDCl_3$ (v/v), 500 MHz, 298K).

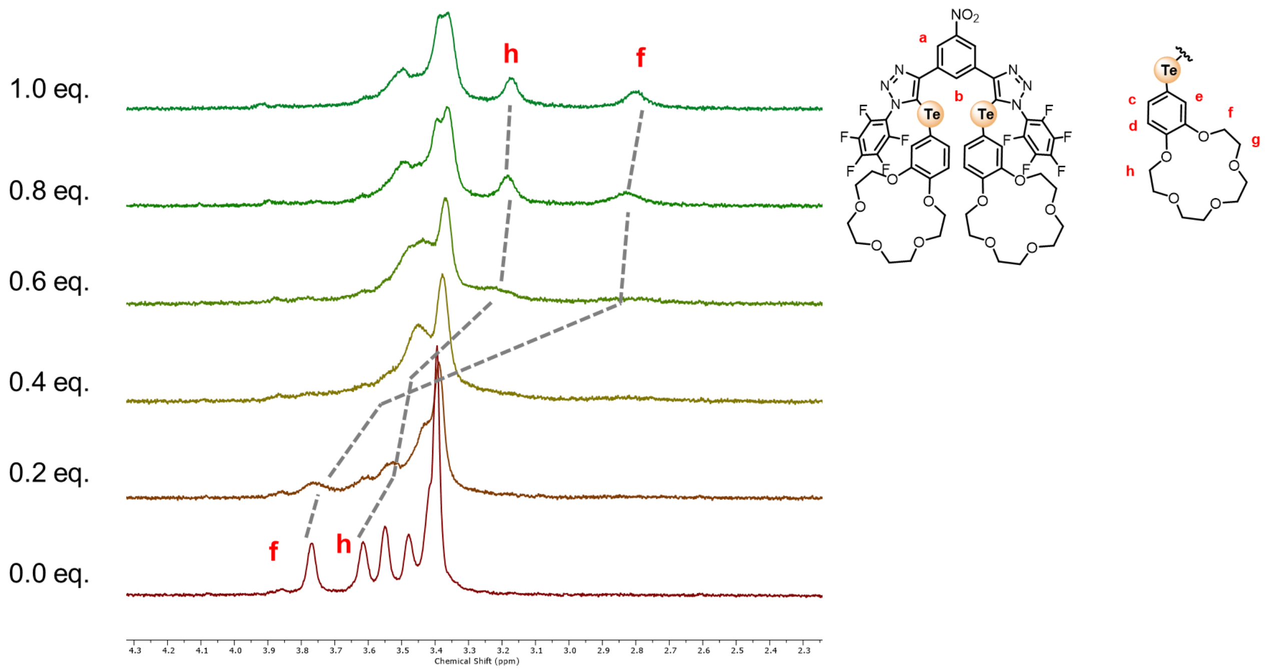

Figure S16. Stacked ^{1}H NMR titration spectra of **1·ChB^{PFP}** (1 mM) with $KBAr^{F}_{4}$ (50 mM) (1:1 CD_3CN:$CDCl_3$ (v/v), 500 MHz, 298K).

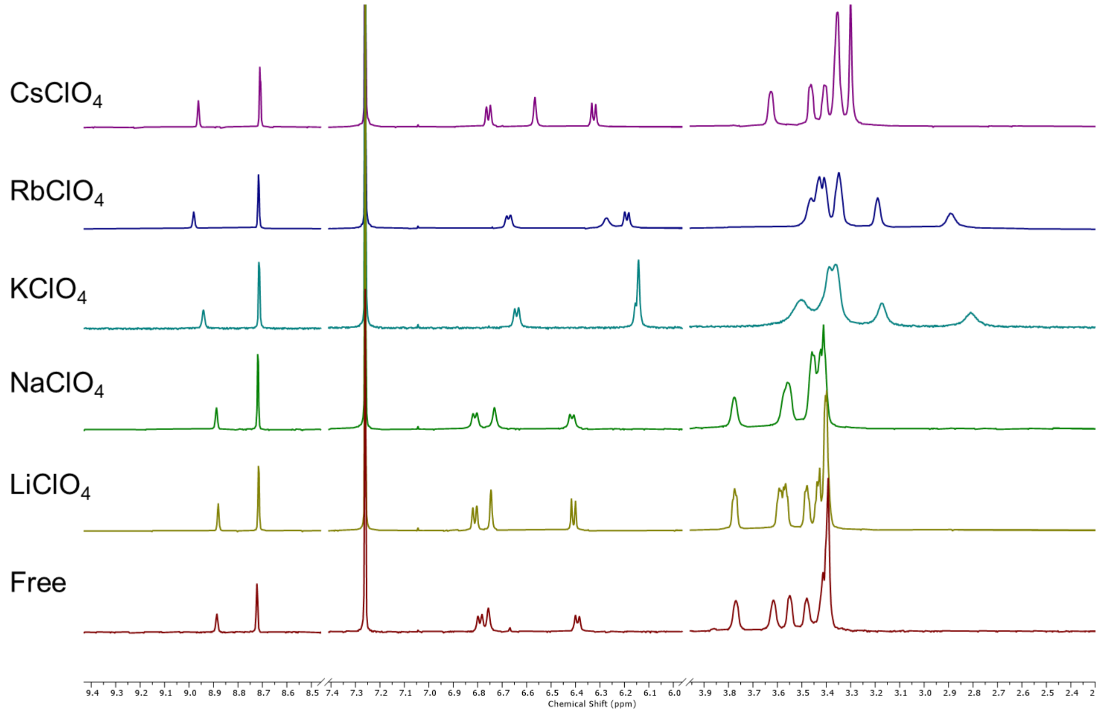

Figure S17. Stacked ^{1}H NMR titration spectra of **1·ChB^{PFP}** (1 mM) complexed with 1 equivalent of various alkali perchlorates (1:1 CD_3CN:$CDCl_3$ (v/v), 500 MHz, 298K).

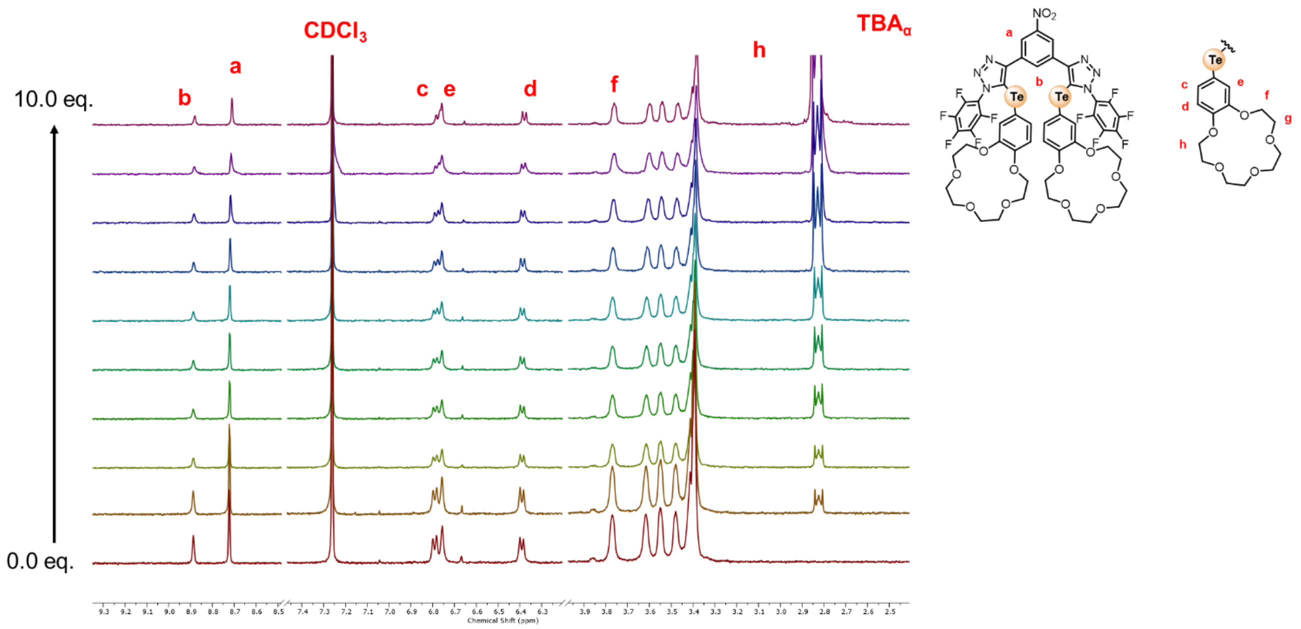

Figure S18. Stacked ^{1}H NMR titration spectra of **1·ChB^{PFP}** (1 mM) with TBACl (50 mM) (1:1 CD_3CN:$CDCl_3$ (v/v), 500 MHz, 298K).

^{1}H NMR Ion-Pair Binding Studies

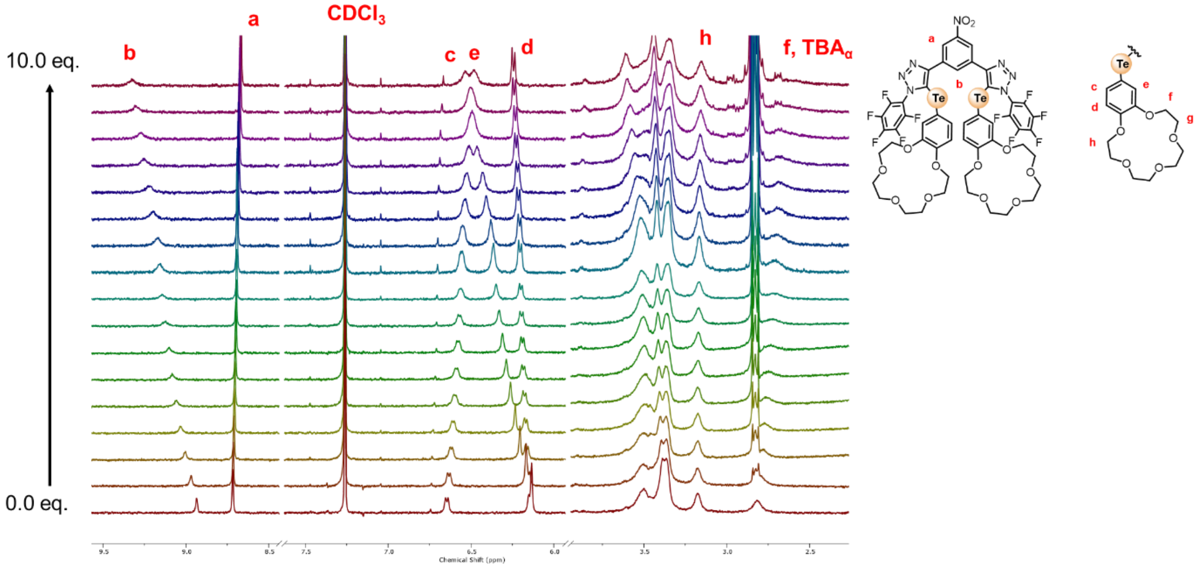

Figure S19. Stacked ^{1}H NMR titration spectra of **1·ChB^{PFP}** (1 mM) with TBACl (50 mM) in the presence of one equivalent of KPF_6 (1:1 CD_3CN:$CDCl_3$ (v/v), 500 MHz, 298K).

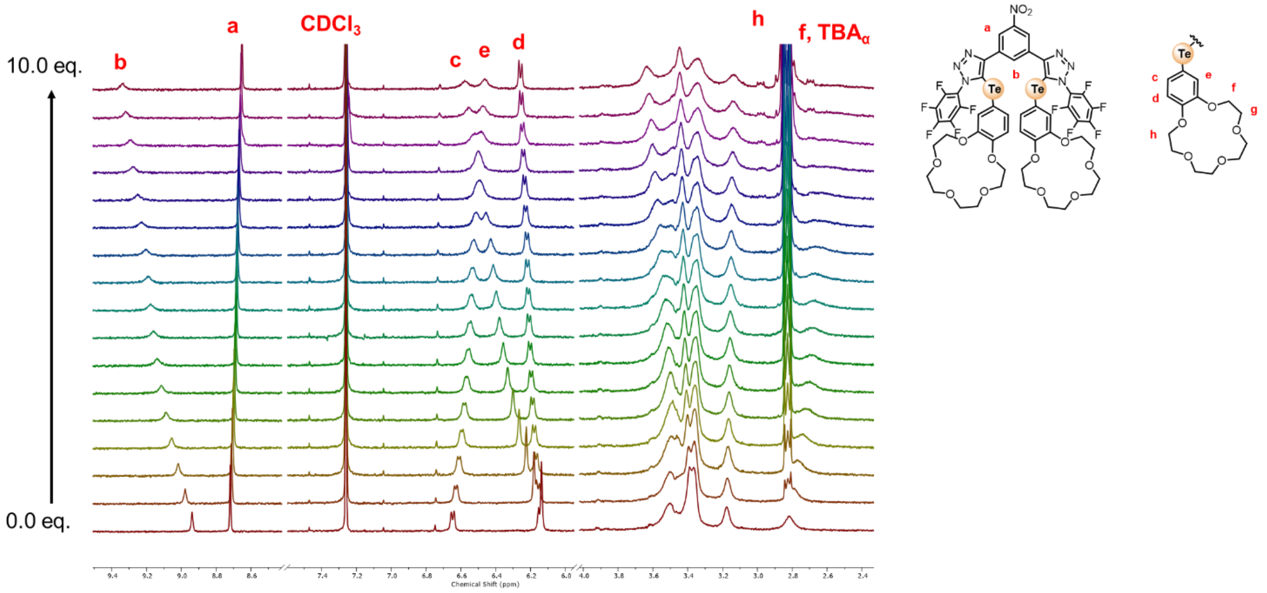

Figure S20. Stacked ^{1}H NMR titration spectra of **1•ChB^{PFP}** (1 mM) with TBABr (50 mM) in the presence of one equivalent of KPF_6 (1:1 CD_3CN:$CDCl_3$ (v/v), 500 MHz, 298K).

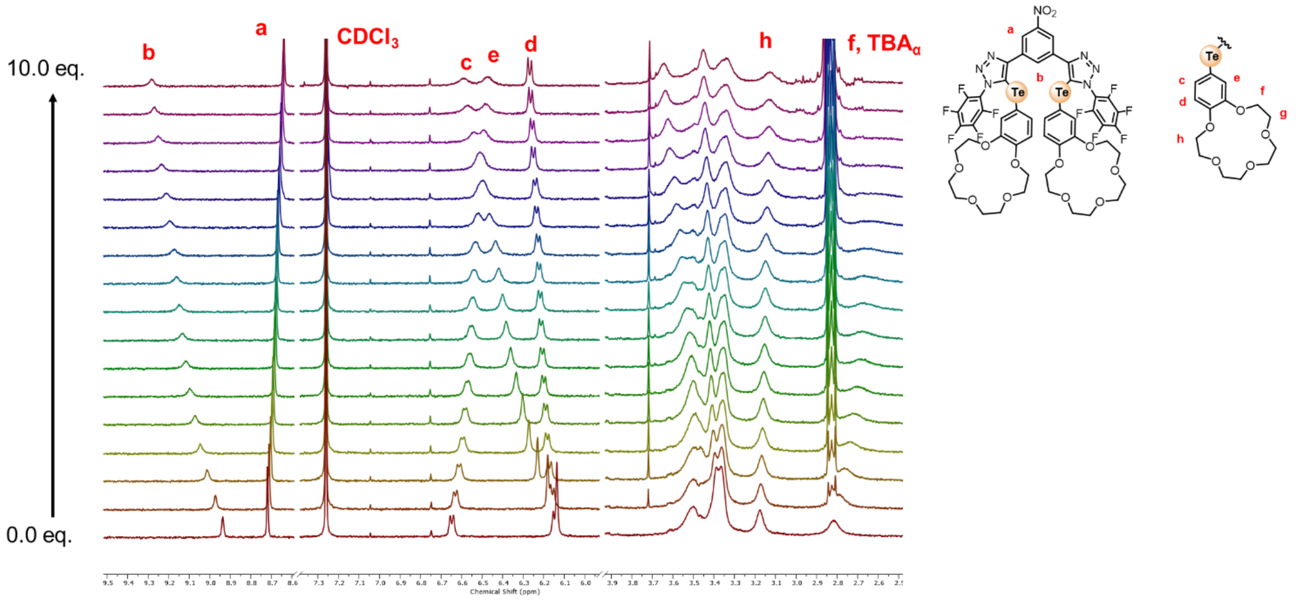

Figure S21. Stacked ^{1}H NMR titration spectra of **1•ChB^{PFP}** (1 mM) with TBAI (50 mM) in the presence of one equivalent of KPF_6 (1:1 CD_3CN:$CDCl_3$ (v/v), 500 MHz, 298K).

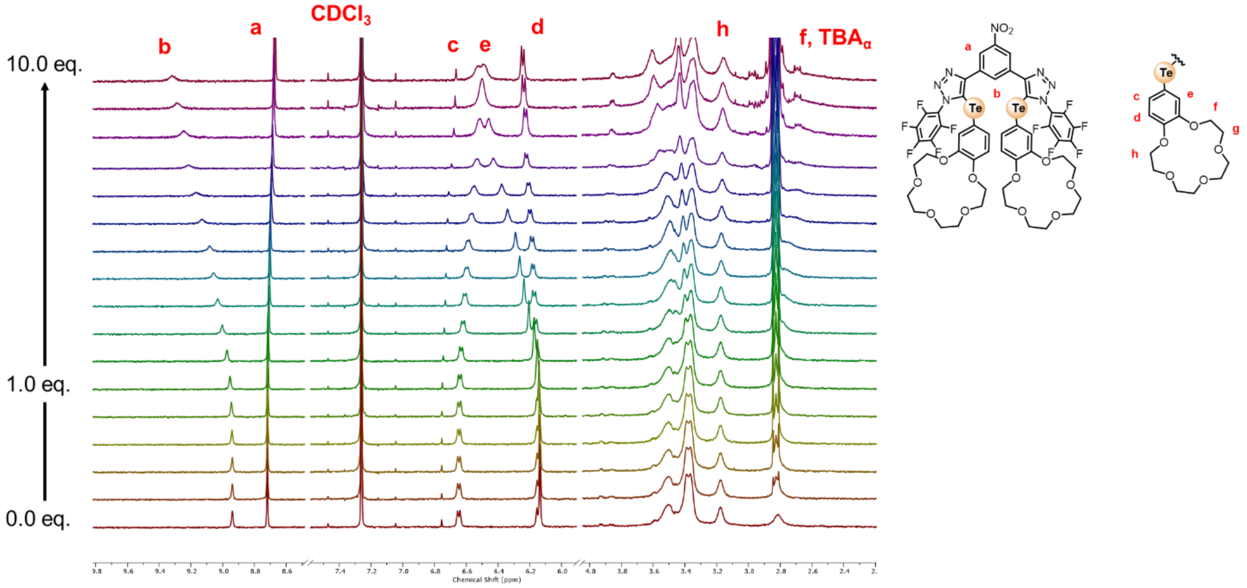

Figure S22. Stacked ^{1}H NMR titration spectra of **1·ChB**$^{\mathbf{PFP}}$ (1 mM) with TBACl (50 mM) in the presence of two equivalents of KPF_6 (1:1 CD_3CN:$CDCl_3$ (v/v), 500 MHz, 298K).

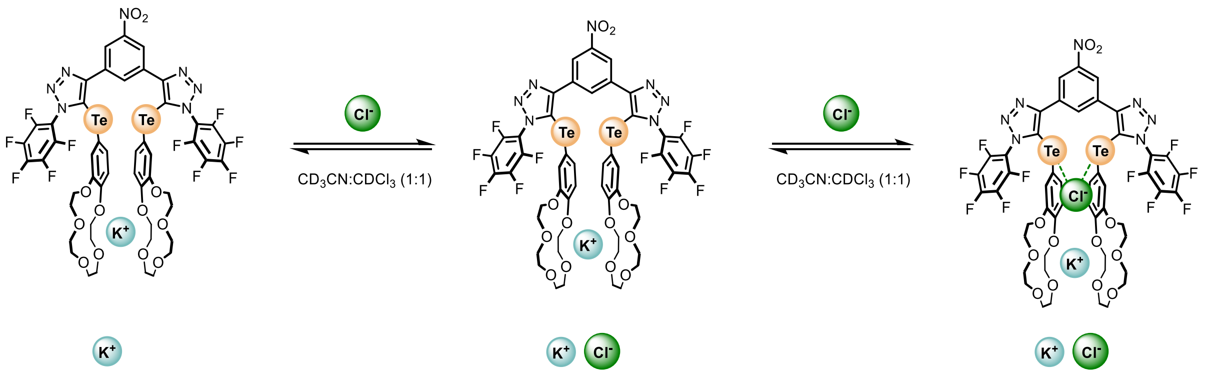

Figure S23. Proposed ion-pair binding equilibrium for **1·ChB**$^{\mathbf{PFP}}$ in the presence of two equivalents of KPF_6.

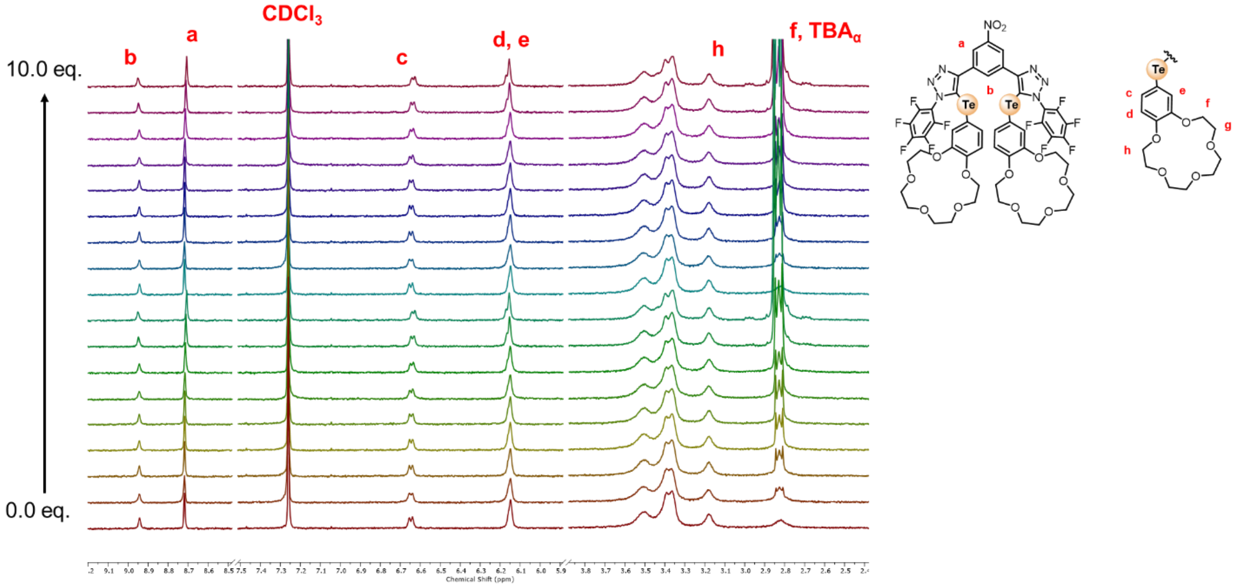

Figure S24. Stacked ^{1}H NMR titration spectra of **1·ChB**[PFP] (1 mM) with $TBAClO_4$ (50 mM) in the presence of one equivalent of KPF_6 (1:1 CD_3CN:$CDCl_3$ (v/v), 500 MHz, 298K).

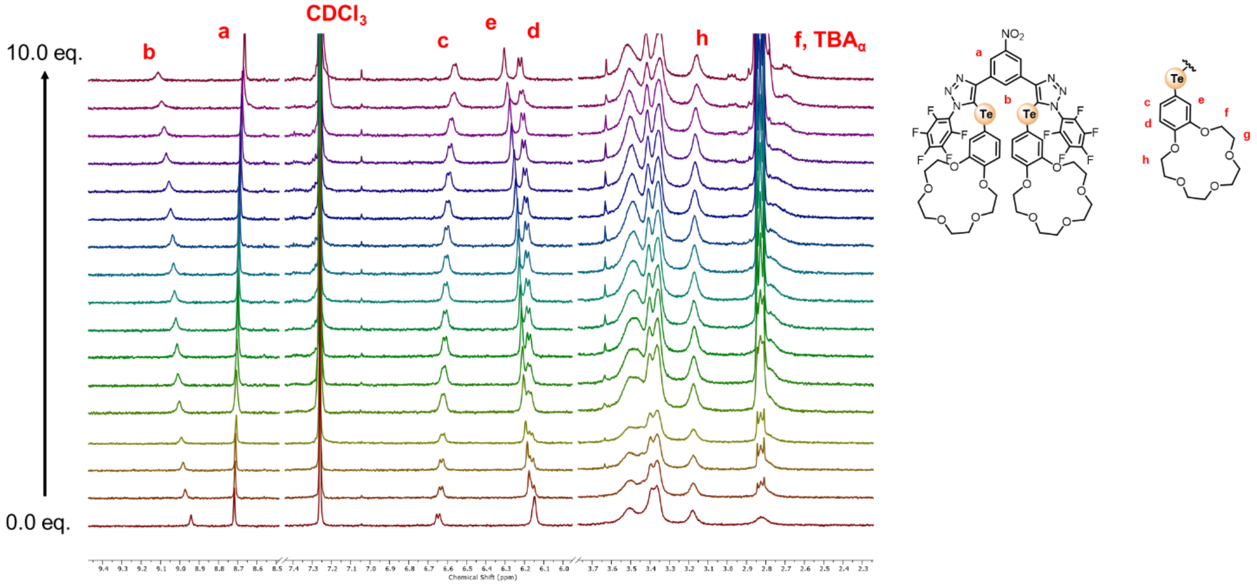

Figure S25. Stacked ^{1}H NMR titration spectra of **1·ChB**[PFP] (1 mM) with TBANCO (50 mM) in the presence of one equivalent of KPF_6 (1:1 CD_3CN:$CDCl_3$ (v/v), 500 MHz, 298K).

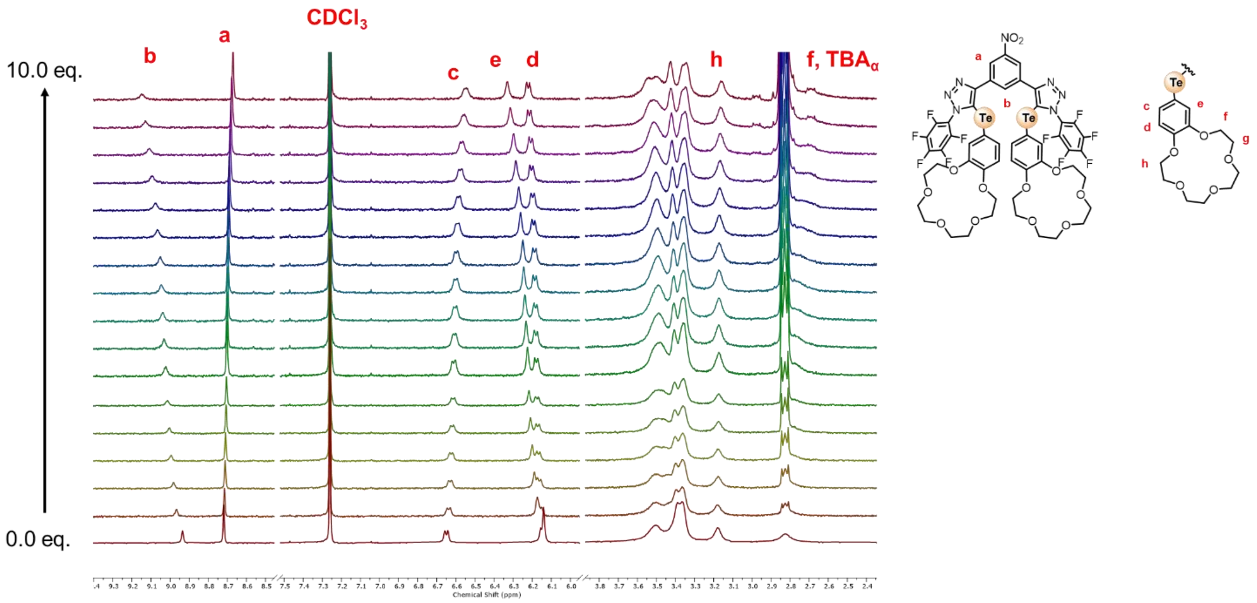

Figure S26. Stacked ^{1}H NMR titration spectra of **1·ChB**^{PFP} (1 mM) with TBANO$_2$ (50 mM) in the presence of one equivalent of KPF$_6$ (1:1 CD$_3$CN:CDCl$_3$ (v/v), 500 MHz, 298K).

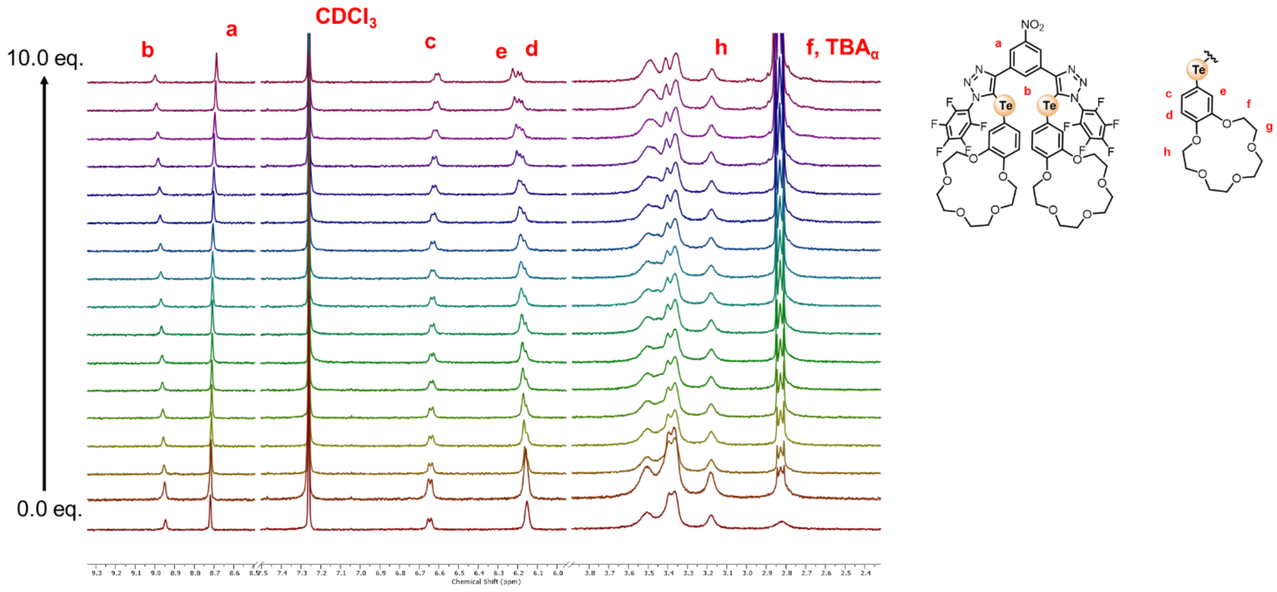

Figure S27. Stacked ^{1}H NMR titration spectra of **1·ChB**^{PFP} (1 mM) with TBANO$_3$ (50 mM) in the presence of one equivalent of KPF$_6$ (1:1 CD$_3$CN:CDCl$_3$ (v/v), 500 MHz, 298K).

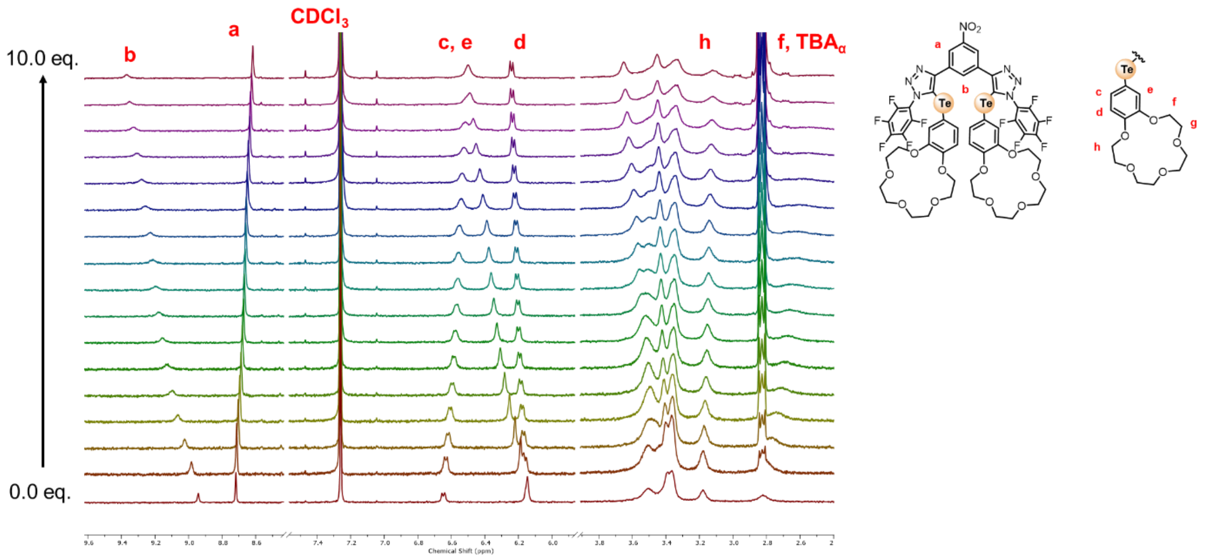

Figure S28. Stacked ^{1}H NMR titration spectra of **1·ChB^PFP^** (1 mM) with TBAOAc (50 mM) in the presence of one equivalent of KPF_6 (1:1 CD_3CN:$CDCl_3$ (v/v), 500 MHz, 298K).

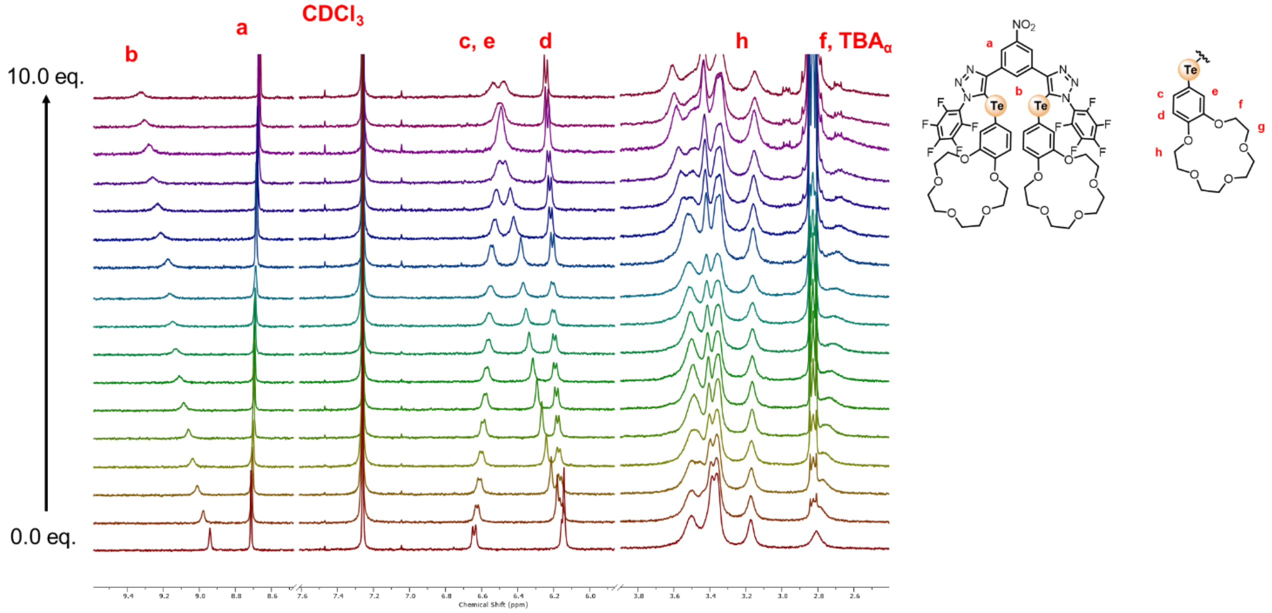

Figure S29. Stacked ¹H NMR titration spectra of **1·ChB^{PFP}** (1 mM) with TBACl (50 mM) in the presence of one equivalent of $CsClO_4$ (1:1 CD_3CN:$CDCl_3$ (v/v), 500 MHz, 298K).

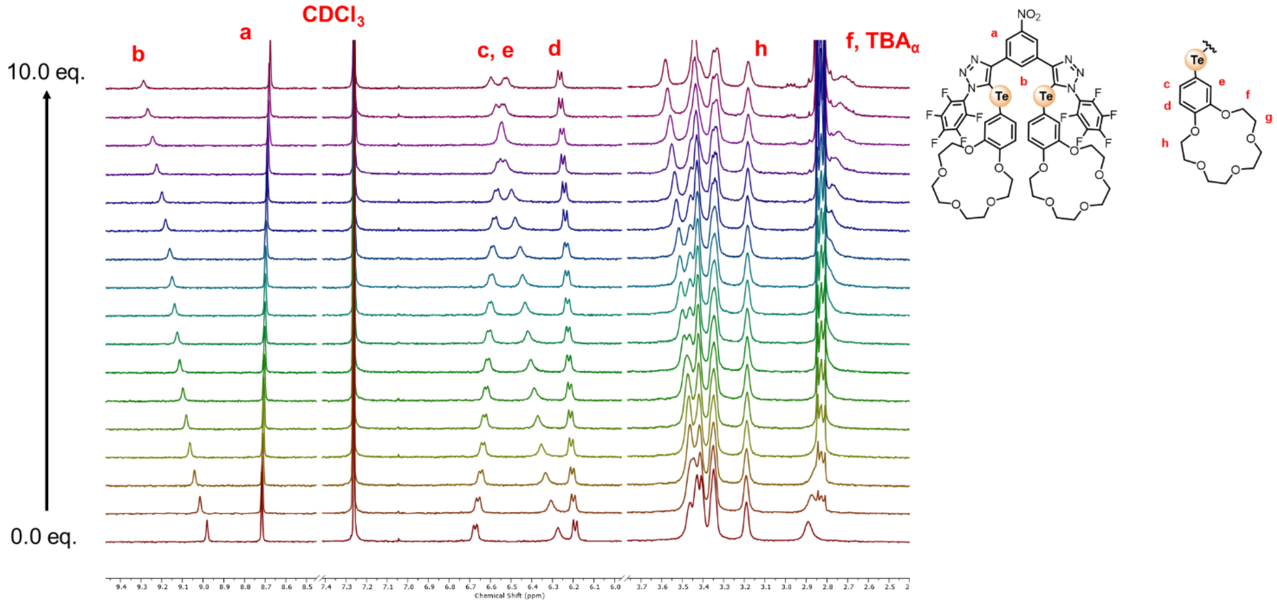

Figure S30. Stacked ¹H NMR titration spectra of **1·ChB^{PFP}** (1 mM) with TBACl (50 mM) in the presence of one equivalent of $RbClO_4$ (1:1 CD_3CN:$CDCl_3$ (v/v), 500 MHz, 298K).

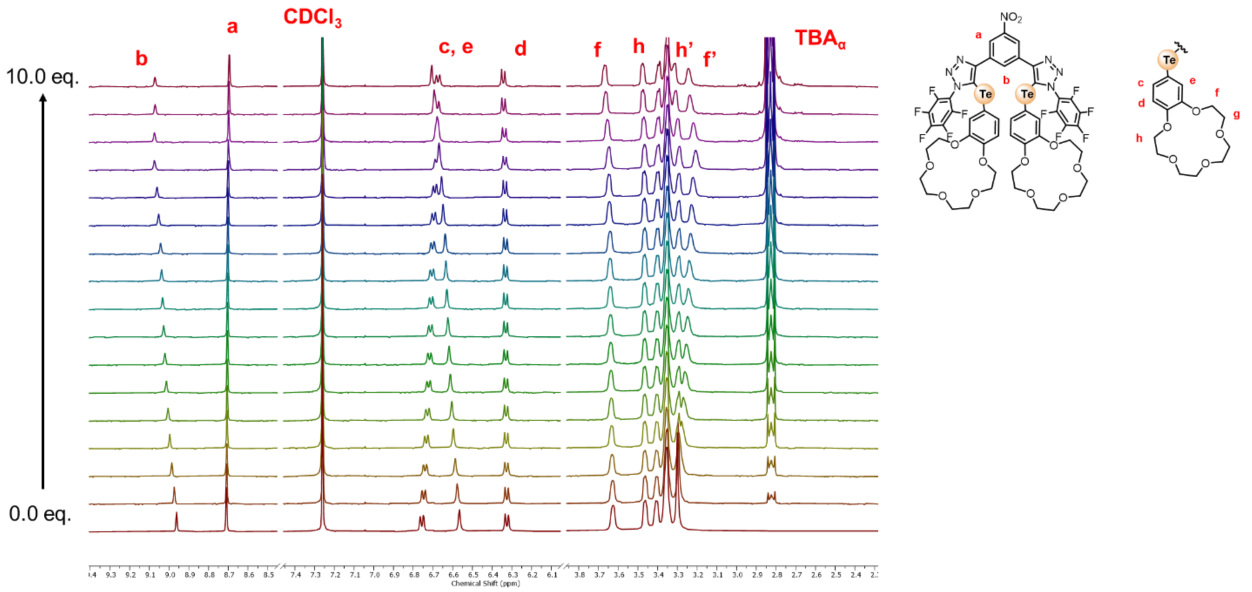

Figure S31. Stacked ^{1}H NMR titration spectra of **1·ChB^{PFP}** (1 mM) with TBACl (50 mM) in the presence of one equivalent of $KClO_4$ (1:1 CD_3CN:$CDCl_3$ (v/v), 500 MHz, 298K).

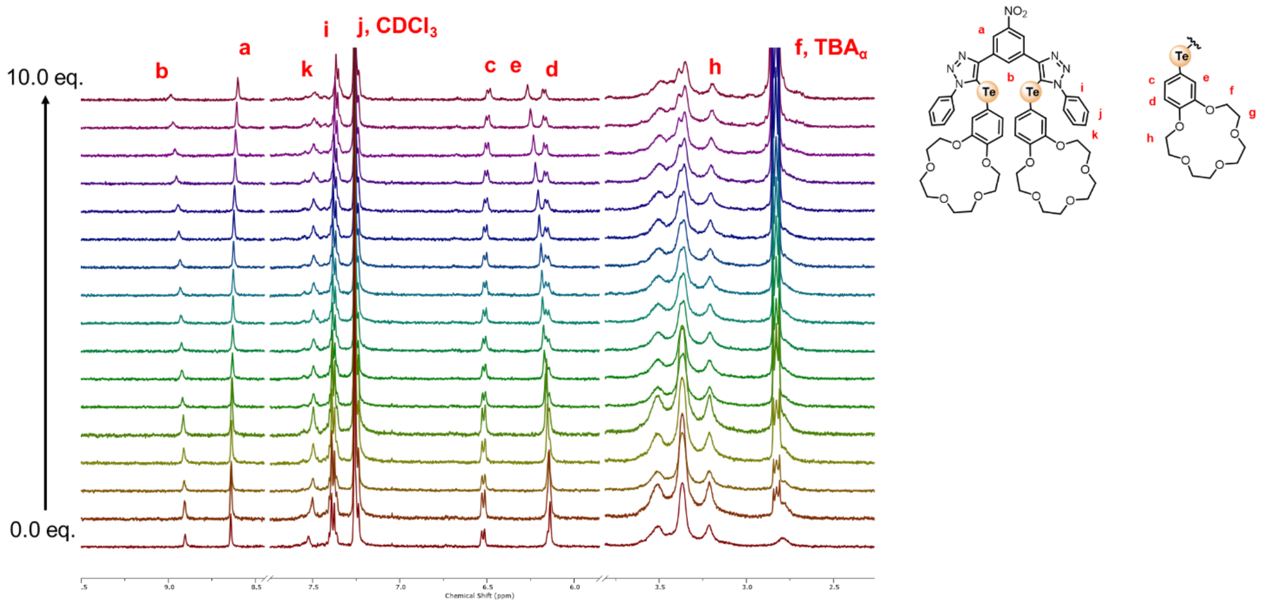

Figure S32. Stacked ^{1}H NMR titration spectra of **1·ChB^{Ph}** (1 mM) with TBACl (50 mM) in the presence of one equivalent of KPF_6 (1:1 CD_3CN:$CDCl_3$ (v/v), 500 MHz, 298K).

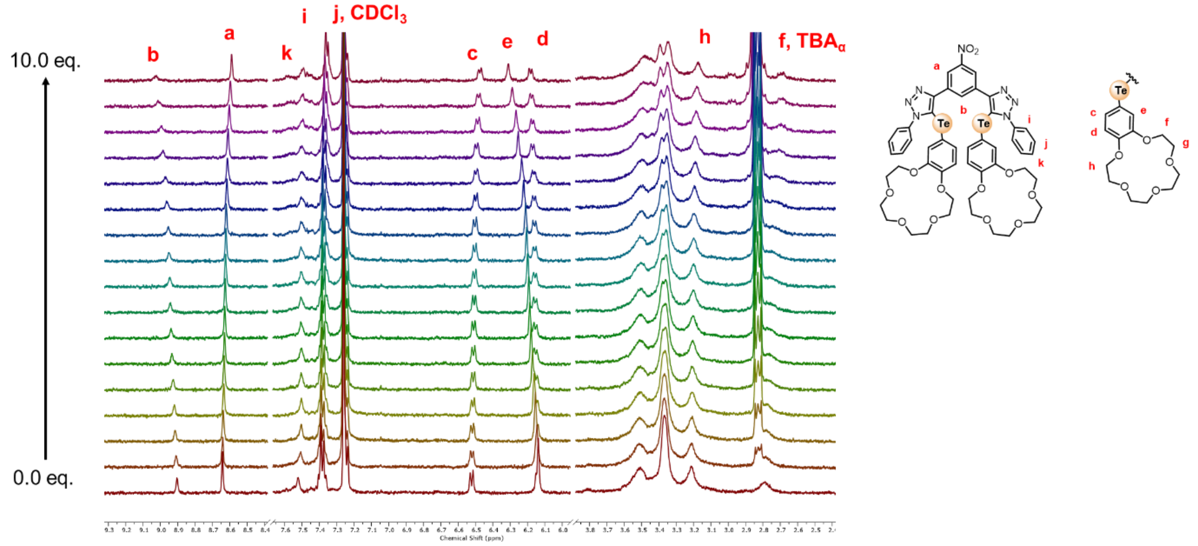

Figure S33. Stacked ^{1}H NMR titration spectra of **1·ChB^{Ph}** (1 mM) with TBABr (50 mM) in the presence of one equivalent of KPF_6 (1:1 CD_3CN:$CDCl_3$ (v/v), 500 MHz, 298K).

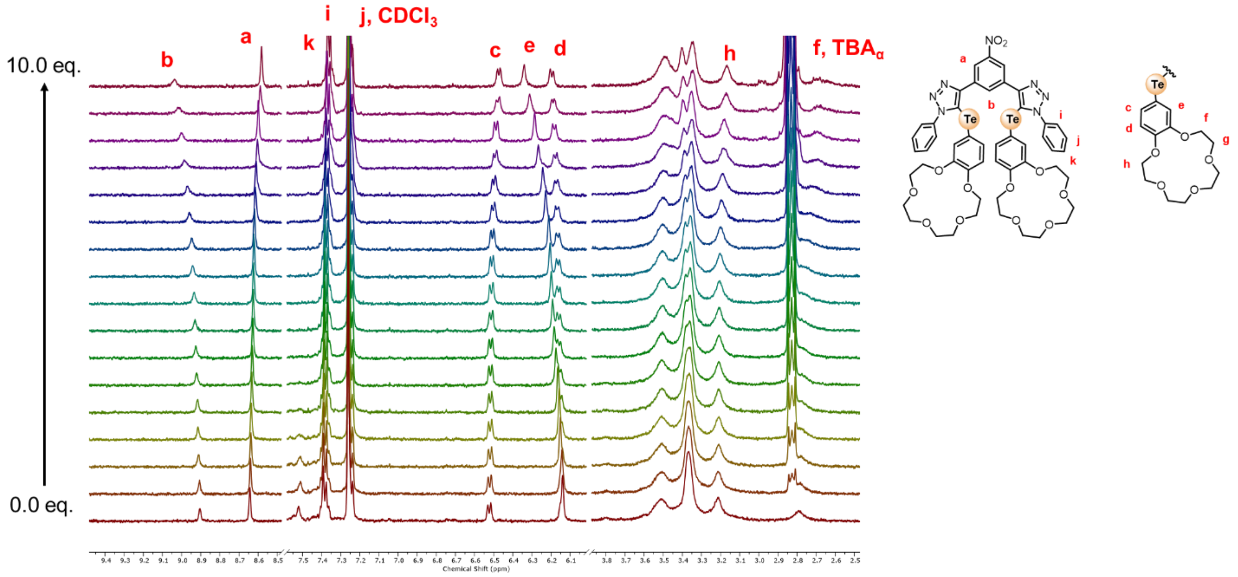

Figure S34. Stacked ^{1}H NMR titration spectra of **1·ChB^{Ph}** (1 mM) with TBAI (50 mM) in the presence of one equivalent of KPF_6 (1:1 CD_3CN:$CDCl_3$ (v/v), 500 MHz, 298K).

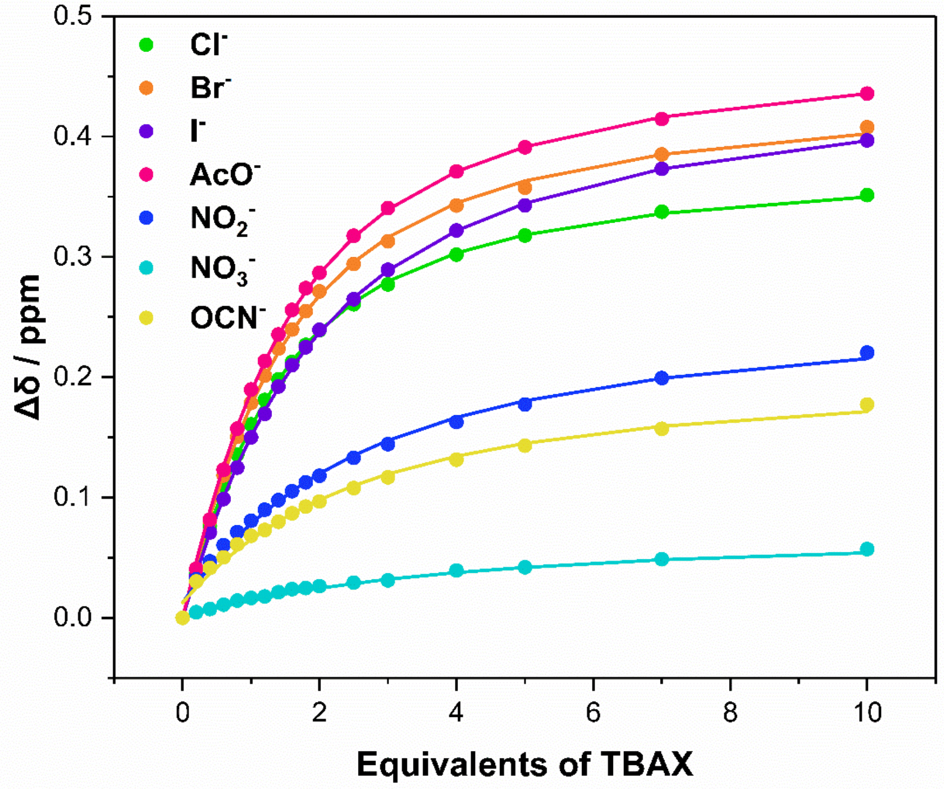

Figure S35. Anion binding isotherms for **1·ChB**^{PFP} in the presence of one equivalent of KPF_6, where circles represent experimental data and solid lines represent the fitted binding isotherm (1:1 CD_3CN:$CDCl_3$ (v/v), 500 MHz, 298K).

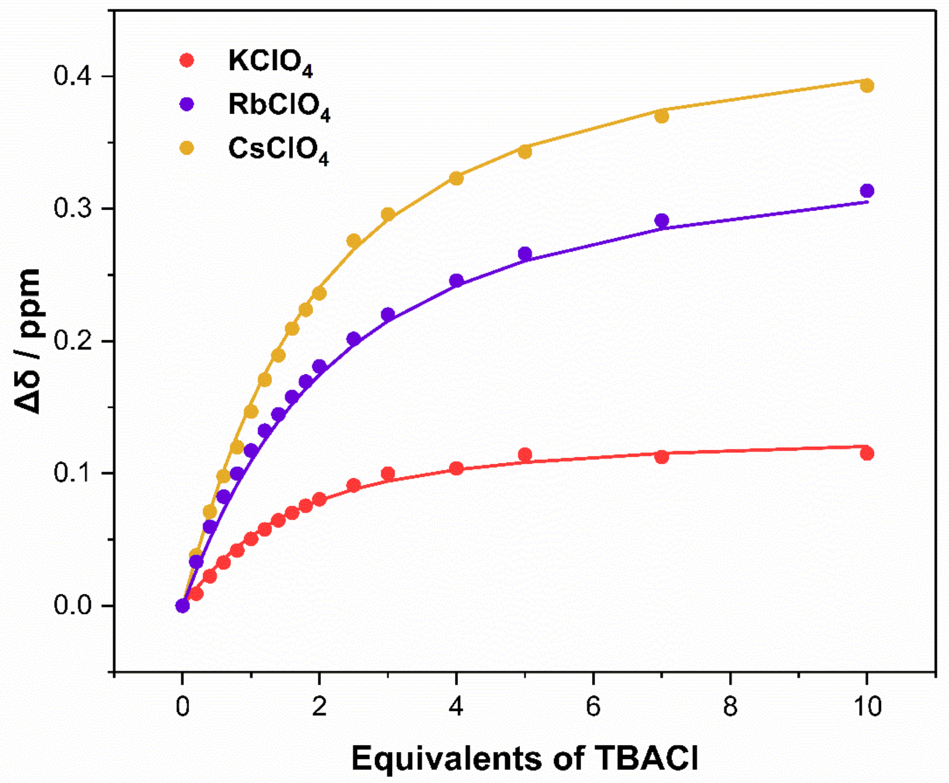

Figure S36. Chloride binding isotherms for **1·ChB**^{PFP} in the presence of one equivalent of $MClO_4$ (M= K, Rb, Cs), where circles represent experimental data and solid lines represent the fitted binding isotherm (1:1 CD_3CN:$CDCl_3$ (v/v), 500 MHz, 298K).

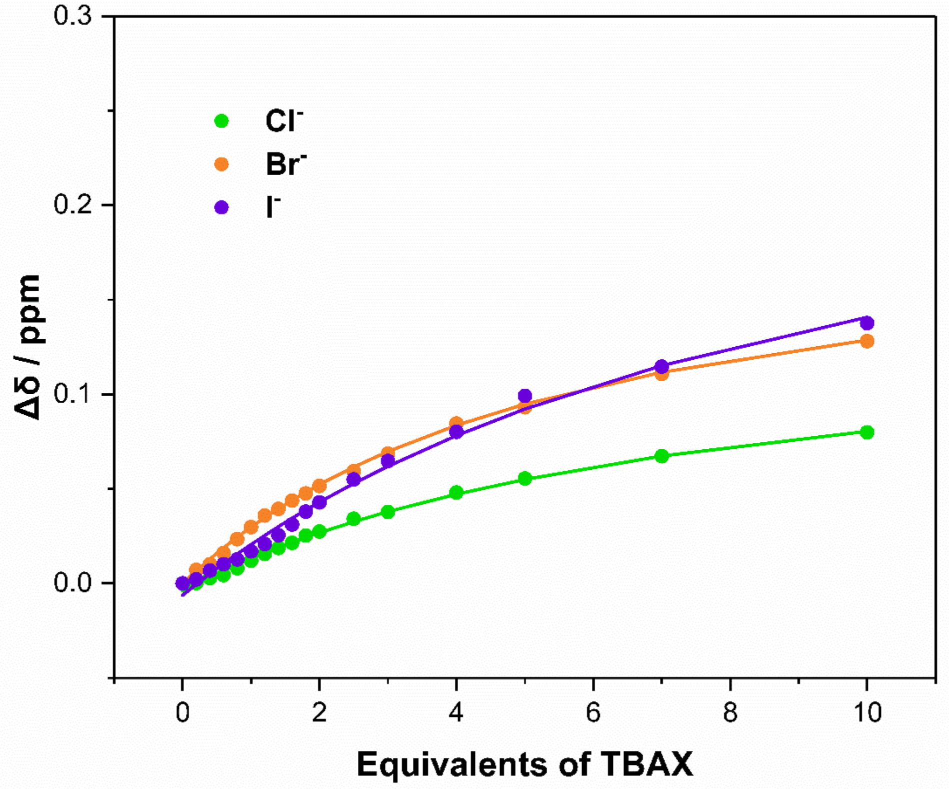

Figure S37. Anion binding isotherms for **1·ChB^{Ph}** in the presence of one equivalent of KPF_6, where circles represent experimental data and solid lines represent the fitted binding isotherm (1:1 CD_3CN:$CDCl_3$ (v/v), 500 MHz, 298K).

Table S1. Halide association constants for **1•ChB^PFP** from ^{1}H NMR titration (1:1 CD_3CN:$CDCl_3$ (v/v), 500 MHz, 298K).

Anion association constants of **1•ChB^PFP** in the presence and absence of equimolar KPF_6 /M^{-1} [a]		
Anion[b]	KPF_6	None
Cl^-	1,198	NB[c]
Br^-	1,080	NB[c]
I^-	709	NB[c]

[a] Determined from Bindfit analysis of the titration isotherm by monitoring of the chemical shift perturbation of internal aromatic signal b, error < 3 %. [b] Anions added their tetrabutylammonium salts. [c] NB = No binding, no observed chemical shift perturbations.

Table S2. Halide association constants for **1•ChB^Ph** from ^{1}H NMR titration (1:1 CD_3CN:$CDCl_3$ (v/v), 500 MHz, 298K).

Anion association constants of **1•ChB^Ph** in the presence and absence of equimolar KPF_6 /M^{-1} [a]		
Anion[b]	KPF_6	None
Cl^-	128	NB[c]
Br^-	190	NB[c]
I^-	93	NB[c]

[a] Determined from Bindfit analysis of the titration isotherm by monitoring of the chemical shift perturbation of internal aromatic signal b, error < 5%. [b] Anions added their tetrabutylammonium salts. [c] NB = No binding, no observed chemical shift perturbations.

Table S3. Halide association constants for **1•ChB**^PFP^ from ^{1}H NMR titration (1:1 CD_3CN:$CDCl_3$ (v/v), 500 MHz, 298K),

	Anion association constant of **1•ChB**^{Ph} in the presence and absence of equimolar $MClO_4$ /M^{-1} [a]				
Anion[b]	$LiClO_4$	$NaClO_4$	$KClO_4$	$RbClO_4$	$CsClO_4$
Cl^-	-[c]	-[c]	1,100	742	605

[a] Determined from Bindfit analysis of the titration isotherm by monitoring of the chemical shift perturbation of internal aromatic signal b. [b] Anions added their tetrabutylammonium salts. [c] ^{1}H NMR evidence demonstrates quantitative cation decomplexation and salt recombination.

Solid-Liquid Extraction (SLE) Procedure

A 2 mM solution of the receptor in $CDCl_3$ or CD_2Cl_2 (0.7 ml) was exposed to an excess (ca. 5 equivalents) of a microcrystalline sample of the alkali-metal salt. The mixture was sonicated for 10 minutes, after which the solution was removed and passed through a syringe filter and the ^1H NMR spectrum recorded. In the case of determining whether the emergence of new signals corresponded to the receptor:complex in slow exchange with the free receptor, the addition of known concentration of free receptor to the post extraction solution gave a spectrum which shows two sets of signals; receptor:complex and free receptor, indicating that these two species are indeed in slow exchange with each other.

Table S4. Summarised Solid-liquid extraction efficiencies for **1·ChB^{PFP}** and various potassium salts in $CDCl_3$.

Salt	KF	KCl	KBr	KI	$KClO_4$	KPF_6	KSCN	KNO_3
Extraction[a]	-[b]	47%	>95%[c]	>95%[c]	50%	>95%[c]	>95%[c]	35%

[a] Extraction as determined by integration of ^1H NMR spectrum resonances of the internal aromatic signals corresponding to free and complexed receptor. [b] Receptor decomposition. [c] No signal corresponding to free receptor observed.

Table S5. Summarised Solid-liquid extraction efficiencies for **1·ChB^{PFP}** and various potassium salts in CD_2Cl_2.

Salt	KCl	KBr	KI
Extraction[a]	40%	>95%[b]	>95%[b]

[a] Extraction as determined by integration of ^1H NMR spectrum resonances of the internal aromatic signals corresponding to free and complexed receptor. [b] No signal corresponding to free receptor observed.

Table S6. Summarised Solid-liquid extraction efficiencies for **1·ChB^{PFP}** and various alkali metal chloride salts in $CDCl_3$.

Salt	LiCl	NaCl	KCl	RbCl	CsCl
Extraction[a]	0%[b]	0%[b]	47%	10%	0%[b]

[a] Extraction as determined by integration of ^1H NMR spectrum resonances of the internal aromatic signals corresponding to free and complexed receptor. [b] No signal perturbations observed.

Table S7. Summarised Solid-liquid extraction efficiencies for **1·ChB^{PFP}** and various alkali metal salts in $CDCl_3$.

Salt	RbI	$RbClO_4$	CsI	$CsClO_4$
Extraction[a]	>95%[b]	0%[c]	>95% [b]	0%[c]

[a] Extraction as determined by integration of ^{1}H NMR spectrum resonances corresponding to free and complexed receptor. [b] No signal corresponding to free receptor observed. [c] No signal perturbations observed.

Table S8. Summarised Solid-liquid extraction efficiencies for **1·ChB^{Ph}** and various potassium salts in $CDCl_3$.

Salt	KCl	KBr	KI
Extraction[a]	0%[b]	>95%[c]	>95%[c]

[a] Extraction as determined by integration of ^{1}H NMR spectrum resonances of the internal aromatic signals corresponding to free and complexed receptor. [b] No signal perturbations observed. [c] No signal corresponding to free receptor observed.

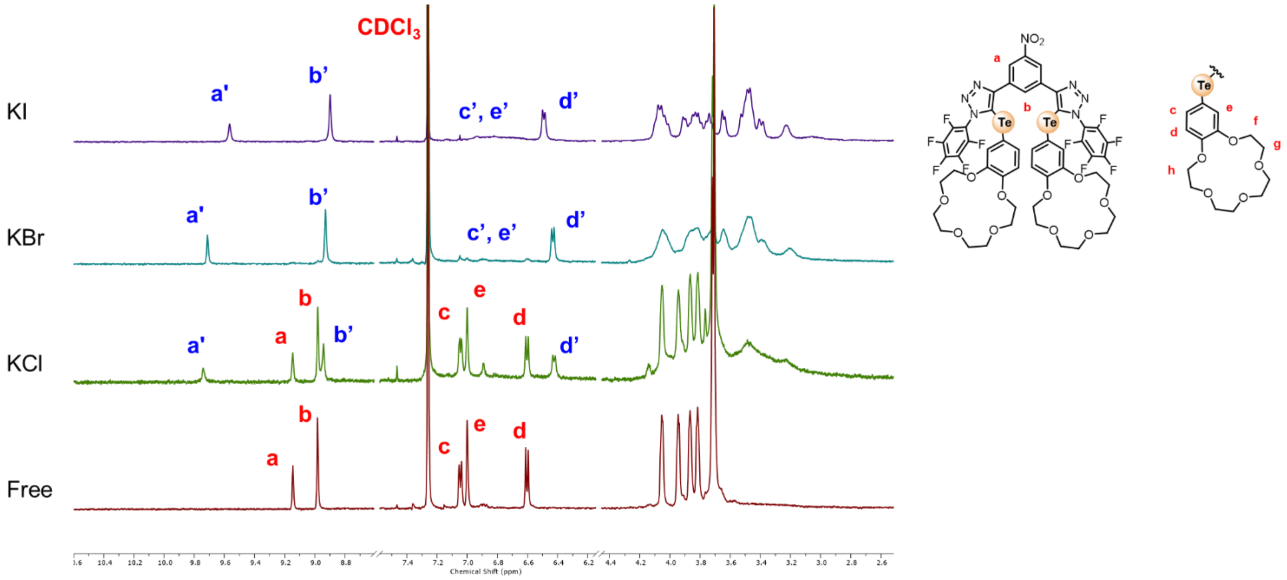

Figure S38. Representative example of the calculation of receptor loading by integration of the signals corresponding to **1·ChB**^{PFP} (red) and [**1·ChB**^{PFP} + MX] (blue) from a SLE ^{1}H NMR extraction experiment ($CDCl_3$, 500 MHz, 298 K).

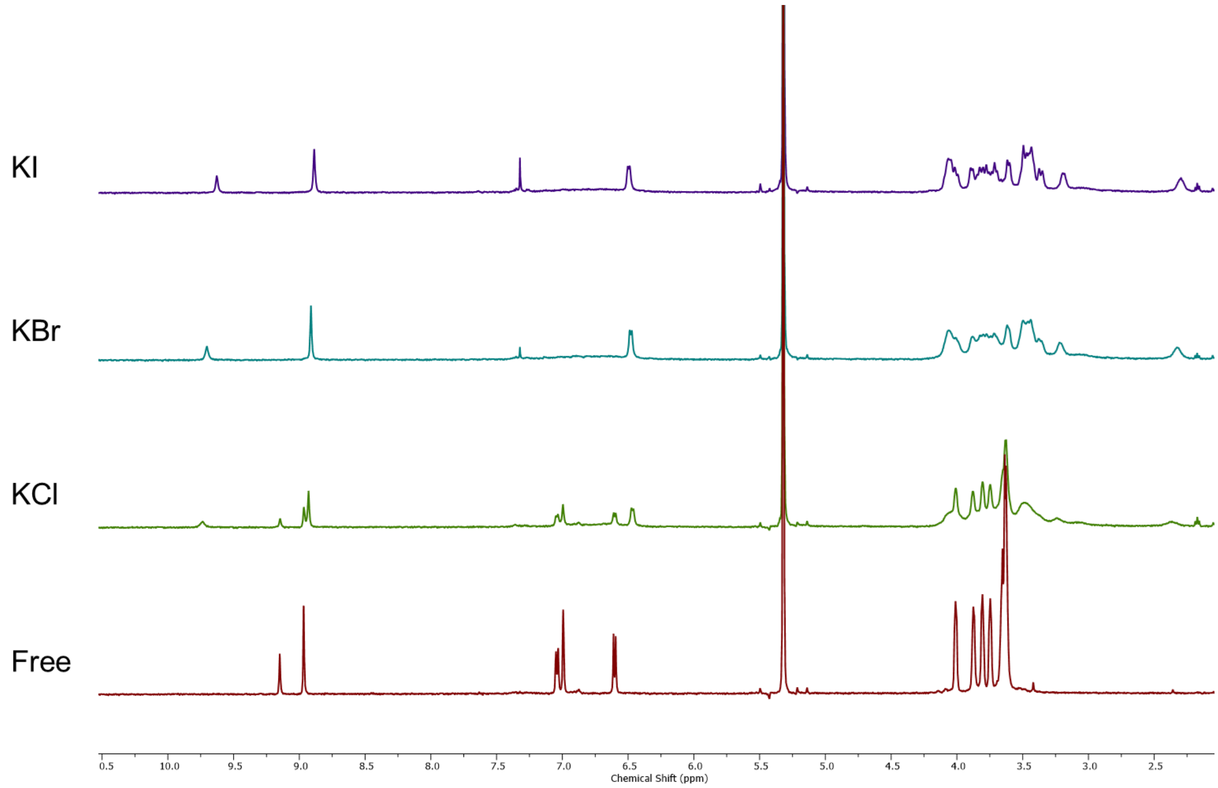

Figure S39. ^{1}H NMR spectrum from SLE extraction experiment of **1·ChB**^{PFP} (CD_2Cl_2, 500 MHz, 298K).

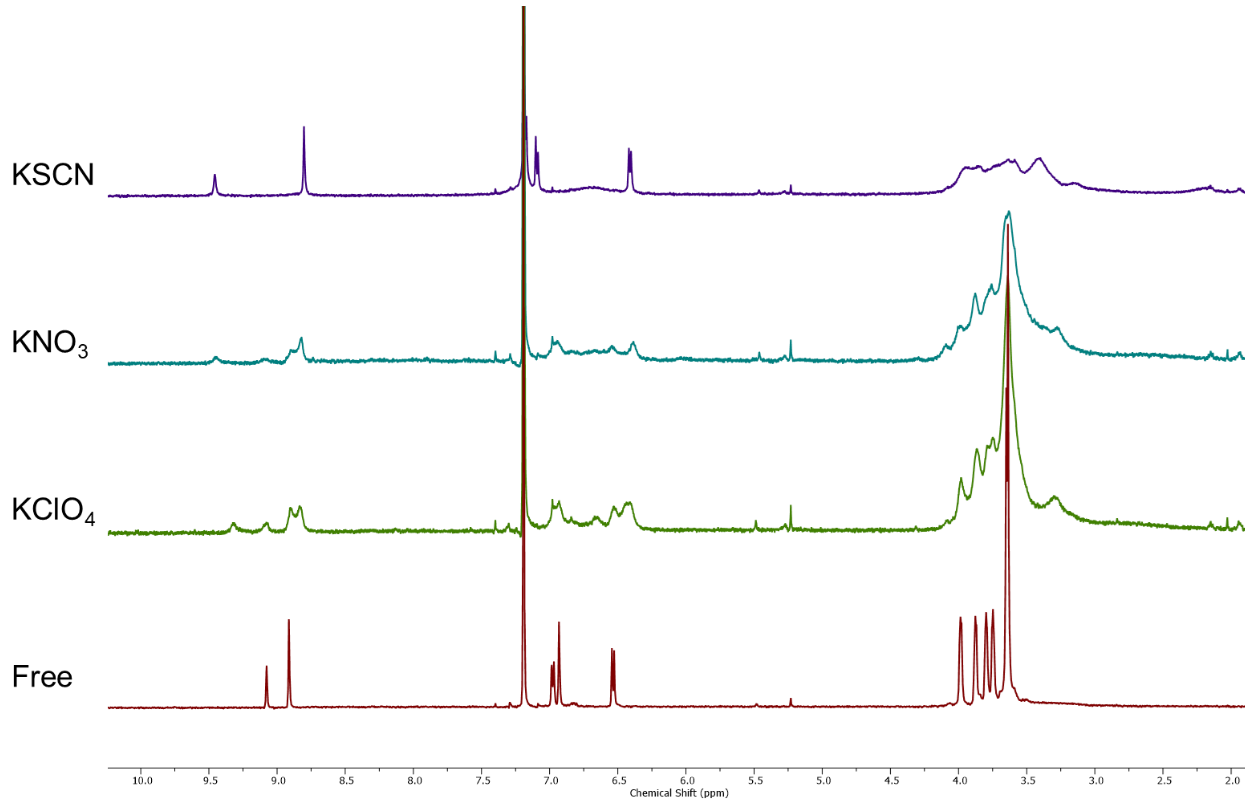

Figure S40. ^1H NMR spectrum from SLE extraction experiment of **1·ChB**^{PFP} ($CDCl_3$, 500 MHz, 298K).

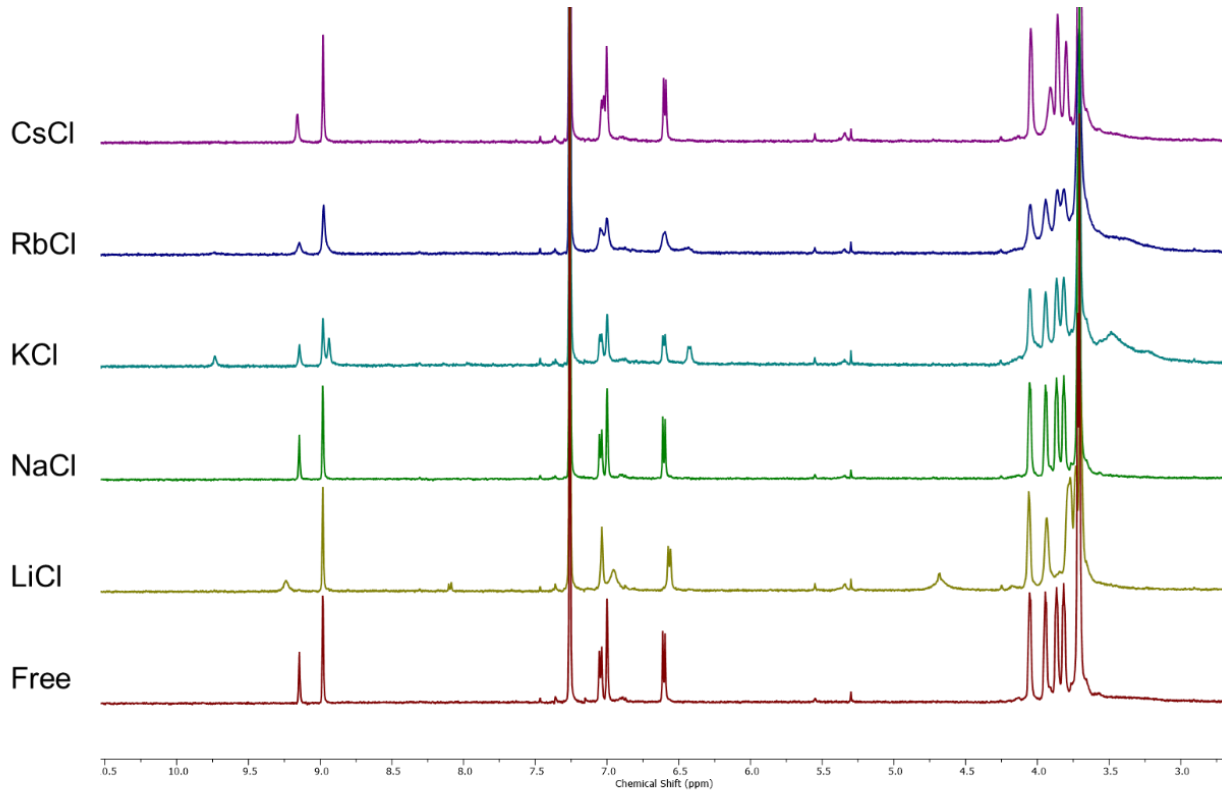

Figure S41. ^1H NMR spectrum from SLE extraction experiment of **1·ChB**^{PFP} ($CDCl_3$, 500 MHz, 298K).

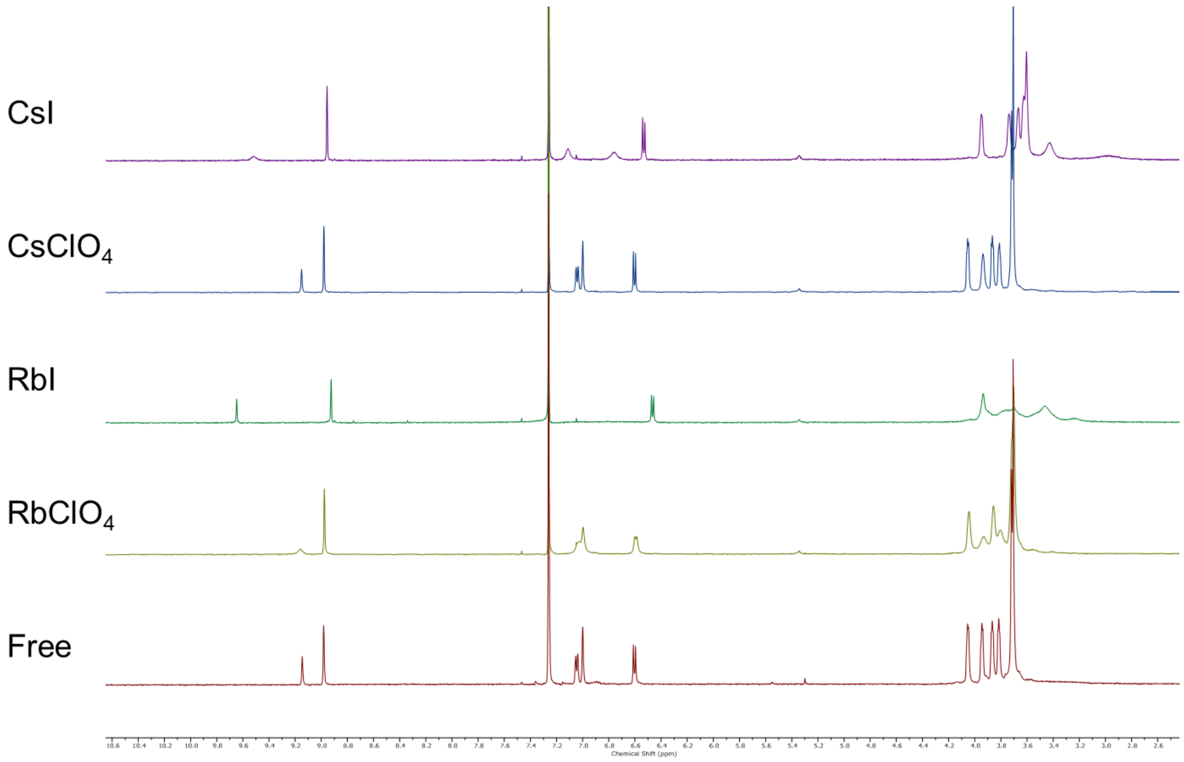

Figure S42. ^{1}H NMR spectrum from SLE extraction experiment of **1·ChB**^{PFP} ($CDCl_3$, 500 MHz, 298K).

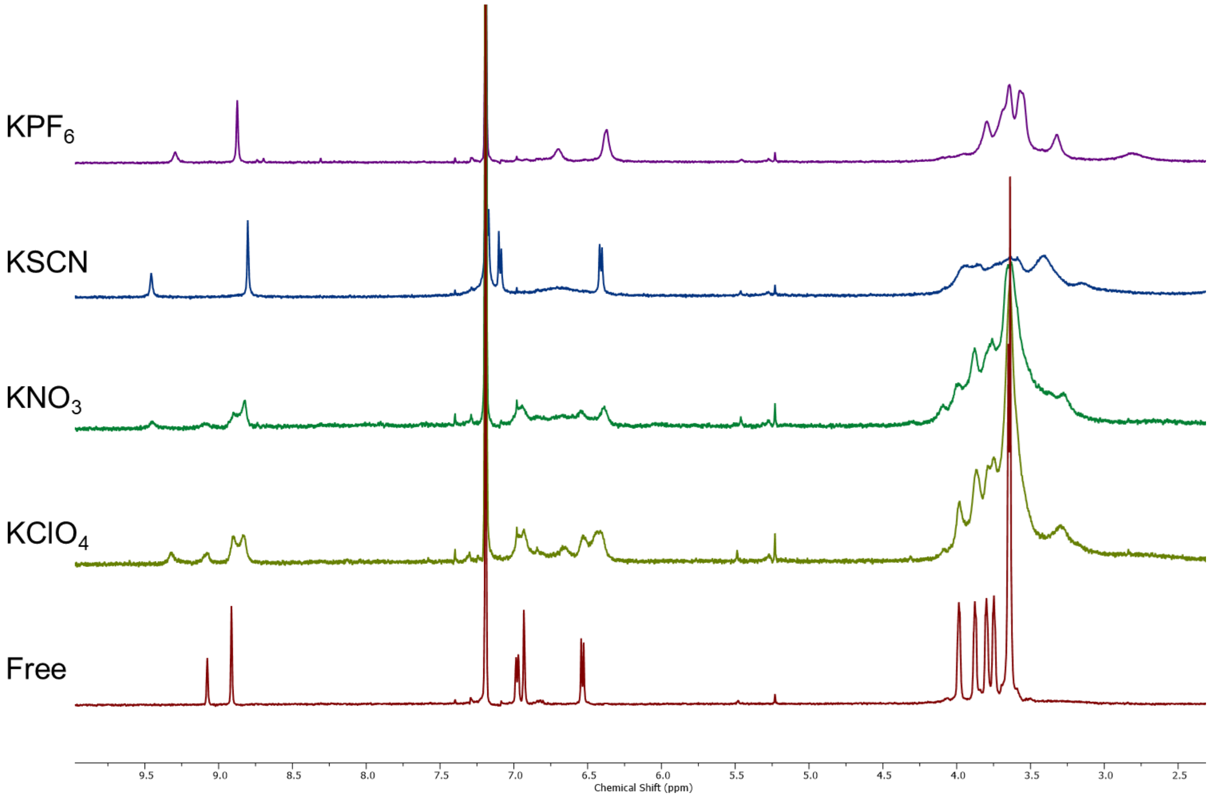

Figure S43. ^{1}H NMR spectrum from SLE extraction experiment of **1·ChB**^{PFP} ($CDCl_3$, 500 MHz, 298K).

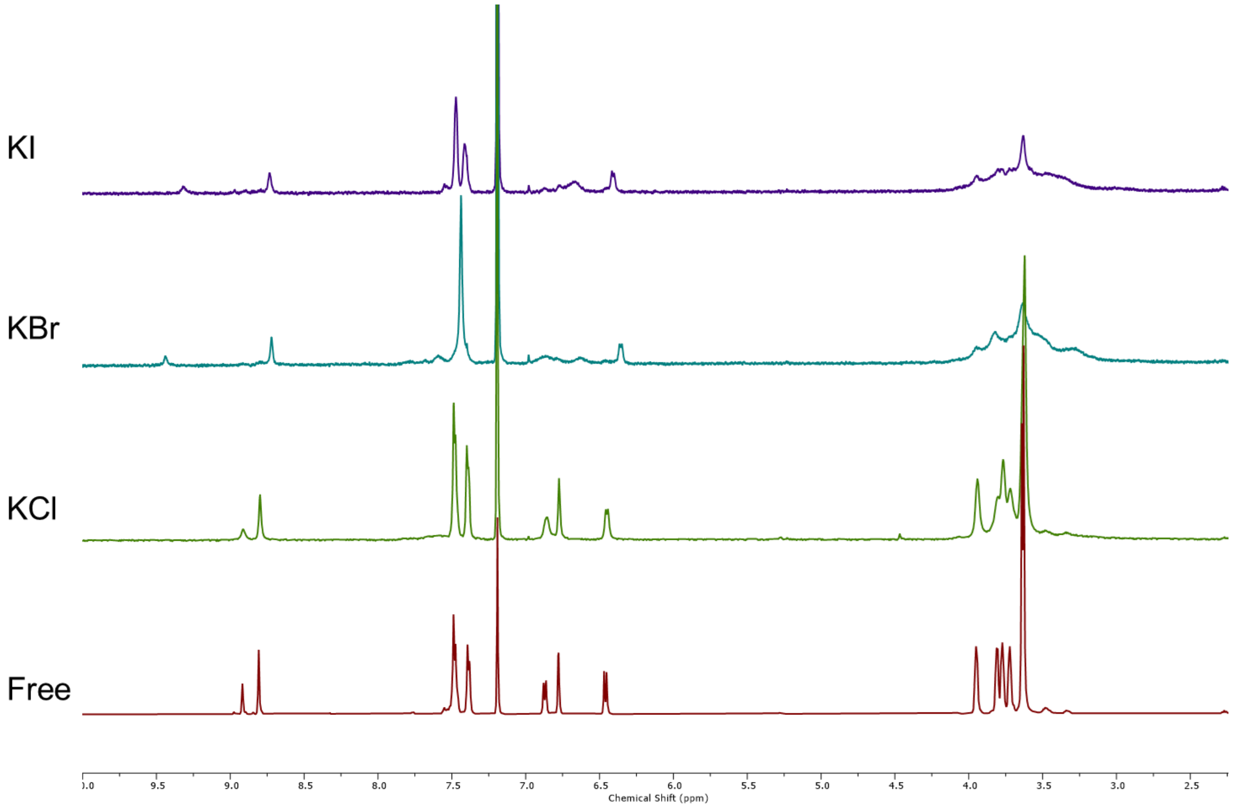

Figure S44. ^{1}H NMR spectrum from SLE extraction experiment of **1·ChB**^{Ph} ($CDCl_3$, 500 MHz, 298K).

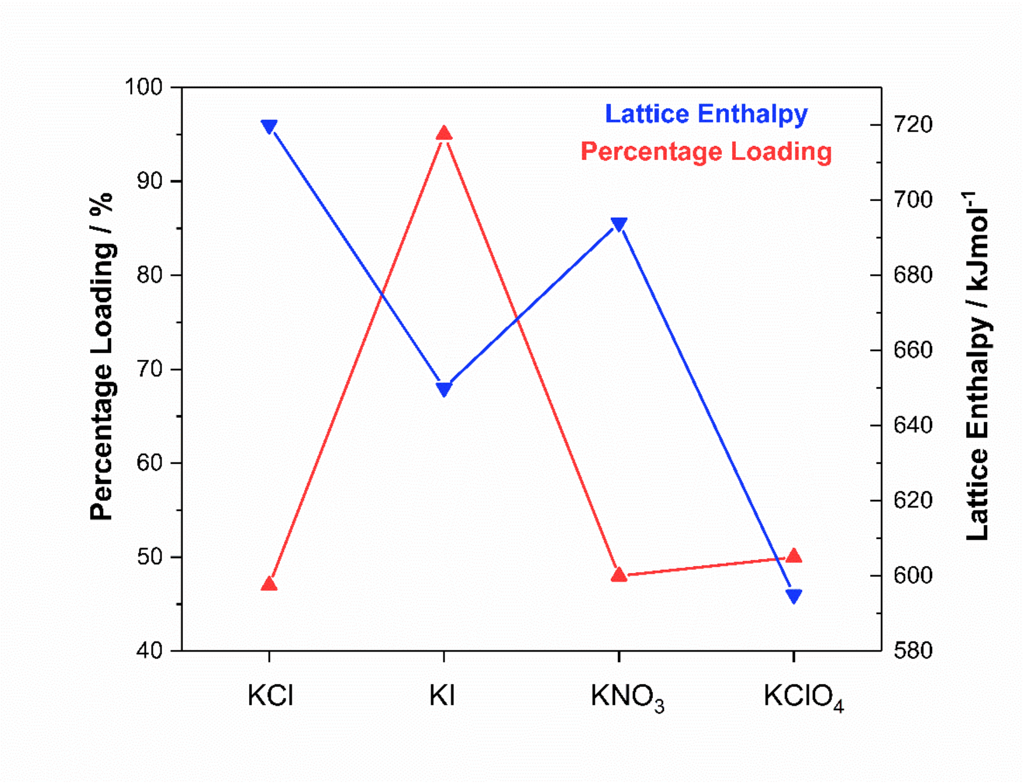

Figure S45. Plots showing calculated percentage loadings versus lattice enthalpies for **1·ChB**[PFP] SLE studies with various potassium salts.

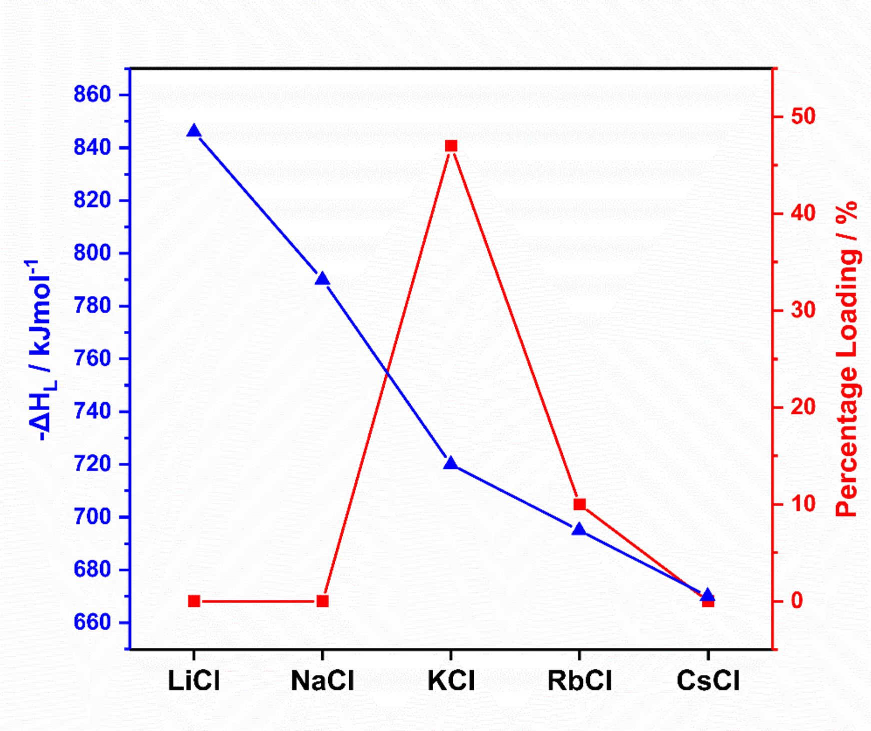

Figure S46. Plots showing calculated percentage loadings versus lattice enthalpies for **1·ChB**[PFP] SLE studies for various alkali metal chloride salts.

Liquid-Liquid Extraction (LLE) Procedure

In a typical experiment a $CDCl_3$ solution of the receptor (0.7 ml) was added to a D_2O solution of the alkali metal salt (0.7 ml), to which was added a stirrer bar and the mixture was stirred vigorously for 30 mins. After which time the biphasic mixture was allowed to settle, the organic phase removed and the ^1H NMR spectrum recorded.

Table S9. Summarised liquid-liquid extraction efficiencies for **1•ChB^{PFP}** with various KCl concentrations in D_2O. [**1•ChB^{PFP}**] = 4 mM.

[KCl]	1 M	2 M	3 M	4 M	Sat.
Extraction[a]	32%	43%	64%	69%	72%

[a] Extraction as determined by integration of ^1H NMR spectrum resonances of the internal aromatic signals corresponding to free and complexed receptor.

Table S10. Summarised liquid-liquid extraction efficiencies for **1•ChB^{PFP}** with various receptor concentrations in $CDCl_3$. [KCl] = 4 M.

[**1•ChB^{PFP}**]	1 mM	2 mM	4 mM	8 mM
Extraction[a]	66%	66%	69%	75%

[a] Extraction as determined by integration of ^1H NMR spectrum resonances of the internal aromatic signals corresponding to free and complexed receptor.

Table S11. Summarised liquid-liquid extraction efficiencies for **1•ChB^{PFP}** with various alkali metal chloride salts. [MCl] = 4 M. [**1•ChB^{PFP}**] = 4 mM.

Salt	LiCl	NaCl	KCl	RbCl	CsCl
Extraction[a]	0%	0%	69%	0%	0%

[a] Extraction as determined by integration of ^1H NMR spectrum resonances of the internal aromatic signals corresponding to free and complexed receptor.

Table S12. Summarised liquid-liquid extraction efficiencies for **1•ChB^{PFP}** with various potassium salts. [KX] = 4 M. [**1•ChB^{PFP}**] = 4 mM.

Salt	KNO_3	KBr	KI
Extraction[a]	-[b]	>95%	>95%

[a] Extraction as determined by integration of ^1H NMR spectrum resonances of the internal aromatic signals corresponding to free and complexed receptor. [b] Signals too broad to accurately determine extraction percentages.

Table S13. Summarised liquid-liquid extraction efficiencies for **1•ChB^Ph^** with various alkali metal chloride salts. [MCl] = 4 M. [**1•ChB**^{Ph}] = 4 mM.

Salt	LiCl	NaCl	KCl	RbCl	CsCl
Extraction[a]	0%	0%	0%	0%	0%

[a] Extraction as determined by integration of ^{1}H NMR spectrum resonances of the internal aromatic signals corresponding to free and complexed receptor.

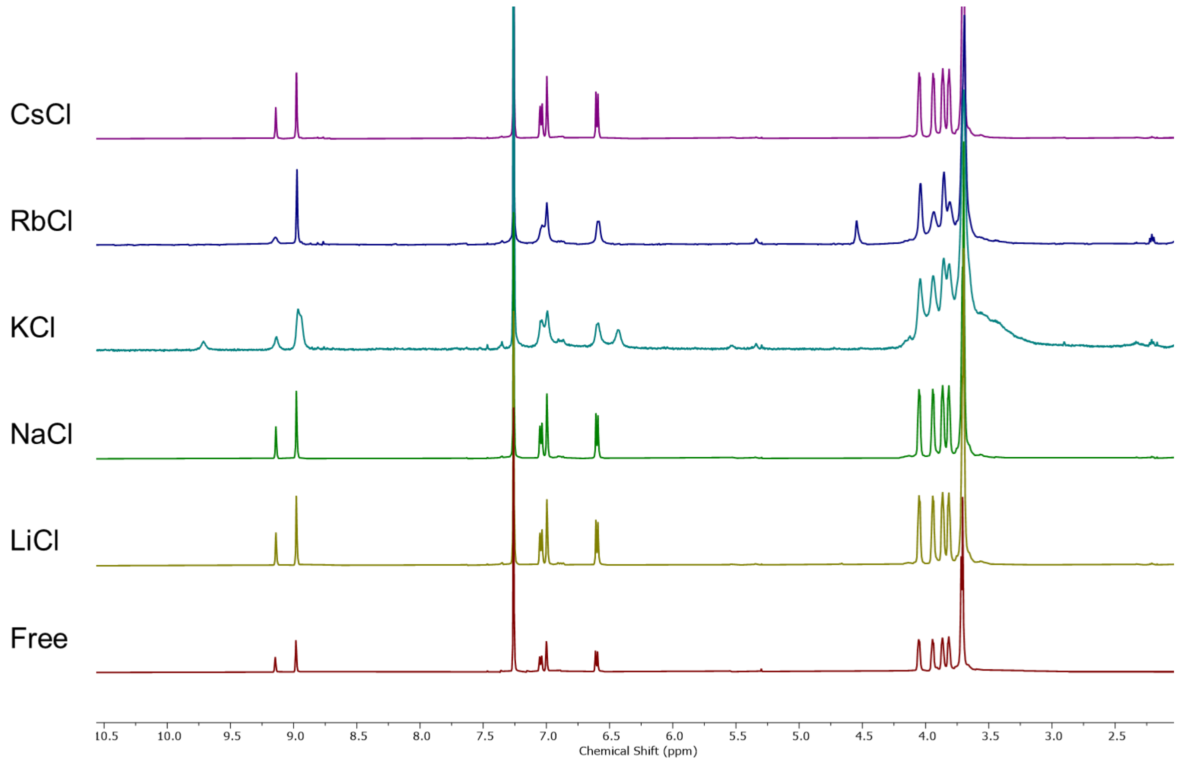

Figure S47. ^{1}H NMR spectrum from LLE extraction experiment of **1·ChB**^{PFP} ($CDCl_3$, 500 MHz, 298K).

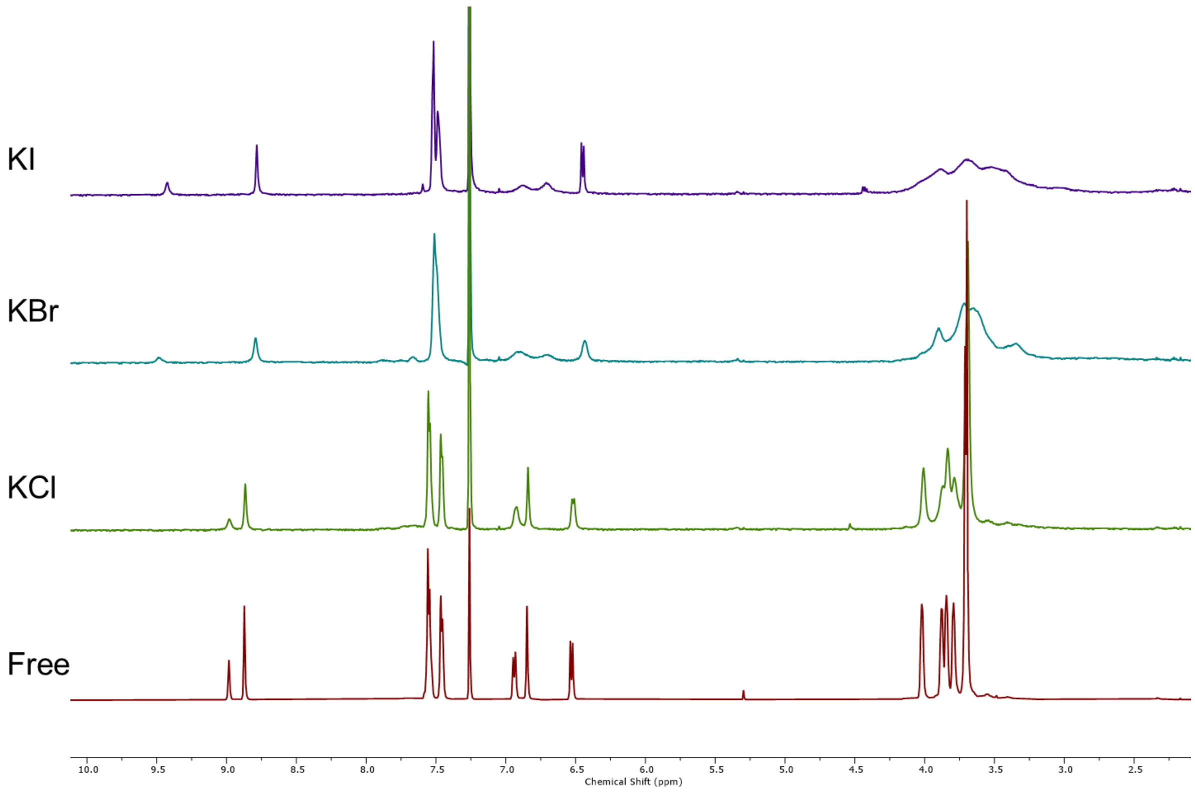

Figure S48. ^{1}H NMR spectrum from LLE extraction experiment of **1·ChB**^{Ph} ($CDCl_3$, 500 MHz, 298K).

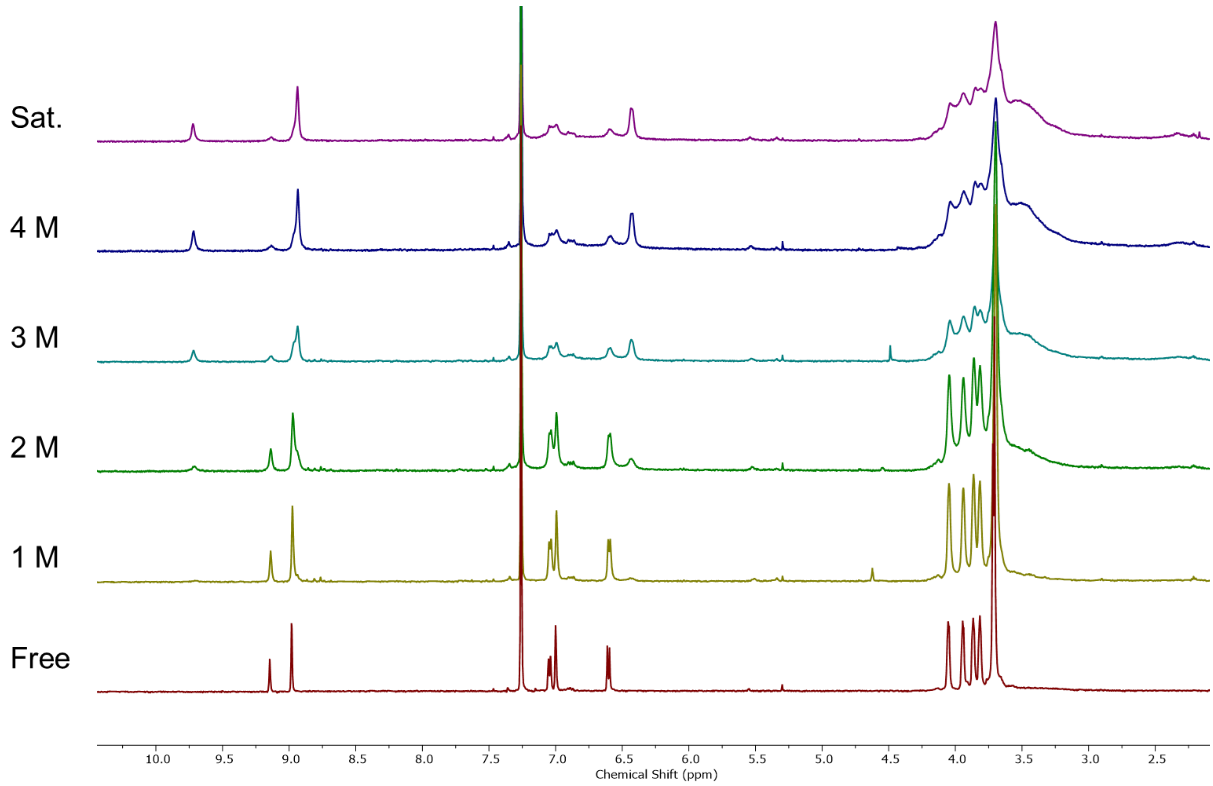

Figure S49. ^{1}H NMR spectrum from LLE extraction experiment of **1·ChB**^{PFP} with various concentrations of $KCl_{(aq)}$ ($CDCl_3$, 500 MHz, 298K).

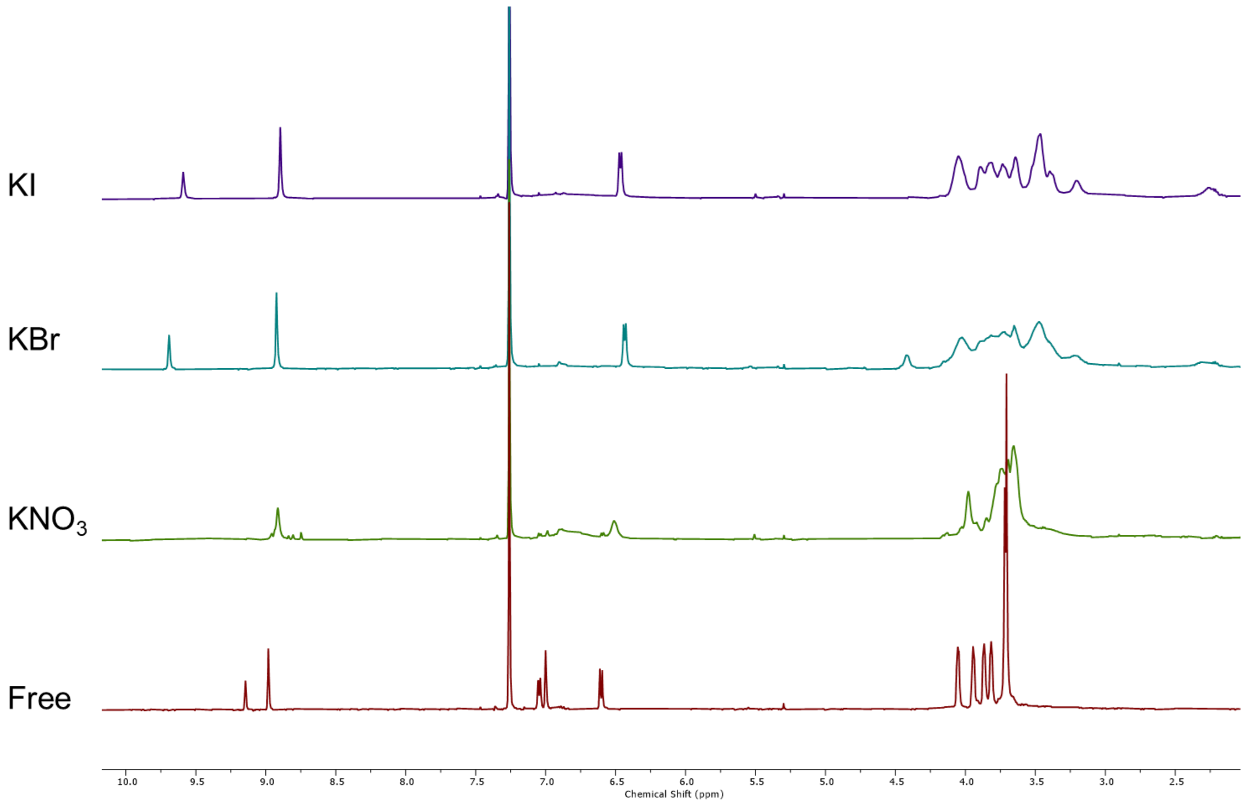

Figure S50. ^{1}H NMR spectrum from LLE extraction experiment of **1·ChB^{PFP}** ($CDCl_3$, 500 MHz, 298K).

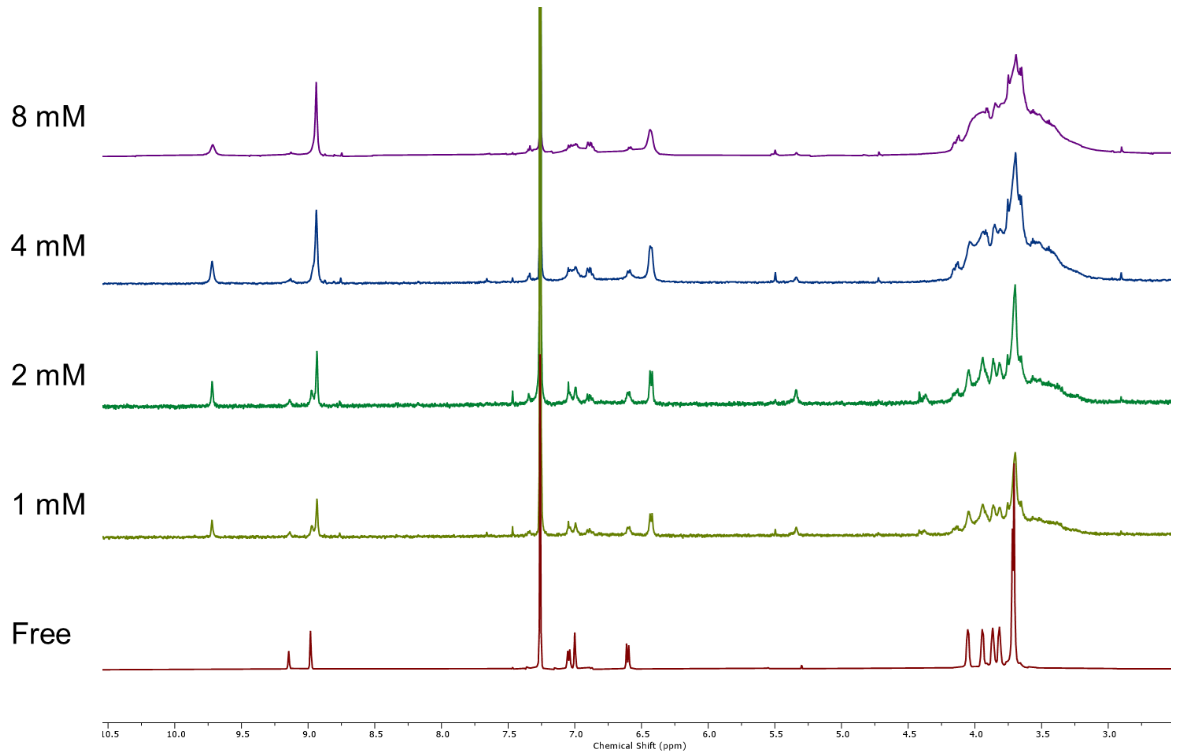

Figure S51. ^{1}H NMR spectrum from LLE extraction experiment with various **1·ChB^PFP^** concentrations ($CDCl_3$, 500 MHz, 298K).

Liquid Membrane Transport Studies

The chloride selective electrode (Thermo Scientific™ Orion™ ionplus® Sure-Flow® Solid State Combination ISE) was soaked in 1000 ppm chloride for extended periods (>1 hr) until readings were stable. Before each run, the electrode was calibrated by recording the potential of known chloride concentrations. The readings (mV) were converted to chloride activity using the modified Nernst equation (Equation S1), where x is chloride activity, y is potential (mV) and a and b are parameters to be determined and c=0.

Equation S1 : $y = a - \ln(x + c)$

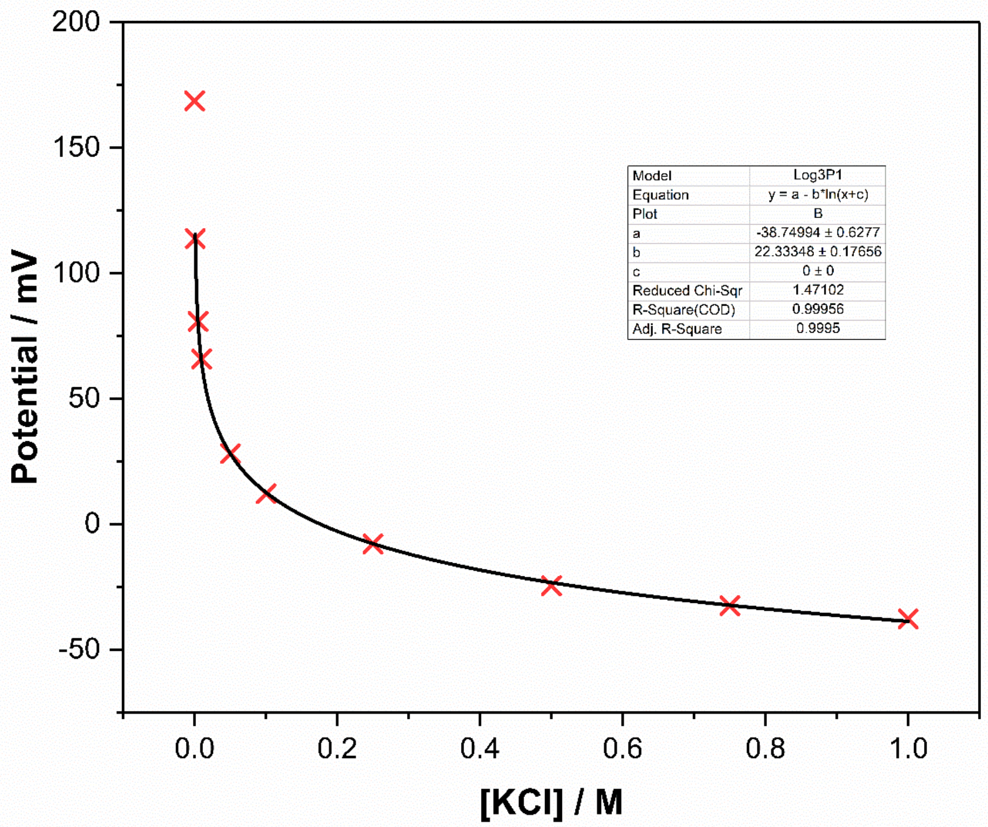

Figure S52. Chloride selective electrode calibration curve, experimental data indicated by red crosses and solid line indicating fitting.

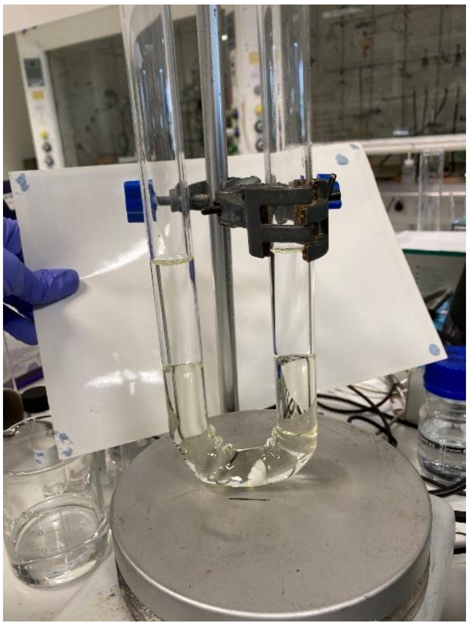

Figure S53. Photograph of the U-tube set up.

In general U-tube transport experiments were conducted as follows: A stirrer bar was placed in the bottom of the U-tube and 30 ml of an 8 mM $CHCl_3$ receptor solution was syringed into the U-tube. On the left hand side was added 15 ml of distilled water (receiving phase) and on the right hand side was added 15 ml 4 M $MCl_{(aq)}$ (source phase). The stirring speed was set at 600 rpm. The chloride selective electrode was immersed in the receiving phase at 0, 1, 5, 12, 18, 24 and 36 hours after set up, and the potential recorded.

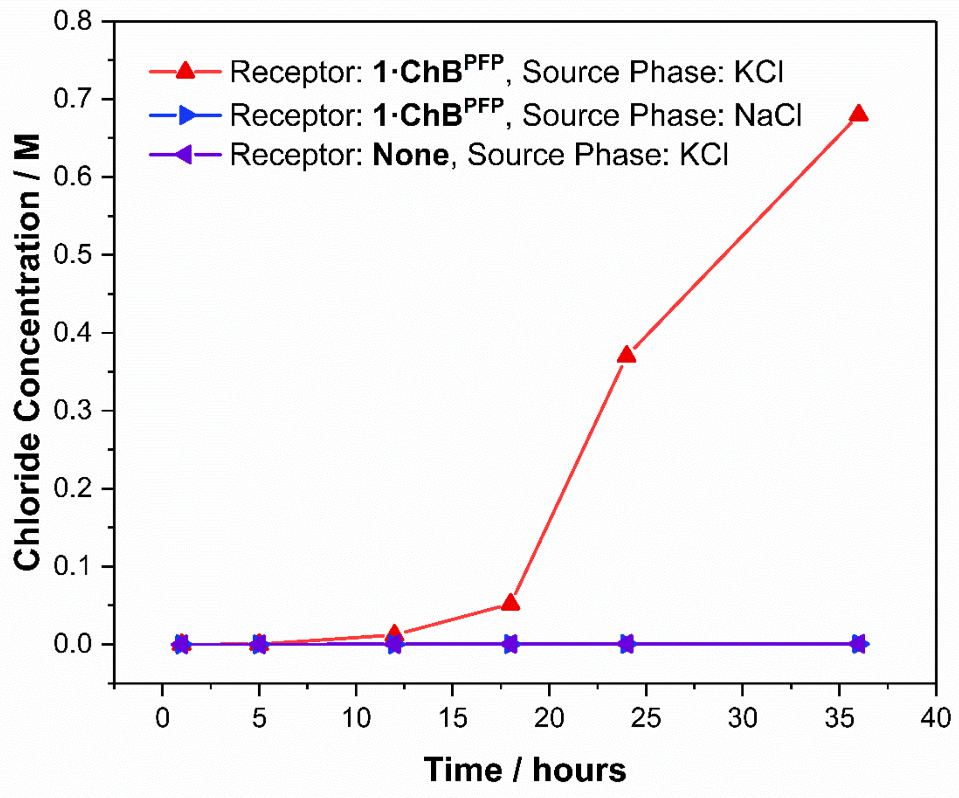

Figure S54. Potassium Chloride ion-pair liquid membrane from source phase to receiving phase, experimental data indicated by coloured triangles and solid lines as a visual aid.

Computational Studies

Detailed Methods

All DFT calculations were carried out with Gaussian 16 (Rev. C.01),[1] using the M06-2X[2] functional coupled with the Def2-TZVP basis set[3–5] in gas-phase or in chloroform or acetonitrile. Moreover, K^+**1·ChB**^{PFP} and its ion-pair complexes were also optimised in solution using the Def2-TZVPD[4–6] basis set on the Te centres and on the anions, as taken from the Basis Set Exchange database.[7–9] Both solvents were described with the Conductor-like Polarizable Continuum Model (C-PCM).[10,11] Tight SCF convergence criterium was used, together with an ultrafine grid for numerical integrations. Finally, each DFT optimised structure was ascertained to be a minimum, as no imaginary frequencies were found. The sole exception was the reoptimized structure of K^+**1·ChB**^{PFP}in acetonitrile with the augmented basis set, with a low imaginary frequency of 4.21i cm^{-1}.

The electrostatic potential distributions of **1·ChB**^{PFP} complexed with K^+, Rb^+ or Cs^+, or in its respective conformations in the absence of the alkali cation were mapped onto their electron density surfaces (V_S, estimated at 0.001 ea_0^{-3}) with MultiWFN 3.7.[12] The anions' V_S were calculated at the M06-2X/Def2-TZVPD theory level in gas-phase. The strength of the ChB and HB interactions between K^+**1·ChB**^{PFP} and the different anion guests was evaluated through the Natural Bond Orbital (NBO) analysis, using NBO 7.0[13,14] from the optimised structures with the Def2-TZVP(D) basis set. The strength of the interactions between the alkali cations and the crown-ether oxygen atoms was also evaluated through NBO analysis in the gas-phase, using the Def2-TZVP basis set for the M^+**1·ChB**^{PFP} complexes, and in both solvents, using the Def2-TZVP(D) basis set for the K^+**1·ChB**^{PFP} anion complexes.

The binding free energy (ΔG) of the different associations between the K^+ complexes of **1·ChB**^{PFP} and the anions was estimated through the following equation:

$$\Delta G = G_{complex} - G_{receptor} - G_{anion} \qquad \textbf{Eq. S2}$$

where $G_{complex}$, $G_{receptor}$ and G_{anion} are the free energies estimated from the ground state optimised structures of the ion-pair complexes, the cation sandwich heteroditopic receptor and the free anions, respectively. The free energy of each entity was obtained as $G = H - TS$, where H is the enthalpy, T is the absolute temperature (298.15 K), and S is the entropy. The enthalpy was obtained as $H = \mathcal{E}_0 + E_{tot} + RT$, where $\mathcal{E}_0$ is the total electronic energy, E_{tot} is the total internal energy, accounting the contributions from the translational, rotational, vibrational, and electronic motions, while RT is the thermal correction, R being the constant for ideal gases and T the temperature. Furthermore, E_{tot} already includes the zero-point vibrational energy correction (ZPE). S accounts for the entropy contributions from the translational, rotational, vibrational, and electronic motions. Finally, the ΔG is further corrected with the free energy change converting from the standard state at 1 atm (1 mol per 24.46 L) to 1 M (1 mol/L), -1.89 kcal mol^{-1}, to afford ΔG^{SS}, as $\Delta G^{SS} = \Delta G + (-1.89\ \text{kcal mol}^{-1})$.[15]

Detailed Discussion

The NBO analysis was also used to ascertain the strength of the ChB and HB interactions on the K^+**1·ChB**^{PFP} anion complexes in both solvents. Within this approach, the ChB interactions result from the interaction between the electron lone pairs of the anion guests (n_A) and the antibonding orbitals of the C_{trz}-Te bond ($\sigma^*_{C\text{-}Te}$), affording the E^2 energies listed in Table S21 for the two binding units. In agreement with the

pattern of ChB distances discussed in the main text, two distinct E^2 values were computed for each anion association, with the largest one corresponding to the shortest binding distance. Accordingly, the values assessed in acetonitrile are globally lower than those in chloroform. Noteworthy, the cooperative recognition of each anion by the two ChB interactions leads to overall E^2 values that linearly correlate with the ΔH binding values of the thermodynamically favoured ion-pair complexes (see Table S19 and Table S20), when the data are grouped by solvent (chloroform or acetonitrile), and anions' type (halide or oxyanions), as shown in Figure S60. Table S21 also includes the E^2 energies corresponding to the HB interaction between the ancillary C_{Ar}-H bond and the anion guest (*i.e.*, the $n_A \rightarrow \sigma^*_{C\text{-}H}$ interaction). These stabilisation energies are typically larger for the halide guests, contributing with *ca.* 20% to the overall anion recognition, while for the oxyanions this contribution drops to *ca.* 10%, regardless of the solvent or cationic sandwiched receptor. Overall, the total E^2 values of the M···O bonds for the K^+**1·ChB**^{PFP} ion-pair complexes appear to be independent of the solvent nature and are like those computed in the K^+**1·ChB**^{PFP} receptor (Table S21).

Supplementary Figures

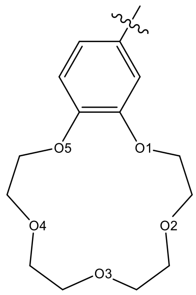

Figure S55. Labelling scheme used in Table S14 and Table S15 for the oxygen atoms of the two crown-ether moieties of **1·ChB**^{PFP}.

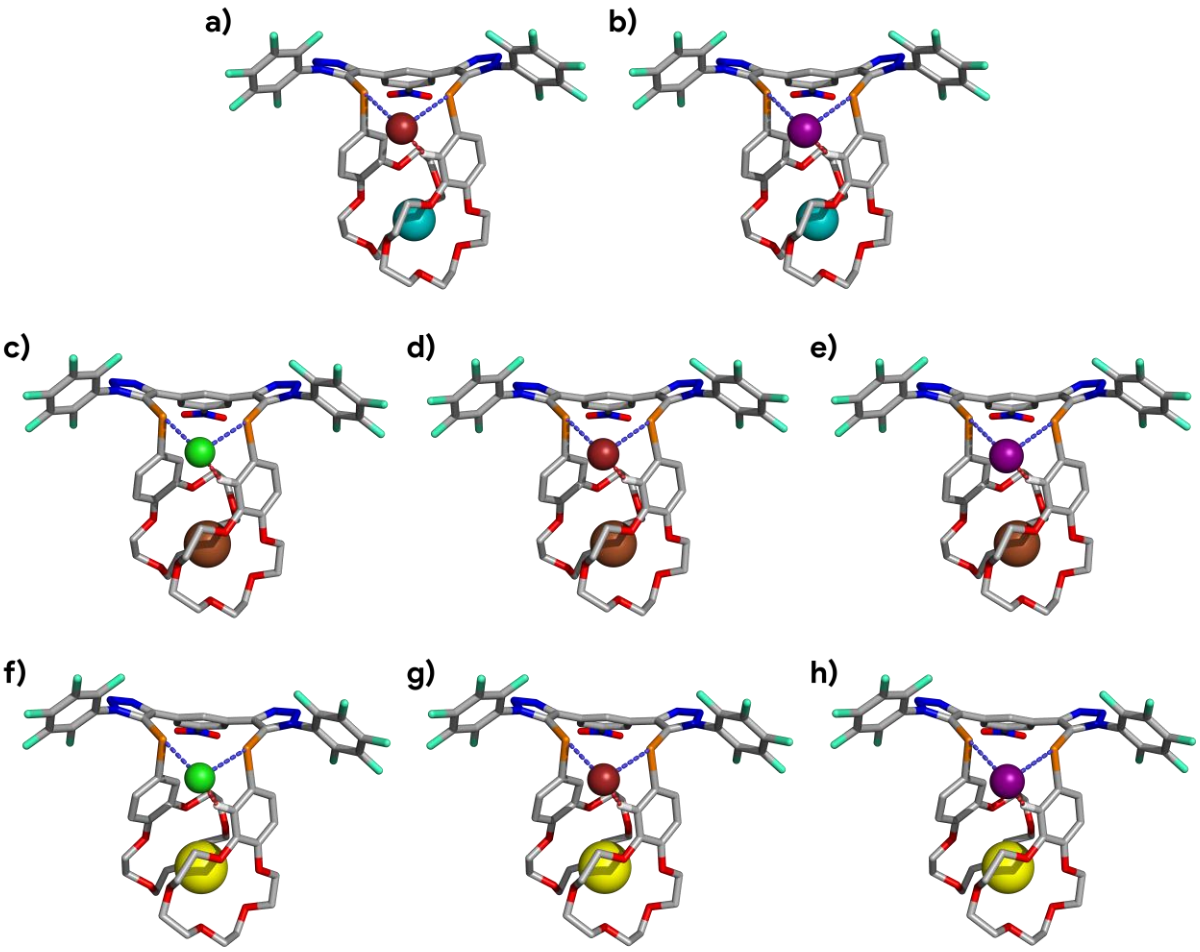

Figure S56. DFT optimised structures of **1·ChB^{PFP}** complexes with KBr (a), KI (b), RbCl (c), RbBr (d), RbI (e), CsCl (f), CsBr (g), and CsI (h) ion-pairs in chloroform. The ChB interactions are drawn in light blue dashes, while the HB interactions are drawn in red. Apart from the donor C_{Ar}-H proton, all hydrogen atoms were hidden for clarity.

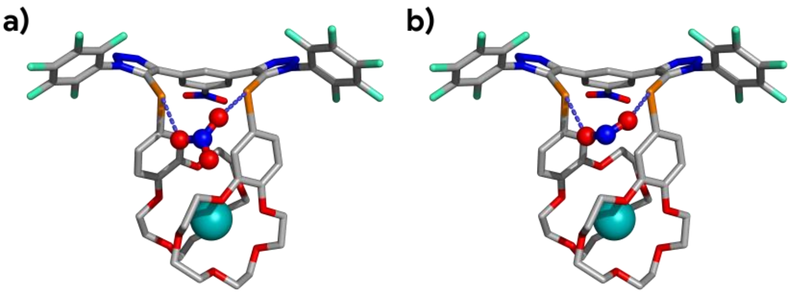

Figure S57. DFT optimised structures of **1·ChB^{PFP}** complexes with KNO_3 (a) and KNO_2 (b) ion-pairs in chloroform. The ChB interactions are drawn in light blue dashes. Hydrogen atoms were hidden for clarity.

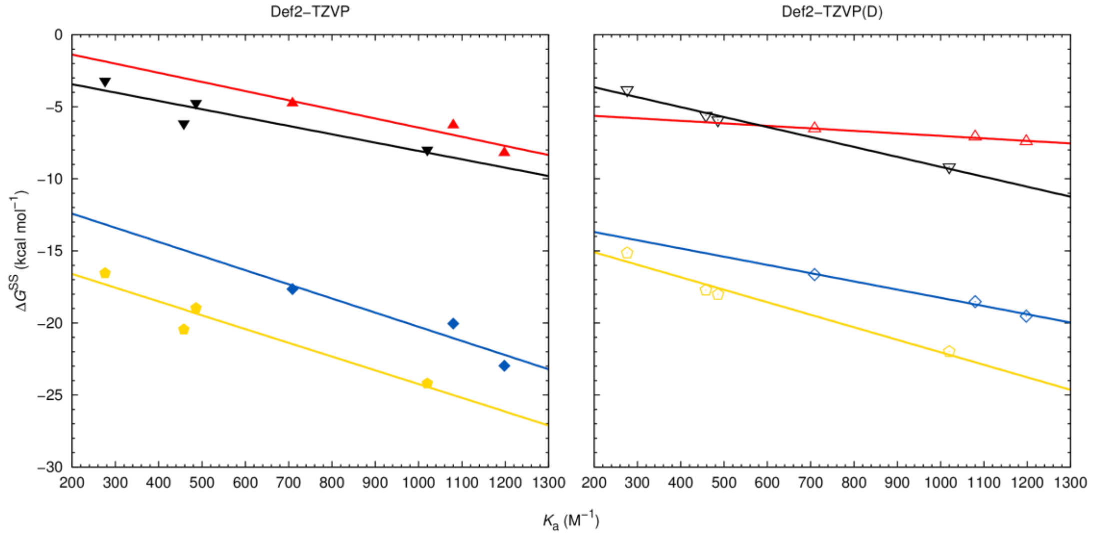

Figure S58. Representation of K^+**1·ChB**^{PFP} ΔG^{SS} (kcal mol^{-1}) with the different anions as a function of the experimental K_a (M^{-1}) for the two different basis sets (Def2-TZVP and Def2-TZVP(D)), with halides in $CHCl_3$ (◆ or ◇), oxoanions in $CHCl_3$ (⬟ or ⬠), halides in CH_3CN (▲ or △), and oxoanions in CH_3CN (▼ or ▽), together with the corresponding linear fits of R^2 = 0.88, 0.99, 0.91, 0.97, 0.88, 0.98, 0.83 and 0.98 in this order (values in blue for the Def2-TZVP(D) basis set).

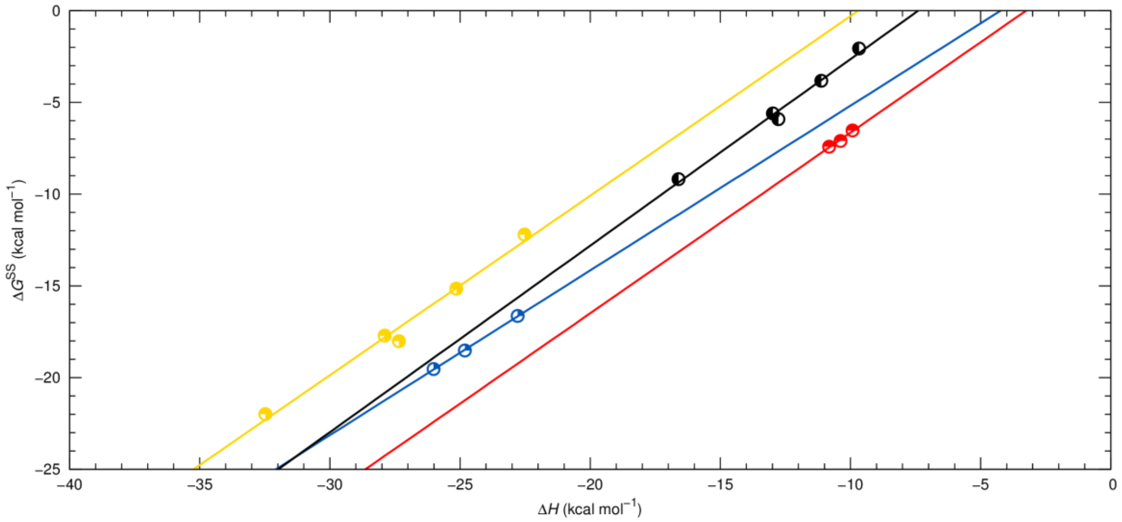

Figure S59. Representation of ΔG^{SS} (kcal mol^{-1}) as a function of ΔH (kcal mol^{-1}) for the interaction of the K^+ sandwiched receptor **1·ChB**^{PFP} with halides in $CHCl_3$ (◔), oxoanions in $CHCl_3$ (◔), halides in CH_3CN (◒), and oxoanions in CH_3CN (◐), together with the corresponding linear fits of R^2 = 1.00, 0.98, 0.98, and 0.99, in this order.

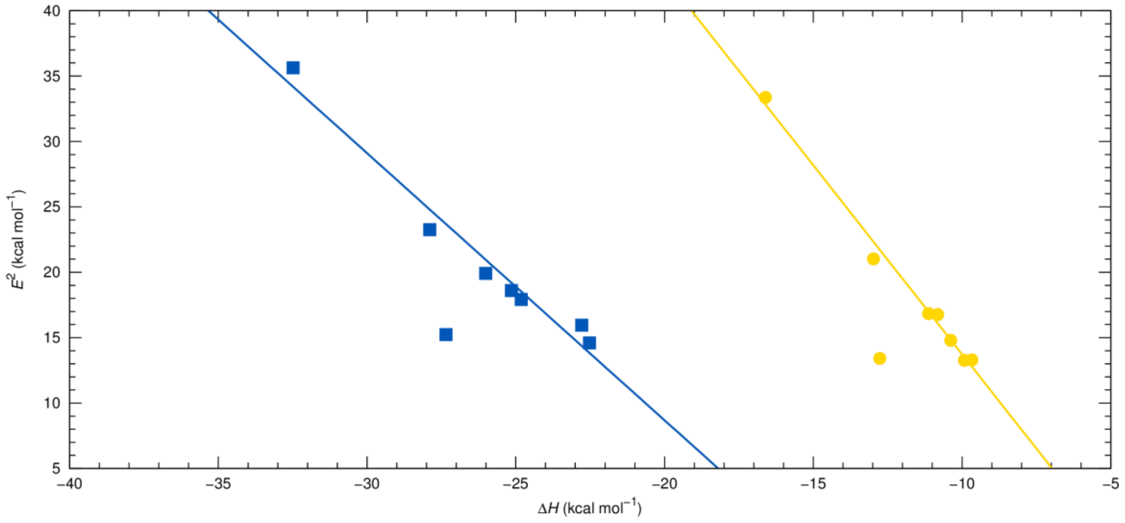

Figure S60. Overall E^2 stabilisation energy (kcal mol^{-1}) of the ChB interactions with anion guests, as a function of ΔH (kcal mol^{-1}) between K^+**1·ChB**$^{\mathbf{PFP}}$ and the halides and oxoanions in $CHCl_3$ (■) or in CH_3CN (●), together with the corresponding linear fits excluding OCN^- (R^2 = 0.97 and 0.99).

Supplementary Tables

Table S14. M···O distances (Å) assessed in the DFT optimised structures of sandwiched alkali cation complexes of **1·ChB**^{PFP} in the gas-phase, together with the corresponding E^2 energy values (kcal mol^{-1}). See Figure S55 for identification of the oxygen atoms in each crown-ether (a or b).

**1·ChB**^{PFP} complex	K^+		Rb^+		Cs^+	
Oxygen atoms	M···O	E^2	M···O	E^2	M···O	E^2
O1(a)	2.763	3.35	2.924	2.88	3.117	2.00
O2(a)	2.791	3.99	2.889	3.99	3.075	3.16
O3(a)	2.731	4.15	2.867	4.18	3.082	3.20
O4(a)	2.847	3.66	2.949	3.84	3.095	3.07
O5(a)	2.896	3.08	3.000	2.81	3.170	2.01
O1(b)	2.815	3.07	3.002	2.66	3.233	1.83
O2(b)	2.806	3.99	2.895	3.98	3.073	3.17
O3(b)	2.743	4.07	2.880	4.10	3.104	3.14
O4(b)	2.834	3.75	2.936	3.90	3.098	3.12
O5(b)	2.917	2.94	2.995	2.75	3.144	1.97
Average M···O	2.814	-	2.934	-	3.119	-
Range M···O	2.731; 2.917	-	2.867; 3.002	-	3.073; 3.233	-
Average E^2	-	3.61	-	3.51	-	2.67
Total E^2	-	36.05	-	35.09	-	26.67

Table S15. Gas-phase NPA charges (*e*) of the **1·ChB**^{PFP} heteroditopic receptor, complexed or in the absence of the sandwiched alkali cations,[a)] together with the net charge of the complexed cation.

Oxygen atoms	K^+**1·ChB**^{PFP}	**1·ChB**$^{PFP b}$	Rb^+**1·ChB**^{PFP}	**1·ChB**$^{PFP c}$	Cs^+**1·ChB**^{PFP}	**1·ChB**$^{PFP d}$
O1(a)	-0.499	-0.449	-0.491	-0.449	-0.488	-0.450
O2(a)	-0.544	-0.506	-0.546	-0.509	-0.542	-0.505
O3(a)	-0.540	-0.497	-0.538	-0.497	-0.537	-0.498
O4(a)	-0.533	-0.497	-0.534	-0.497	-0.535	-0.497
O5(a)	-0.481	-0.439	-0.481	-0.442	-0.480	-0.444
O1(b)	-0.490	-0.448	-0.486	-0.450	-0.482	-0.452
O2(b)	-0.539	-0.502	-0.541	-0.505	-0.540	-0.504
O3(b)	-0.538	-0.496	-0.535	-0.495	-0.534	-0.496
O4(b)	-0.533	-0.495	-0.533	-0.495	-0.534	-0.496
O5(b)	-0.483	-0.445	-0.484	-0.448	-0.483	-0.450
Average O	-0.518	-0.477	-0.517	-0.479	-0.516	-0.479
Te1	0.611	0.583	0.612	0.583	0.611	0.582
Te2	0.582	0.556	0.581	0.556	0.580	0.556
Net cation charge	0.859	-	0.869	-	0.884	-

[a)] The slight differences on the NPA charges for **1·ChB**^{PFP} are due to the small t structural distortions on the heteroditopic receptor imposed by the complexation of alkali cations with different ion sizes; [b)] in the geometry present in the K^+**1·ChB**^{PFP} complex; [c)] in the geometry present in the Rb^+**1·ChB**^{PFP} complex; and [d)] in the geometry present in the Cs^+**1·ChB**^{PFP} complex.

Table S16. Dimensions of the ChB and HB interactions in the halide associations of the sandwiched cation complexes of **1·ChB^{PFP}**, together with the alkali···halide distances and the range of distances between the cation and crown-ether oxygens atoms (distances in Å and angles in °). Values given in black are for the Def2-TZVP basis set, while the values in blue are for the Def2-TZVP(D) basis set.

Solvent	Chloroform									Acetonitrile				
Ion-pair	KCl	KBr	KI	RbCl	RbBr	RbI	CsCl	CsBr	CsI	KCl	KBr	KI	RbCl	CsCl
Te1···X	3.363	3.560	3.812	3.389	3.595	3.837	3.421	3.611	3.848	3.391	3.616	3.852	3.424	3.472
	3.379	3.570	3.812	–	–	–	–	–	–	3.430	3.628	3.853	–	–
Te2···X	3.137	3.331	3.571	3.121	3.309	3.553	3.098	3.299	3.541	3.198	3.395	3.635	3.185	3.159
	3.157	3.342	3.571	–	–	–	–	–	–	3.217	3.408	3.639	–	–
C_{trz}-Te1···X	166.0	167.4	168.8	166.4	167.7	169.3	166.9	168.6	170.2	166.7	167.8	169.3	167.2	167.5
	166.2	167.5	168.8	–	–	–	–	–	–	166.7	167.9	169.4	–	–
C_{trz}-Te2···X	166.6	168.3	170.4	167.6	169.4	171.8	168.4	170.4	172.8	168.0	169.8	172.1	168.9	169.9
	166.9	168.5	170.4	–	–	–	–	–	–	168.3	170.1	172.2	–	–
H···X	2.462	2.625	2.841	2.441	2.612	2.832	2.446	2.616	2.842	2.515	2.683	2.897	2.492	2.498
	2.477	2.635	2.841	–	–	–	–	–	–	2.535	2.695	2.900	–	–
C_{Ar}-H···X	149.2	150.2	150.6	150.6	151.6	151.2	149.9	149.4	148.1	147.9	149.6	149.0	149.4	148.8
	149.2	150.2	150.6	–	–	–	–	–	–	148.7	149.5	148.9	–	–
M···X	7.162	7.283	7.451	7.135	7.291	7.481	7.186	7.357	7.569	7.248	7.372	7.544	7.227	7.259
	7.177	7.291	7.451	–	–	–	–	–	–	7.260	7.383	7.548	–	–
M···O [range]	2.742; 2.942	2.740; 2.939	2.738; 2.934	2.885; 3.016	2.885; 3.015	2.884; 3.016	3.057; 3.17	3.058; 3.173	3.060; 3.171	2.742; 2.942	2.729; 2.942	2.728; 2.938	2.878; 3.02	3.057; 3.183
	2.741; 2.94	2.740; 2.938	2.738; 2.934	–	–	–	–	–	–	2.730; 2.947	2.730; 2.941	2.728; 2.937	–	–

Table S17. Comparison between the DFT computed structures for ion-pair complexes of **1·ChB**[PFP] in chloroform and the corresponding X-ray single crystal strcutures with distances in ångströms (Å) and angles in degrees (°).[a]

Ion-pair	KCl[b]	KBr[b]	KI	RbI	CsI
Te1···X	3.363 (3.418)	3.560 (3.522)	3.812 (3.889)	3.837 (3.871)	3.848 (3.904)
Te2···X	3.137 (3.117)	3.331 (3.252)	3.571 (3.460)	3.553 (3.466)	3.541 (3.478)
C_{trz}-Te1···X	166.0 (165.2)	167.4 (165.8)	168.8 (166.8)	169.3 (167.7)	170.2 (168.8)
C_{trz}-Te2···X	166.6 (170.7)	168.3 (171.4)	170.4 (173.5)	171.8 (173.6)	172.8 (174.3)
H···X	2.462 (2.645)	2.625 (2.751)	2.841 (3.012)	2.832 (3.016)	2.842 (3.005)
C_{Ar}-H···X	149.2 (151.8)	150.2 (152.6)	150.6 (159.5)	151.2 (158.4)	148.1 (160.5)
M···X	7.162 (7.267)	7.283 (7.314)	7.451 (7.475)	7.481 (7.462)	7.569 (7.444)
M···O [range]	2.742; 2.942 (2.737; 3.105)	2.740; 2.939 (2.732; 3.101)	2.738; 2.934 (2.747; 2.984)	2.884; 3.016 (2.840; 3.048)	3.060; 3.171 (3.015; 3.183)

[a)] Values in brackets are for single crystal X-ray structures; [b)] The experimental values listed for KCl and KBr ion-pair complexes are the average calculated with the two molecules of the asymmetric unit.

Table S18. Dimensions of the ChB and HB interactions in the DFT optimised structures of the K^{+}**1·ChB**^{PFP} oxoanion complexes, together with range of distances between the cation and crown-ether oxygens atoms (distances in Å and angles in °). Values given in black are for the Def2-TZVP basis set, while the values in blue are for the Def2-TZVP(D) basis set.

Solvent			Chloroform					Acetonitrile		
Ion-pair	KAco	KNO_3	KNO_2	KOCN	$KClO_4$	KAco	KNO_3	KNO_2	KOCN	$KClO_4$
Te1···O	2.644	2.896	2.797	2.877	2.976	2.668	2.912	2.821	2.900	3.015
	2.651	2.901	2.805	2.879	2.978	2.678	2.918	2.832	2.903	3.017
Te2···O[a]	2.678	2.844	2.795	2.809	2.920	2.699	2.880	2.825	2.869	2.943
	2.678	2.845	2.803	2.817	2.922	2.699	2.883	2.836	2.878	2.945
C_{trz}-Te1···O	173.7	176.9	174.3	175.1	171.2	174.0	178.1	174.5	175.2	171.6
	173.8	177.3	174.3	175.3	171.0	174.0	178.1	174.4	175.3	171.4
C_{trz}-Te2···O[a]	167.2	164.5	166.1	169.3	164.1	168.3	165.9	167.4	170.8	165.6
	167.3	164.6	166.3	169.4	164.2	168.4	166.1	167.6	170.9	165.8
H···O[a]	2.250	2.339	2.258	2.610	2.308	2.279	2.355	2.284	2.657	2.346
	2.256	2.336	2.264	2.609	2.317	2.276	2.343	2.291	2.657	2.354
C_{Ar}-H···O[a]	168.3	166.4	168.8	166.8	171.1	167.0	164.8	167.2	165.8	170.0
	168.6	166.6	168.8	167.0	170.9	167.3	165.0	167.1	166.0	169.8
M···O [range]	2.739; 2.939	2.736; 2.938	2.738; 2.935	2.738; 2.922	2.737; 2.918	2.729; 2.948	2.725; 2.946	2.727; 2.941	2.728; 2.932	2.728; 2.926
	2.739; 2.938	2.737; 2.938	2.738; 2.935	2.737; 2.923	2.736; 2.918	2.729; 2.948	2.725; 2.946	2.727; 2.941	2.728; 2.933	2.728; 2.926

[a] Distances and angles to the N atom of OCN^{-}.

Table S19. Uncorrected electronic binding energies ($\Delta\mathcal{E}_0$),[a] Zero Point Corrections (ΔZPE),[b] thermal corrected binding energies (ΔE),[c] binding enthalpies (ΔH),[d] binding entropies term contribution ($T\Delta S$),[e] binding free energies (ΔG),[f] and standard state binding free energies (ΔG^{SS})[g] (in kcal mol^{-1}) for the halide ion-pair associations of K$^+$**1·ChB^{PFP}** in chloroform and in acetonitrile.[h] Values given in black are for the Def2-TZVP basis set, while the values in blue are for the Def2-TZVP(D) basis set.

Energy term	Chloroform			Acetonitrile		
	KCl	KBr	KI	KCl	KBr	KI
$\Delta\mathcal{E}_0$	-29.45	-26.55	-23.97	-14.34	-12.47	-10.94
	-26.20	-24.99	-22.91	-11.63	-11.22	-10.74
ΔZPE	0.26	0.15	0.07	0.19	0.14	0.07
	0.30	0.16	0.04	0.28	0.20	0.13
ΔE	-28.70	-25.79	-23.24	-13.60	-11.66	-10.16
	-25.42	-24.22	-22.19	-10.23	-9.79	-9.33
ΔH	-29.30	-26.39	-23.83	-14.19	-12.26	-10.75
	-26.02	-24.81	-22.79	-10.82	-10.38	-9.92
$T\Delta S$	-8.21	-8.23	-8.07	-7.87	-7.86	-7.87
	-8.37	-8.18	-8.03	-5.30	-5.17	-5.28
ΔG	-21.08	-18.16	-15.76	-6.32	-4.40	-2.88
	-17.64	-16.63	-14.75	-5.52	-5.21	-4.64
ΔG^{SS}	-22.97	-20.05	-17.65	-8.21	-6.29	-4.77
	-19.53	-18.52	-16.64	-7.41	-7.10	-6.53

[a)] The energy values were not corrected for basis set superposition errors; [b)] ΔZPE is included in the ΔE, ΔH and ΔG terms; [c)] $\Delta E = \Delta\mathcal{E}_0 + \Delta E_{Tot}$, where ΔE_{Tot} accounts for the differences in the internal energy due to translational, rotational, vibrational and electronic motions; [d)] $\Delta H = \Delta E + \Delta nRT$, where n is -1 for a 1:1 host-guest systems, R is the ideal gas constant and T is the temperature (298.15 K); [e)] ΔS accounts for the differences in entropy due to in the translational, rotational, vibrational and electronic motions; [f)] $\Delta G = \Delta H - T\Delta S$; [g)] $\Delta G^{SS} = \Delta G + (-1.89$ kcal mol^{-1}), which corresponds to the free energy change converting from the standard state at 1 atm (1 mol per 24.46 L) to 1 M (1 mol/L);[15] [h)] the absolute energy terms are defined in the Detailed Methods section.

Table S20. Uncorrected electronic binding energies ($\Delta\mathcal{E}_0$),[a] Zero Point Corrections (ΔZPE),[b] thermal corrected binding energies (ΔE),[c] binding enthalpies (ΔH),[d] binding entropies term contribution ($T\Delta S$),[e] binding free energies (ΔG),[f] and standard state binding free energies (ΔG^{SS})[g] (in kcal mol^{-1}) for the oxoanion ion-pair associations of **1·ChB**$^{\mathbf{PFP}}$ in chloroform and in acetonitrile.[h] Values given in black are for the Def2-TZVP basis set, while the values in blue are for the Def2-TZVP(D) basis set.

Energy term	Chloroform					Acetonitrile				
	KAco	KNO_3	KNO_2	KOCN	$KClO_4$	KAco	KNO_3	KNO_2	KOCN	$KClO_4$
$\Delta\mathcal{E}_0$	-35.58	-27.98	-31.99	-29.17	-24.49	-20.21	-14.45	-17.43	-15.13	-12.21
	-33.82	-26.45	-29.16	-28.26	-23.78	-18.51	-13.07	-14.81	-14.33	-11.56
ΔZPE	0.97	0.90	1.09	0.83	0.68	0.96	0.85	0.95	0.81	0.65
	1.02	0.89	1.09	0.81	0.72	0.94	0.91	1.00	0.86	0.74
ΔE	-33.68	-26.10	-30.15	-27.66	-22.69	-18.33	-12.54	-15.64	-13.60	-10.38
	-31.89	-24.55	-27.30	-26.75	-21.93	-16.01	-10.53	-12.39	-12.18	-9.08
ΔH	-34.27	-26.69	-30.74	-28.25	-23.28	-18.93	-13.13	-16.24	-14.19	-10.97
	-32.48	-25.15	-27.89	-27.34	-22.52	-16.61	-11.12	-12.98	-12.77	-9.67
$T\Delta S$	-11.96	-12.04	-12.17	-11.17	-12.07	-12.84	-11.83	-11.96	-11.34	-12.05
	-12.38	-11.89	-12.07	-11.22	-12.21	-9.32	-9.19	-9.26	-8.74	-9.50
ΔG	-22.31	-14.65	-18.57	-17.08	-11.20	-6.09	-1.31	-4.27	-2.86	1.07
	-20.10	-13.26	-15.83	-16.13	-10.31	-7.29	-1.93	-3.72	-4.03	-0.17
ΔG^{SS}	-24.20	-16.54	-20.46	-18.97	-13.09	-7.98	-3.20	-6.16	-4.75	-0.82
	-21.99	-15.15	-17.72	-18.02	-12.20	-9.18	-3.82	-5.61	-5.92	-2.06

[a)] The energy values were not corrected for basis set superposition errors; [b)] ΔZPE is included in the ΔE, ΔH and ΔG terms; [c)] $\Delta E = \Delta\mathcal{E}_0 + \Delta E_{Tot}$, where ΔE_{Tot} accounts for the differences in the internal energy due to translational, rotational, vibrational and electronic motions; [d)] $\Delta H = \Delta E + \Delta nRT$, where n is -1 for a 1:1 host-guest systems, R is the ideal gas constant and T is the temperature (298.15 K); [e)] ΔS accounts for the differences in entropy due to in the translational, rotational, vibrational and electronic motions; [f)] $\Delta G = \Delta H - T\Delta S$; [g)] $\Delta G^{SS} = \Delta G + (-1.89\ \text{kcal mol}^{-1})$, which corresponds to the free energy change converting from the standard state at 1 atm (1 mol per 24.46 L) to 1 M (1 mol/L);[15] [h)] the absolute energy terms are defined in the Detailed Methods section.

Table S21. E^2 stabilisation energy values for the ChB and HB interactions between the K^+**1·ChB**$^{\mathbf{PFP}}$ sandwich host and the guest anions (A), together with overall E^2 energies for the interaction between the cation and the crown-ether oxygen atoms (kcal mol^{-1}), assessed at the M06-2X/Def2-TZVP(D) theory level.

Solvent	Chloroform									Acetonitrile								
Interaction	K^+	KCl	KBr	KI	KAco	KNO_3	KNO_2	KOCN	$KClO_4$	K^+	KCl	KBr	KI	KAco	KNO_3	KNO_2	KOCN	$KClO_4$
C_{trz}-Te1···A	–	7.17	6.38	5.52	18.70	7.89	11.26	7.97	6.61	–	6.11	5.30	4.78	17.32	7.53	10.36	7.31	5.87
C_{trz}-Te2···A^a	–	12.74	11.53	10.43	16.93	10.71	11.99	7.26	7.98	–	10.65	9.50	8.49	16.05	9.31	10.66	6.10	7.42
C_{Ar}-H···A^a	–	5.70	5.31	4.71	2.94	2.08	4.14	0.07	3.15	–	4.55	4.17	3.65	2.63	2.12	3.67	0.05	2.65
M···O (Total)	35.55	35.32	35.40	35.47	35.11	35.08	35.07	35.43	35.52	35.41	35.39	35.39	35.46	35.11	35.09	35.14	35.51	35.55

[a)] Interactions with the N atom of OCN^-.

References for Computational Studies

(1) Gaussian 16, Revision C.01, Frisch, M. J.; Trucks, G. W.; Schlegel, H. B.; Scuseria, G. E.; Robb, M. A.; Cheeseman, J. R.; Scalmani, G.; Barone, V.; Petersson, G. A.; Nakatsuji, H.; Li, X.; Caricato, M.; Marenich, A. V.; Bloino, J.; Janesko, B. G.; Gomperts, R.; Mennucci, B.; Hratchian, H. P.; Ortiz, J. V.; Izmaylov, A. F.; Sonnenberg, J. L.; Williams-Young, D.; Ding, F.; Lipparini, F.; Egidi, F.; Goings, J.; Peng, B.; Petrone, A.; Henderson, T.; Ranasinghe, D.; Zakrzewski, V. G.; Gao, J.; Rega, N.; Zheng, G.; Liang, W.; Hada, M.; Ehara, M.; Toyota, K.; Fukuda, R.; Hasegawa, J.; Ishida, M.; Nakajima, T.; Honda, Y.; Kitao, O.; Nakai, H.; Vreven, T.; Throssell, K.; Montgomery, J. A., Jr.; Peralta, J. E.; Ogliaro, F.; Bearpark, M. J.; Heyd, J. J.; Brothers, E. N.; Kudin, K. N.; Staroverov, V. N.; Keith, T. A.; Kobayashi, R.; Normand, J.; Raghavachari, K.; Rendell, A. P.; Burant, J. C.; Iyengar, S. S.; Tomasi, J.; Cossi, M.; Millam, J. M.; Klene, M.; Adamo, C.; Cammi, R.; Ochterski, J. W.; Martin, R. L.; Morokuma, K.; Farkas, Ö.; Foresman, J. B.; and Fox, D. J., Gaussian, Inc., Wallingford CT, 2019.

(2) Zhao, Y.; Truhlar, D. G. The M06 Suite of Density Functionals for Main Group Thermochemistry, Thermochemical Kinetics, Noncovalent Interactions, Excited States, and Transition Elements: Two New Functionals and Systematic Testing of Four M06-Class Functionals and 12 Other Functionals. *Theor. Chem. Acc.* **2008**, *120* (1–3), 215–241.

(3) Leininger, T.; Nicklass, A.; Küchle, W.; Stoll, H.; Dolg, M.; Bergner, A. The Accuracy of the Pseudopotential Approximation: Non-Frozen-Core Effects for Spectroscopic Constants of Alkali Fluorides XF (X = K, Rb, Cs). *Chem. Phys. Lett.* **1996**, *255* (4–6), 274–280.

(4) Peterson, K. A.; Figgen, D.; Goll, E.; Stoll, H.; Dolg, M. Systematically Convergent Basis Sets with Relativistic Pseudopotentials. II. Small-Core Pseudopotentials and Correlation Consistent Basis Sets for the Post- *d* Group 16–18 Elements. *J. Chem. Phys.* **2003**, *119* (21), 11113–11123.

(5) Weigend, F.; Ahlrichs, R. Balanced Basis Sets of Split Valence, Triple Zeta Valence and Quadruple Zeta Valence Quality for H to Rn: Design and Assessment of Accuracy. *Phys. Chem. Chem. Phys.* **2005**, 7 (18), 3297. https://doi.org/10.1039/b508541a.

(6) Rappoport, D.; Furche, F. Property-Optimized Gaussian Basis Sets for Molecular Response Calculations. *J. Chem. Phys.* **2010**, *133* (13), 134105.

(7) Pritchard, B. P.; Altarawy, D.; Didier, B.; Gibson, T. D.; Windus, T. L. New Basis Set Exchange: An Open, Up-to-Date Resource for the Molecular Sciences Community. *J. Chem. Inf. Model.* **2019**, *59* (11), 4814–4820.

(8) Feller, D. The Role of Databases in Support of Computational Chemistry Calculations. *J. Comput. Chem.* **1996**, *17* (13), 1571–1586.

(9) Schuchardt, K. L.; Didier, B. T.; Elsethagen, T.; Sun, L.; Gurumoorthi, V.; Chase, J.; Li, J.; Windus, T. L. Basis Set Exchange: A Community Database for Computational Sciences. *J. Chem. Inf. Model.* **2007**, *47* (3), 1045–1052.

(10) Barone, V.; Cossi, M. Quantum Calculation of Molecular Energies and Energy Gradients in Solution by a Conductor Solvent Model. *J. Phys. Chem. A* **1998**, *102* (11), 1995–2001.

(11) Cossi, M.; Rega, N.; Scalmani, G.; Barone, V. Energies, Structures, and Electronic Properties of Molecules in Solution with the C-PCM Solvation Model. *J. Comput. Chem.* **2003**, *24* (6), 669–681.

(12) Lu, T.; Chen, F. Multiwfn: A Multifunctional Wavefunction Analyzer. *J. Comput. Chem.* **2012**, *33* (5), 580–592.

(13) Glendening, E. D.; Landis, C. R.; Weinhold, F. Natural Bond Orbital Methods. *WIREs Comput. Mol. Sci.* **2012**, *2* (1), 1–42.

(14) Weinhold, F.; Landis, C. R.; Glendening, E. D. What Is NBO Analysis and How Is It Useful? *Int. Rev. Phys. Chem.* **2016**, *35* (3), 399–440.

(15) Kelly, C. P.; Cramer, C. J.; Truhlar, D. G. Single-Ion Solvation Free Energies and the Normal Hydrogen Electrode Potential in Methanol, Acetonitrile, and Dimethyl Sulfoxide. *J. Phys. Chem. B* **2007**, *111* (2), 408–422.

(16) Cosier, J.; Glazer, A. M. A Nitrogen-Gas-Stream Cryostat for General X-Ray Diffraction Studies. *J. Appl. Crystallogr.* **1986**, *19* (2), 105–107.

Crystal Structure Determination

Single crystals of **1·ChB**^{PFP}·KCl, **1·ChB**^{PFP}·KBr, **1·ChB**^{PFP}·KI, **1·ChB**^{PFP}·RbI, **1·ChB**^{PFP}·CsI, suitable for X-ray analysis were each coated with Paratone-N oil, suspended on a 200 μm MiTeGen loop, and placed in a cold gaseous nitrogen stream on an Oxford Diffraction Supernova X-ray diffractometer performing φ- and ω-scans at 150(2) K. Diffraction[16] intensities were measured using graphite monochromated Cu Kα radiation (1.54184 Å). Data collection, indexing, initial cell refinements, frame integration, final cell refinements and absorption corrections were accomplished using the program CrysAlisPro.[†] Scattering factors and anomalous dispersion corrections were taken from the International Tables for X-ray Crystallography. All structures were solved by direct methods and refined against F2. All hydrogen atoms were included into the model at geometrically calculated positions and refined using a riding model. Details of the data quality and a summary of the residual values for the refinements are listed in Tables S1 and S2. Figures of the crystal structures have been created using the open-source PyMOL Molecular Graphics System.

Deposition Number(s) 2155966 (for **1·ChB**^{PFP}·KCl) and 2155967 (for **1·ChB**^{PFP}·KBr) and 2155968 (for **1·ChB**^{PFP}·KI) and 2155964 (for **1·ChB**^{PFP}·RbI) and 2155963 (for **1·ChB**^{PFP}·CsI) contains the supplementary crystallographic data for this paper. These data are provided free of charge by the joint Cambridge Crystallographic Data Centre and Fachinformationszentrum Karlsruhe Access Structures service www.ccdc.cam.ac.uk/structures.

† CrysAlisPRO, Oxford Diffraction /Agilent Technologies UK Ltd, Yarnton, England.

Table S24. Crystal data and structure refinement for **1•ChB^{PFP}•KCl**.

Identification code	041hk20_sq	
Empirical formula	$C_{54.50}H_{45.50}Cl_{14.50}F_{10}KN_7O_{12}Te_2$	
Formula weight	1988.80	
Temperature	150(2) K	
Wavelength	1.54184 Å	
Crystal system	Triclinic	
Space group	P-1	
Unit cell dimensions	a = 17.0958(2) Å	α= 85.3110(10)°.
	b = 20.8045(4) Å	β= 89.2730(10)°.
	c = 25.0324(4) Å	γ = 68.248(2)°.
Volume	8240.2(3) Å^3	
Z	4	
Density (calculated)	1.603 Mg/m^3	
Absorption coefficient	11.051 mm^{-1}	
F(000)	3908	
Crystal size	0.4 x 0.4 x 0.4 mm^3	
Theta range for data collection	3.271 to 76.350°.	
Index ranges	-20<=h<=21, -26<=k<=25, -28<=l<=31	
Reflections collected	95933	
Independent reflections	33973 [R(int) = 0.0584]	
Completeness to theta = 67.684°	99.8 %	
Absorption correction	Sphere	
Max. and min. transmission	0.12587 and 0.03254	
Refinement method	Full-matrix least-squares on F^2	
Data / restraints / parameters	33973 / 0 / 1819	
Goodness-of-fit on F^2	1.026	
Final R indices [I>2sigma(I)]	R1 = 0.0725, wR2 = 0.1893	
R indices (all data)	R1 = 0.0827, wR2 = 0.2057	
Extinction coefficient	n/a	
Largest diff. peak and hole	2.661 and -1.077 e.Å^{-3}	

Table S25. Crystal data and structure refinement for **1•ChB**^{PFP}**•**KBr.

Identification code	036zy21_sq	
Empirical formula	$C_{54.50}H_{45.50}Br\,Cl_{13.50}F_{10}\,K\,N_7\,O_{12}\,Te_2$	
Formula weight	2033.26	
Temperature	150(2) K	
Wavelength	1.54184 Å	
Crystal system	Triclinic	
Space group	P-1	
Unit cell dimensions	a = 17.3075(2) Å	α = 85.3740(10)°.
	b = 20.7661(3) Å	β= 89.0520(10)°.
	c = 25.1018(3) Å	γ = 68.0790(10)°.
Volume	8341.38(19) $Å^3$	
Z	4	
Density (calculated)	1.619 Mg/m^3	
Absorption coefficient	11.183 mm^{-1}	
F(000)	3980	
Crystal size	0.400 x 0.400 x 0.400 mm^3	
Theta range for data collection	3.533 to 76.656°.	
Index ranges	-21<=h<=18, -26<=k<=26, -31<=l<=31	
Reflections collected	175477	
Independent reflections	34525 [R(int) = 0.0607]	
Completeness to theta = 67.684°	99.9 %	
Absorption correction	Sphere	
Max. and min. transmission	0.12562 and 0.03228	
Refinement method	Full-matrix least-squares on F^2	
Data / restraints / parameters	34525 / 0 / 1819	
Goodness-of-fit on F^2	1.037	
Final R indices [I>2sigma(I)]	R1 = 0.0630, wR2 = 0.1775	
R indices (all data)	R1 = 0.0693, wR2 = 0.1870	
Extinction coefficient	n/a	
Largest diff. peak and hole	2.456 and -1.469 e.$Å^{-3}$	

Table S26. Crystal data and structure refinement for **1•ChB**^{PFP}•KI.

Identification code	039zy20_sq	
Empirical formula	$C_{53} H_{44} Cl_9 F_{10} I K N_7 O_{12} Te_2$	
Formula weight	1901.20	
Temperature	150(2) K	
Wavelength	1.54178 Å	
Crystal system	Triclinic	
Space group	P-1	
Unit cell dimensions	a = 14.5034(3) Å	α = 118.166(3)°.
	b = 18.4077(5) Å	β = 99.957(2)°.
	c = 18.5490(6) Å	γ = 97.552(2)°.
Volume	4168.5(2) $Å^3$	
Z	2	
Density (calculated)	1.515 Mg/m^3	
Absorption coefficient	12.181 mm^{-1}	
F(000)	1852	
Crystal size	0.2 x 0.2 x 0.2 mm^3	
Theta range for data collection	3.191 to 76.336°.	
Index ranges	-18<=h<=16, -22<=k<=23, -22<=l<=23	
Reflections collected	79897	
Independent reflections	17205 [R(int) = 0.0682]	
Completeness to theta = 67.679°	99.9 %	
Absorption correction	Sphere	
Max. and min. transmission	0.09152 and 0.01392	
Refinement method	Full-matrix least-squares on F^2	
Data / restraints / parameters	17205 / 0 / 856	
Goodness-of-fit on F^2	1.164	
Final R indices [I>2sigma(I)]	R1 = 0.0943, wR2 = 0.2564	
R indices (all data)	R1 = 0.1009, wR2 = 0.2733	
Extinction coefficient	n/a	
Largest diff. peak and hole	3.960 and -1.446 e.$Å^{-3}$	

Table S27. Crystal data and structure refinement for **1•ChB**^{PFP}•RbI.

Identification code	003zy21_sq	
Empirical formula	$C_{52} H_{43} Cl_6 F_{10} I N_7 O_{12} Rb Te_2$	
Formula weight	1828.20	
Temperature	150(2) K	
Wavelength	1.54184 Å	
Crystal system	Triclinic	
Space group	P-1	
Unit cell dimensions	a = 14.4929(2) Å	α = 117.326(2)°.
	b = 18.4361(2) Å	β = 99.9420(10)°.
	c = 18.4714(3) Å	γ = 97.8410(10)°.
Volume	4185.59(12) Å^3	
Z	2	
Density (calculated)	1.451 Mg/m^3	
Absorption coefficient	11.491 mm^{-1}	
F(000)	1772	
Crystal size	0.4 x 0.4 x 0.4 mm^3	
Theta range for data collection	3.194 to 76.226°.	
Index ranges	-17<=h<=18, -23<=k<=23, -23<=l<=21	
Reflections collected	91967	
Independent reflections	17243 [R(int) = 0.0651]	
Completeness to theta = 67.684°	100.0 %	
Absorption correction	Sphere	
Max. and min. transmission	0.08917 and 0.01300	
Refinement method	Full-matrix least-squares on F^2	
Data / restraints / parameters	17243 / 0 / 820	
Goodness-of-fit on F^2	1.064	
Final R indices [I>2sigma(I)]	R1 = 0.0613, wR2 = 0.1602	
R indices (all data)	R1 = 0.0683, wR2 = 0.1700	
Extinction coefficient	n/a	
Largest diff. peak and hole	2.231 and -0.551 e.Å^{-3}	

Table S28. Crystal data and structure refinement for **1•ChB**^{PFP}•CsI.

Identification code	005zy21_sq	
Empirical formula	$C_{50} H_{41} Cs F_{10} I N_7 O_{12} Te_2$	
Formula weight	1636.91	
Temperature	150(2) K	
Wavelength	1.54184 Å	
Crystal system	Triclinic	
Space group	P-1	
Unit cell dimensions	a = 14.4539(3) Å	α = 115.718(2)°.
	b = 18.4545(3) Å	β = 97.669(2)°.
	c = 18.4961(4) Å	γ = 100.608(2)°.
Volume	4240.27(16) Å^3	
Z	2	
Density (calculated)	1.282 Mg/m^3	
Absorption coefficient	12.172 mm^{-1}	
F(000)	1576	
Crystal size	0.400 x 0.400 x 0.400 mm^3	
Theta range for data collection	3.620 to 76.462°.	
Index ranges	-18<=h<=18, -20<=k<=23, -23<=l<=22	
Reflections collected	70615	
Independent reflections	17510 [R(int) = 0.0659]	
Completeness to theta = 67.684°	99.9 %	
Absorption correction	Sphere	
Max. and min. transmission	0.07118 and 0.00693	
Refinement method	Full-matrix least-squares on F^2	
Data / restraints / parameters	17510 / 0 / 748	
Goodness-of-fit on F^2	1.021	
Final R indices [I>2sigma(I)]	R1 = 0.0886, wR2 = 0.2267	
R indices (all data)	R1 = 0.0948, wR2 = 0.2420	
Extinction coefficient	n/a	
Largest diff. peak and hole	4.493 and -0.754 e.Å^{-3}	
